# Supplementary material for: Pregnancy exposure registries for drugs and vaccines in low-income and middle-income countries: scoping review protocol
Source: BMJ Open. 2023 May 8;13(5):e070543. doi: 10.1136/bmjopen-2022-070543 (PMC10174003; doi:10.1136/bmjopen-2022-070543)
Supplement: Supplementary data [file bmjopen-2022-070543supp001.pdf]

Search Strategy by Database:

PubMed

Set #	Query	Results
132	#130 NOT #131	2,016
131	address[Publication Type] OR autobiography[Publication Type] OR bibliography[Publication Type] OR biography[Publication Type] OR comment[Publication Type] OR dictionary[Publication Type] OR directory[Publication Type] OR editorial[Publication Type] OR "expression of concern"[Publication Type] OR festschrift[Publication Type] OR historical article[Publication Type] OR interactive tutorial[Publication Type] OR lecture[Publication Type] OR news[Publication Type] OR newspaper article[Publication Type] OR portrait[Publication Type] OR video-audio media[Publication Type] OR webcast[Publication Type]	2,050,653
130	#128 NOT #129	2,048
129	Animals[mesh] NOT Humans[mesh]	5,016,862
128	#126 AND #127	2,051
127	("2000"[Date - Publication] : "3000"[Date - Publication])	20,721,844
126	#61 AND #125	2,597
125	#62 OR #63 OR #64 OR #65 OR #66 OR #67 OR #68 OR #69 OR #70 OR #71 OR #72 OR #73 OR #74 OR #75 OR #76 OR #77 OR #78 OR #79 OR #80 OR #81 OR #82 OR #83 OR #84 OR #85 OR #86 OR #87 OR #88 OR #89 OR #90 OR #91 OR #92 OR #93 OR #94 OR #95 OR #96 OR #97 OR #98 OR #99 OR #100 OR #101 OR #102 OR #103 OR #104 OR #105 OR #106 OR #107 OR #108 OR #109 OR #110 OR #111 OR #112 OR #113 OR #114 OR #115 OR #116 OR #117 OR #118 OR #119 OR #120 OR #121 OR #122 OR #123 OR #124	7,704,889
124	MNCH[Other Term] AND (maternal*[Other Term] OR newborn*[Other Term] OR child*[Other Term])	11
123	MNCH[Title] AND (maternal*[Title] OR newborn*[Title] OR child*[Title])	22
122	"Maternal Health"[mesh]	2,142
121	"Child Health"[mesh]	4,447
120	"Infant Health"[mesh]	1,175
119	(birth[Other Term] OR births[Other Term]) AND defect*[Other Term]	1,029
118	(birth[Title] OR births[Title]) AND defect*[Title]	2,671

117	(birth[Other Term] OR births[Other Term] OR matern*[Other Term] OR neonat*[Other Term] OR neo-nat*[Other Term] OR perinatal*[Other Term] OR peri-natal*[Other Term] OR peripartum[Other Term] OR "peri-partum"[Other Term] OR postnatal*[Other Term] OR post-natal*[Other Term] OR postpartum[Other Term] OR "post-partum"[Other Term] OR post-birth*[Other Term] OR pregnanc*[Other Term]) AND outcome*[Other Term]	9,426
116	(birth[Title] OR births[Title] OR matern*[Title] OR neonat*[Title] OR neo-nat*[Title] OR perinatal*[Title] OR peri-natal*[Title] OR peripartum[Title] OR "peri-partum"[Title] OR postnatal*[Title] OR post-natal*[Title] OR postpartum[Title] OR "post-partum"[Title] OR post-birth*[Title] OR pregnanc*[Title]) AND outcome*[Title]	31,066
115	Pregnancy Outcome[mesh]	82,242
114	(fetal[OT] OR foetal[OT] OR fetus*[OT] OR foetus*[OT] OR prenatal*[OT] OR pre-natal*[OT]) AND expos*[OT]	2,033
113	(fetal[Title] OR foetal[Title] OR fetus*[Title] OR foetus*[Title] OR prenatal*[Title] OR pre-natal*[Title]) AND expos*[Title]	10,858
112	"Prenatal Exposure Delayed Effects"[mesh]	33,016
111	(fetal[Other Term] OR foetal[Other Term] OR fetus*[Other Term] OR foetus*[Other Term]) AND develop*[Other Term]	2,531
110	(fetal[Title] OR foetal[Title] OR fetus*[Title] OR foetus*[Title]) AND develop*[Title]	8,734
109	"Fetal Development"[mesh:noexp]	9,246
108	antiretroviral*[Other Term] OR anti-retroviral*[Other Term] OR "anti-HIV"[Other Term] OR "anti-AIDS"[Other Term] OR "AIDS drug"[Other Term] OR "AIDS drugs"[Other Term]	7,307
107	(antiretroviral*[Title] OR anti-retroviral*[Title] OR "anti-HIV"[Title] OR "anti-AIDS"[Title] OR "AIDS drug"[Title] OR "AIDS drugs"[Title])	29,826
106	"Anti-Retroviral Agents"[mesh]	69,532
105	"Zika Virus Infection/prevention and control"[MeSH]	1,096
104	"Streptococcal Infections/prevention and control"[MeSH:noexp]	2,944
103	"Respiratory Syncytial Virus Infections/prevention and control"[MeSH Terms]	1,832
102	"Hepatitis E/prevention and control"[MeSH Terms]	346
101	"Cytomegalovirus Infections/prevention and control"[MeSH Terms]	3,130
100	antimalarial*[Other Term] OR anti-malarial*[Other Term]	2,577
99	antimalarial*[Title] OR anti-malarial*[Title]	8,562
98	Antimalarials[mesh]	28,226

97	"Malaria/prevention and control"[MeSH Terms]	17,528
96	(maternal[Other Term] OR pregnan*[Other Term]) AND (immunisation*[Other Term] OR immunization*[Other Term])	754
95	(maternal[Title] OR pregnan*[Title]) AND (immunisation*[Title] OR immunization*[Title])	1,153
94	(antibod*[Other Term] OR anti-bod*[Other Term]) AND transfer*[Other Term]	338
93	(antibod*[Title] OR anti-bod*[Title]) AND transfer*[Title]	1,620
92	immunit*[Other Term] AND maternally-acqui*[Other Term]	8
91	immunit*[Title] AND maternally-acqui*[Title]	8
90	immunit*[Other Term] AND transfer*[Other Term]	161
89	immunit*[Title] AND transfer*[Title]	882
88	"Immunity, Maternally-Acquired"[mesh]	5,842
87	"Immunization Programs"[mesh:noexp]	12,435
86	Immunization[mesh:noexp]	144,668
85	vaccin*[ti] OR vaccin*[ot]	220,223
84	"Viral Vaccines"[mesh]	132,729
83	"Vaccines, Live, Unattenuated"[mesh]	82
82	"Vaccines, Synthetic"[mesh]	31,589
81	"Vaccines, Inactivated"[mesh:noexp]	6,082
80	"Vaccines, Combined"[mesh]	10,562
79	"Vaccines, Attenuated"[mesh]	12,810
78	"Protozoan Vaccines"[mesh]	6,894
77	"Bacterial Vaccines"[mesh]	72,365
76	Vaccines[mesh:noexp]	25,746
75	Vaccination[mesh]	100,718
74	GAIA[Text Word] AND (alignment[Text Word] OR immunisation[Text Word] OR immunization[Text Word] OR safety[Text Word] OR pregnan*[Text Word])	35
73	"Global Alignment of Immunization Safety Assessment in Pregnancy"[tw] - Schema: all	0
72	AEFI[Other Term] AND adverse[Other Term]	68
71	AEFI[Title] AND adverse[Title]	35
70	adverse effect[Other Term] OR adverse effects[Other Term] OR adverse reaction[Other Term] OR adverse reactions[Other Term] OR adverse event[Other Term] OR adverse events[Other Term] OR adverse outcome[Other Term] OR adverse outcomes[Other Term]	17,729
69	adverse effect[Title] OR adverse effects[Title] OR adverse reaction[Title] OR adverse reactions[Title] OR adverse event[Title] OR adverse events[Title] OR adverse outcome[Title] OR adverse outcomes[Title]	31,848

68	safe[Other Term] OR safety[Other Term] OR side effect[Other Term] OR side effects[Other Term] OR undesirable effect[Other Term] OR undesirable effects[Other Term] OR treatment emergent[Other Term] OR tolerability[Other Term] OR toxicity[Other Term] OR adrs[Other Term]	98,171
67	safe[Title] OR safety[Title] OR side effect[Title] OR side effects[Title] OR undesirable effect[Title] OR undesirable effects[Title] OR treatment emergent[Title] OR tolerability[Title] OR toxicity[Title] OR adrs[Title]	296,710
66	("adverse effects"[MeSH Subheading]) OR (Complications[MeSH Subheading]) OR ("drug effects"[MeSH Subheading])	6,963,724
65	(drug[Title] OR drugs[Title] OR medicine[Title] OR medicines[Title] OR medication*[Title] OR pharmaceutical*[Title] OR pharma-ceutical*[Title]) AND expos*[Title]	4,387
64	"Drug-Related Side Effects and Adverse Reactions"[MeSH Terms]	127,437
63	(global[Title] OR international*[Title] OR world*[Title]) AND health*[Title]	27,778
62	Global Health[mesh]	53,845
61	#56 AND #60	8,450
60	#57 OR #58 OR #59	1,104,126
59	"high burden country"[Other Term] OR "high burden countries"[Other Term] OR "high-burden country"[Other Term] OR "high-burden countries"[Other Term] OR "countdown country"[Other Term] OR "countdown countries"[Other Term]	10
58	"high burden country"[Title/Abstract] OR "high burden countries"[Title/Abstract] OR "high-burden country"[Title/Abstract] OR "high-burden countries"[Title/Abstract] OR "countdown country"[Title/Abstract] OR "countdown countries"[Title/Abstract]	633

57	afghanistan[Text Word] OR albania[Text Word] OR algeria[Text Word] OR "american samoa"[Text Word] OR angola[Text Word] OR antigua[Text Word] OR barbuda[Text Word] OR argentina[Text Word] OR armenia[Text Word] OR armenian[Text Word] OR aruba[Text Word] OR azerbaijan[Text Word] OR bahrain[Text Word] OR bangladesh[Text Word] OR barbados[Text Word] OR belarus[Text Word] OR byelarus[Text Word] OR belorussia[Text Word] OR byelorussian[Text Word] OR belize[Text Word] OR "british honduras"[Text Word] OR benin[Text Word] OR dahomey[Text Word] OR bhutan[Text Word] OR bolivia[Text Word] OR "bosnia herzegovina"[Text Word] OR bosnia[Text Word] OR herzegovina[Text Word] OR botswana[Text Word] OR bechuanaland[Text Word] OR brazil[Text Word] OR brasil[Text Word] OR bulgaria[Text Word] OR "burkina faso"[Text Word] OR "burkina fasso"[Text Word] OR "upper volta"[Text Word] OR burundi[Text Word] OR urundi[Text Word] OR "cabo verde"[Text Word] OR "cape verde"[Text Word] OR cambodia[Text Word] OR kampuchea[Text Word] OR "khmer republic"[Text Word] OR cameroon[Text Word] OR cameron[Text Word] OR cameroun[Text Word] OR "central african republic"[Text Word] OR "ubangi shari"[Text Word] OR chad[Text Word] OR chile[Text Word] OR china[Text Word] OR colombia[Text Word] OR comoros[Text Word] OR "comoro islands"[Text Word] OR "iles comores"[Text Word] OR mayotte[Text Word] OR "democratic republic of the congo"[Text Word] OR "democratic republic congo"[Text Word] OR congo[Text Word] OR zaire[Text Word] OR "costa rica"[Text Word] OR "cote d’ivoire"[Text Word] OR "cote d’ ivoire"[Text Word] OR "cote divoire"[Text Word] OR "cote d ivoire"[Text Word] OR "ivory coast"[Text Word] OR croatia[Text Word] OR cuba[Text Word] OR cyprus[Text Word] OR "czech republic"[Text Word] OR czechoslovakia[Text Word] OR djibouti[Text Word] OR "french somaliland"[Text Word] OR dominica[Text Word] OR "dominican republic"[Text Word] OR ecuador[Text Word] OR egypt[Text Word] OR "united arab republic"[Text Word] OR "el salvador"[Text Word] OR "equatorial guinea"[Text Word] OR "spanish guinea"[Text Word] OR eritrea[Text Word] OR estonia[Text Word] OR eswatini[Text Word] OR swaziland[Text Word] OR ethiopia[Text Word] OR fiji[Text Word] OR gabon[Text Word] OR "gabonese republic"[Text Word] OR gambia[Text Word] OR "georgia (republic)"[Text Word] OR georgian[Text Word] OR ghana[Text Word] OR "gold coast"[Text Word] OR gibraltar[Text Word] OR greece[Text Word] OR grenada[Text Word] OR guam[Text Word] OR guatemala[Text Word] OR guinea[Text Word] OR "guinea bissau"[Text Word] OR guyana[Text Word] OR "british guiana"[Text Word] OR haiti[Text Word] OR hispaniola[Text Word] OR honduras[Text Word] OR hungary[Text Word] OR india[Text Word] OR indonesia[Text Word] OR timor[Text Word] OR iran[Text Word] OR iraq[Text Word] OR "isle	1,103,822

of man"[Text Word] OR jamaica[Text Word] OR jordan[Text Word] OR kazakhstan[Text Word] OR kazakh[Text Word] OR kenya[Text Word] OR "democratic people’s republic of korea"[Text Word] OR "republic of korea"[Text Word] OR "north korea"[Text Word] OR "south korea"[Text Word] OR korea[Text Word] OR kosovo[Text Word] OR kyrgyzstan[Text Word] OR kirghizia[Text Word] OR kirgizstan[Text Word] OR "kyrgyz republic"[Text Word] OR kirghiz[Text Word] OR laos[Text Word] OR "lao pdr"[Text Word] OR "lao people's democratic republic"[Text Word] OR latvia[Text Word] OR lebanon[Text Word] OR lebanese republic[Text Word] OR lesotho[Text Word] OR basutoland[Text Word] OR liberia[Text Word] OR libya[Text Word] OR "libyan arab jamahiriya"[Text Word] OR lithuania[Text Word] OR macau[Text Word] OR macao[Text Word] OR republic of "north macedonia"[Text Word] OR macedonia[Text Word] OR madagascar[Text Word] OR "malagasy republic"[Text Word] OR malawi[Text Word] OR nyasaland[Text Word] OR malaysia[Text Word] OR "malay federation"[Text Word] OR "malaya federation"[Text Word] OR maldives[Text Word] OR "indian ocean islands"[Text Word] OR "indian ocean"[Text Word] OR mali[Text Word] OR malta[Text Word] OR micronesia[Text Word] OR "federated states of micronesia"[Text Word] OR kiribati[Text Word] OR "marshall islands"[Text Word] OR nauru[Text Word] OR "northern mariana islands"[Text Word] OR palau[Text Word] OR tuvalu[Text Word] OR mauritania[Text Word] OR mauritius[Text Word] OR mexico[Text Word] OR moldova[Text Word] OR moldovian[Text Word] OR mongolia[Text Word] OR montenegro[Text Word] OR morocco[Text Word] OR ifni[Text Word] OR mozambique[Text Word] OR "portuguese east africa"[Text Word] OR myanmar[Text Word] OR burma[Text Word] OR namibia[Text Word] OR nepal[Text Word] OR "netherlands antilles"[Text Word] OR nicaragua[Text Word] OR niger[Text Word] OR nigeria[Text Word] OR oman[Text Word] OR muscat[Text Word] OR pakistan[Text Word] OR panama[Text Word] OR "papua new guinea"[Text Word] OR "new guinea"[Text Word] OR paraguay[Text Word] OR peru[Text Word] OR philippines[Text Word] OR philipines[Text Word] OR phillipines[Text Word] OR phillippines[Text Word] OR poland[Text Word] OR "polish people's republic"[Text Word] OR portugal[Text Word] OR "portuguese republic"[Text Word] OR "puerto rico"[Text Word] OR romania[Text Word] OR russia[Text Word] OR "russian federation"[Text Word] OR ussr[Text Word] OR "soviet union"[Text Word] OR "union of soviet socialist republics"[Text Word] OR rwanda[Text Word] OR ruanda[Text Word] OR samoa[Text Word] OR "pacific islands"[Text Word] OR polynesia[Text Word] OR "samoan islands"[Text Word] OR "navigator island"[Text Word] OR "navigator islands"[Text Word] OR "sao tome and principe"[Text Word] OR "saudi arabia"[Text Word] OR senegal[Text Word] OR serbia[Text Word] OR

seychelles[Text Word] OR "sierra leone"[Text Word] OR slovakia[Text Word] OR "slovak republic"[Text Word] OR slovenia[Text Word] OR melanesia[Text Word] OR "solomon island"[Text Word] OR "solomon islands"[Text Word] OR "norfolk island"[Text Word] OR "norfolk islands"[Text Word] OR somalia[Text Word] OR "south africa"[Text Word] OR "south sudan"[Text Word] OR "sri lanka"[Text Word] OR ceylon[Text Word] OR "saint kitts and nevis"[Text Word] OR "st. kitts and nevis"[Text Word] OR "saint lucia"[Text Word] OR "st. lucia"[Text Word] OR "saint Vincent and the grenadines"[Text Word] OR "saint vincent"[Text Word] OR "st. vincent"[Text Word] OR grenadines[Text Word] OR sudan[Text Word] OR suriname[Text Word] OR surinam[Text Word] OR "dutch guiana"[Text Word] OR "netherlands guiana"[Text Word] OR syria[Text Word] OR "syrian arab republic"[Text Word] OR tajikistan[Text Word] OR tadjikistan[Text Word] OR tadzhikistan[Text Word] OR tadzhik[Text Word] OR tanzania[Text Word] OR tanganyika[Text Word] OR thailand[Text Word] OR siam[Text Word] OR "timor leste"[Text Word] OR "east timor"[Text Word] OR togo[Text Word] OR "togolese republic"[Text Word] OR tonga[Text Word] OR "Trinidad and tobago"[Text Word] OR trinidad[Text Word] OR tobago[Text Word] OR tunisia[Text Word] OR turkey[Text Word] OR turkmenistan[Text Word] OR turkmen[Text Word] OR uganda[Text Word] OR ukraine[Text Word] OR uruguay[Text Word] OR uzbekistan[Text Word] OR uzbek[Text Word] OR vanuatu[Text Word] OR "new hebrides"[Text Word] OR venezuela[Text Word] OR vietnam[Text Word] OR "viet nam"[Text Word] OR "middle east"[Text Word] OR "west bank"[Text Word] OR gaza[Text Word] OR palestine[Text Word] OR yemen[Text Word] OR yugoslavia[Text Word] OR zambia[Text Word] OR zimbabwe[Text Word] OR "northern rhodesia"[Text Word] OR "global south"[Text Word] OR "africa south of the sahara"[Text Word] OR "sub-saharan africa"[Text Word] OR "subsaharan africa"[Text Word] OR "africa, central"[Text Word] OR "central africa"[Text Word] OR "africa, northern"[Text Word] OR "north africa"[Text Word] OR "northern africa"[Text Word] OR magreb[Text Word] OR maghrib[Text Word] OR sahara[Text Word] OR "africa, southern"[Text Word] OR "southern africa"[Text Word] OR "africa, eastern"[Text Word] OR "east africa"[Text Word] OR "eastern africa"[Text Word] OR "africa, western"[Text Word] OR "west africa"[Text Word] OR "western africa"[Text Word] OR "west indies"[Text Word] OR caribbean[Text Word] OR "central america"[Text Word] OR "latin america"[Text Word] OR "south and central america"[Text Word] OR "south america"[Text Word] OR "asia, central"[Text Word] OR "central asia"[Text Word] OR "asia, northern"[Text Word] OR "north asia"[Text Word] OR "northern asia"[Text Word] OR "asia, southeastern"[Text Word] OR "southeastern asia"[Text Word] OR

"south eastern asia"[Text Word] OR "southeast asia"[Text Word] OR "south east asia"[Text Word] OR "asia, western"[Text Word] OR "western asia"[Text Word] OR "europe, eastern"[Text Word] OR "east europe"[Text Word] OR "eastern europe"[Text Word] OR "developing country"[Text Word] OR "developing countries"[Text Word] OR "developing nation"[Text Word] OR "developing nations"[Text Word] OR "developing population"[Text Word] OR "developing populations"[Text Word] OR "developing world"[Text Word] OR "less developed country"[Text Word] OR "less developed countries"[Text Word] OR "less developed nation"[Text Word] OR "less developed nations"[Text Word] OR "less developed population"[Text Word] OR "less developed populations"[Text Word] OR "less developed world"[Text Word] OR "lesser developed country"[Text Word] OR "lesser developed countries"[Text Word] OR "lesser developed nation"[Text Word] OR "lesser developed nations"[Text Word] OR "lesser developed population"[Text Word] OR "lesser developed populations"[Text Word] OR "lesser developed world"[Text Word] OR "under developed country"[Text Word] OR "under developed countries"[Text Word] OR "under developed nation"[Text Word] OR "under developed nations"[Text Word] OR "under developed population"[Text Word] OR "under developed populations"[Text Word] OR "under developed world"[Text Word] OR "underdeveloped country"[Text Word] OR "underdeveloped countries"[Text Word] OR "underdeveloped nation"[Text Word] OR "underdeveloped nations"[Text Word] OR "underdeveloped population"[Text Word] OR "underdeveloped populations"[Text Word] OR "underdeveloped world"[Text Word] OR "middle income country"[Text Word] OR "middle income countries"[Text Word] OR "middle income nation"[Text Word] OR "middle income nations"[Text Word] OR "middle income population"[Text Word] OR "middle income populations"[Text Word] OR "low income country"[Text Word] OR "low income countries"[Text Word] OR "low income nation"[Text Word] OR "low income nations"[Text Word] OR "low income population"[Text Word] OR "low income populations"[Text Word] OR "lower income country"[Text Word] OR "lower income countries"[Text Word] OR "lower income nation"[Text Word] OR "lower income nations"[Text Word] OR "lower income population"[Text Word] OR "lower income populations"[Text Word] OR "underserved country"[Text Word] OR "underserved countries"[Text Word] OR "underserved nation"[Text Word] OR "underserved nations"[Text Word] OR "underserved population"[Text Word] OR "underserved populations"[Text Word] OR "underserved world"[Text Word] OR "under served country"[Text Word] OR "under served countries"[Text Word] OR "under served nation"[Text Word] OR "under served nations"[Text Word] OR "under served population"[Text Word] OR "under served populations"[Text

	Word] OR "under served world"[Text Word] OR "deprived country"[Text Word] OR "deprived countries"[Text Word] OR "deprived nation"[Text Word] OR "deprived nations"[Text Word] OR "deprived population"[Text Word] OR "deprived populations"[Text Word] OR "deprived world"[Text Word] OR "poor country"[Text Word] OR "poor countries"[Text Word] OR "poor nation"[Text Word] OR "poor nations"[Text Word] OR "poor population"[Text Word] OR "poor populations"[Text Word] OR "poor world"[Text Word] OR "poorer country"[Text Word] OR "poorer countries"[Text Word] OR "poorer nation"[Text Word] OR "poorer nations"[Text Word] OR "poorer population"[Text Word] OR "poorer populations"[Text Word] OR "poorer world"[Text Word] OR "developing economy"[Text Word] OR "developing economies"[Text Word] OR "less developed economy"[Text Word] OR "less developed economics"[Text Word] OR "lesser developed economy"[Text Word] OR "lesser developed economies"[Text Word] OR "under developed economy"[Text Word] OR "under developed economies"[Text Word] OR "underdeveloped economy"[Text Word] OR "underdeveloped economies"[Text Word] OR "middle income economy"[Text Word] OR "middle income economies"[Text Word] OR "low income economy"[Text Word] OR "low income economies"[Text Word] OR "lower income economy"[Text Word] OR "lower income economies"[Text Word] OR "low gdp"[Text Word] OR "low gnp"[Text Word] OR "low gross domestic"[Text Word] OR "low gross national"[Text Word] OR "lower gdp"[Text Word] OR "lower gnp"[Text Word] OR "lower gross domestic"[Text Word] OR "lower gross national"[Text Word] OR lmic[Text Word] OR lmics[Text Word] OR "third world"[Text Word] OR "lami country"[Text Word] OR "lami countries"[Text Word] OR "transitional country"[Text Word] OR "transitional economies"[Text Word] OR "emerging economy"[Text Word] OR "emerging economies"[Text Word] OR "emerging nation"[Text Word] OR "emerging nations"[Text Word]	
56	#1 OR #55	39,866
55	#21 AND #54	39,859
54	#22 OR #23 OR #24 OR #25 OR #26 OR #27 OR #28 OR #29 OR #30 OR #31 OR #32 OR #33 OR #34 OR #35 OR #36 OR #37 OR #38 OR #39 OR #40 OR #41 OR #42 OR #43 OR #44 OR #45 OR #46 OR #47 OR #48 OR #49 OR #50 OR #51 OR #52 OR #53	850,027
53	"Product Surveillance, Postmarketing/statistics and numerical data"[MeSH Terms]	2,361
52	"Maternal Health Services/statistics and numerical data"[MeSH Terms]	7,290
51	"Maternal Exposure/statistics and numerical data"[MeSH Terms]	1,123

50	pharmacovigilan*[ti] OR pharmaco-vigilan*[ti] OR pharmacovigilan*[ot] OR pharmaco-vigilan*[ot]	3,751
49	"Product Surveillance, Postmarketing"[mesh:noexp]	7,553
48	"data system"[ti] OR "data systems"[ti] OR "information system"[ti] OR "information systems"[ti] OR "data system"[ot] OR "data systems"[ot] OR "information system"[ot] OR "information systems"[ot]	13,581
47	"Health Information Systems"[mesh]	1,546
46	(decision*[ti] AND support*[ti] AND clinical*[ti]) OR (decision*[ot] AND support*[ot] AND clinical*[ot])	4,467
45	"Decision Support Systems, Clinical"[mesh]	9,106
44	"Databases, Factual"[mesh]	163,862
43	"Databases as Topic"[mesh:noexp]	9,688
42	surveillance*[ti] OR surveillance*[ot]	61,304
41	"Population Surveillance"[mesh]	74,026
40	survey*[ti] OR survey*[ot]	189,840
39	"Health Surveys"[mesh:noexp]	66,266
38	"Health Care Surveys"[MeSH Major Topic]	10,167
37	"Surveys and Questionnaires"[MeSH Major Topic:noexp]	49,876
36	registry[ot] OR registries[ot] OR eregistr*[ot] OR "e-registry"[ot] OR "e-registries"[ot]	8,509
35	registry[ti] OR registries[ti] OR eregistr*[ti] OR "e-registry"[ti] OR "e-registries"[ti]	34,311
34	Registries[mesh:noexp]	104,749
33	(preliminary[ot] OR "pilot project"[ot] OR "pilot projects"[ot]) AND data[ot]	35
32	(preliminary[ti] OR "pilot project"[ti] OR "pilot projects"[ti]) AND data[ti]	2,947
31	"Preliminary Data"[mesh]	724
30	"focus group"[ti] OR "focus groups"[ti] OR "focus group"[ot] OR "focus groups"[ot]	6,048
29	"Focus Groups"[mesh]	34,384
28	databas*[ot] OR "data base"[ot] OR "data bases"[ot] OR databank*[ot] OR "data bank"[ot] OR "data banks"[ot] OR dataset*[ot] OR "data set"[ot] OR "data sets"[ot]	14,502
27	databas*[ti] OR "data base"[ti] OR "data bases"[ti] OR databank*[ti] OR "data bank"[ti] OR "data banks"[ti] OR dataset*[ti] OR "data set"[ti] OR "data sets"[ti]	55,119
26	"Datasets as Topic"[mesh]	7,252
25	data[ot] AND (accumulat*[ot] OR accura*[ot] OR assembl*[ot] OR captur*[ot] OR collect*[ot] OR compil*[ot] OR coordinat*[ot] OR co-ordinat*[ot] OR gather*[ot] OR hub[ot] OR hubs[ot])	5,205

24	data[ti] AND (accumulat*[ti] OR accura*[ti] OR assembl*[ti] OR captur*[ti] OR collect*[ti] OR compil*[ti] OR coordinat*[ti] OR co-ordinat*[ti] OR gather*[ti] OR hub[ti] OR hubs[ti])	11,365
23	"Data Accuracy"[mesh]	3,726
22	"Data Collection"[mesh:noexp]	91,761
21	#2 OR #3 OR #4 OR #5 OR #6 OR #7 OR #8 OR #9 OR #10 OR #11 OR #12 OR #13 OR #14 OR #15 OR #16 OR #17 OR #18 OR #19 OR #20	1,067,642
20	maternal[ot] AND (fetal[ot] OR fetus[ot] OR foetal[ot] OR foetus[ot])	3,848
19	(maternal*[ot] OR maternity[ot]) AND (care[ot] OR health*[ot] OR service[ot] OR services[ot])	11,210
18	(maternal*[ot] or maternity[ot]) AND (contact*[ot] or expos*[ot])	601
17	perinatal*[ot] OR "peri-natal"[ot] OR "peri-natally"[ot] OR peripartum[ot] or "peri-partum"[ot]	7,552
16	prenatal*[ot] OR antenatal*[ot] OR "ante-natal"[ot] OR "ante-natally"[ot] OR antepartum[ot] or "ante-partum"[ot]	15,862
15	maternal[ti] AND (fetal[ti] OR fetus[ti] OR foetal[ti] OR foetus[ti])	13,964
14	(maternal*[ti] OR maternity[ti]) AND (care[ti] OR health*[ti] OR service[ti] OR services[ti])	13,467
13	"Maternal Health Services"[mesh:noexp]	15,617
12	(maternal*[ti] or maternity[ti]) AND (contact*[ti] or expos*[ti])	3,847
11	"Maternal Exposure"[mesh]	10,689
10	perinatal*[ti] OR "peri-natal"[ti] OR "peri-natally"[ti] OR peripartum[ti] or "peri-partum"[ti]	30,982
9	"Perinatal Care"[mesh]	11,334
8	prenatal*[ti] OR antenatal*[ti] OR "ante-natal"[ti] OR "ante-natally"[ti] OR antepartum[ti] or "ante-partum"[ti]	59,414
7	"Prenatal Care"[mesh]	31,249
6	pregnan*[ti]	252,201
5	"Pregnancy Trimesters"[mesh]	43,874
4	"Pregnant Women"[mesh]	12,334
3	"Pregnancy Complications"[mesh]	460,551
2	Pregnancy[mesh]	970,546
1	"pregnancy exposure database" [tw] OR "pregnancy exposure databases" [tw] OR "pregnancy exposure data base" [tw] OR "pregnancy exposure data bases" [tw] OR "pregnancy exposure registry" [tw] OR "pregnancy exposure registries" [tw]	34

Embase

----
1 ("pregnancy exposure database" or "pregnancy exposure databases" or "pregnancy exposure data base" or "pregnancy exposure data bases" or "pregnancy exposure registry" or "pregnancy exposure registries").tw,kw,kf. (113)
2 exp pregnancy/ (727815)
3 exp pregnancy complication/ (141579)
4 exp named groups by pregnancy/ (130431)
5 pregnan*.ti,kw,kf. (329712)
6 exp prenatal care/ (167165)
7 (prenatal* or antenatal* or "ante-natal" or "ante-natally" or antepartum or "ante-partum").ti,kw,kf. (87488)
8 exp perinatal care/ (65235)
9 (perinatal* or peri-natal* or peripartum or "peri-partum").ti,kw,kf. (46195)
10 maternal exposure/ (3606)
11 ((maternal* or maternity) and (contact* or expos*)).ti,kw,kf. (6357)
12 maternal health service/ (2454)
13 ((maternal* or maternity) and (care or health* or service or services)).ti,kw,kf. (22738)
14 (maternal and (fetal or fetus or foetal or foetus)).ti,kw,kf. (20741)
15 or/2-14 [PREGNANCY] (1035932)
16 *information processing/ (37078)
17 data aggregation/ (374)
18 data accuracy/ (1596)
19 (data and (accumulat* or accura* or assembl* or captur* or collect* or compil* or coordinat* or co-ordinat* or gather* or hub or hubs)).ti,kw,kf. (18259)
20 (databas* or "data base" or "data bases" or databank* or "data bank" or "data banks" or dataset* or "data set" or "data sets").ti,kw,kf. (86633)
21 (focus group or focus groups).ti,kw,kf. (7608)
22 preliminary data/ (31472)
23 ((preliminary or pilot project or pilot projects) and data).ti,kw,kf. (5481)
24 exp register/ (180900)
25 (registry or registries or eregistr* or "e-registry" or "e-registries").ti,kw,kf. (68347)
26 questionnaire/ (814172)
27 *health care survey/ (2606)
28 health survey/ (213987)
29 survey*.ti,kw,kf. (220889)
30 population surveillance/ (117)
31 surveillance*.ti,kw,kf. (82221)
32 data base/ (248533)
33 factual database/ (27912)
34 clinical decision support system/ (4716)
35 (decision* and support* and clinical*).ti,kw,kf. (5904)
36 medical information system/ (22591)
37 ("data system" or "data systems" or "information system" or "information systems").ti,kw,kf. (18222)
38 exp postmarketing surveillance/ (37924)
39 (pharmacovigilan* or pharmaco-vigilan*).ti,kw,kf. (7417)

40 or/16-39 [DATA COLLECTION, REGISTRIES] (1815231)
41 15 and 40 [PREGNANCY - DATA COLLECTION, REGISTRIES] (71373)
42 1 or 41 [PERs, PREGNANCY - DATA COLLECTION, REGISTRIES] (71381)
43 (afghanistan or albania or algeria or american samoa or angola or "antigua and barbuda" or antigua or barbuda or argentina or armenia or armenian or aruba or azerbaijan or bahrain or bangladesh or barbados or republic of belarus or belarus or byelarus or belorussia or byelorussian or belize or british honduras or benin or dahomey or bhutan or bolivia or "bosnia and herzegovina" or bosnia or herzegovina or botswana or bechuanaland or brazil or brasil or bulgaria or burkina faso or burkina fasso or upper volta or burundi or urundi or cabo verde or cape verde or cambodia or kampuchea or khmer republic or cameroon or cameron or cameroun or central african republic or ubangi shari or chad or chile or china or colombia or comoros or comoro islands or iles comores or mayotte or democratic republic of the congo or democratic republic congo or congo or zaire or costa rica or "cote d'ivoire" or "cote d' ivoire" or cote divoire or cote d ivoire or ivory coast or croatia or cuba or cyprus or czech republic or czechoslovakia or djibouti or french somaliland or dominica or dominican republic or ecuador or egypt or united arab republic or el salvador or equatorial guinea or spanish guinea or eritrea or estonia or eswatini or swaziland or ethiopia or fiji or gabon or gabonese republic or gambia or "georgia (republic)" or georgian or ghana or gold coast or gibraltar or greece or grenada or guam or guatemala or guinea or guinea bissau or guyana or british guiana or haiti or hispaniola or honduras or hungary or india or indonesia or timor or iran or iraq or isle of man or jamaica or jordan or kazakhstan or kazakh or kenya or "democratic people's republic of korea" or republic of korea or north korea or south korea or korea or kosovo or kyrgyzstan or kirghizia or kirgizstan or kyrgyz republic or kirghiz or laos or lao pdr or "lao people's democratic republic" or latvia or lebanon or lebanese republic or lesotho or basutoland or liberia or libya or libyan arab jamahiriya or lithuania or macau or macao or republic of north macedonia or macedonia or madagascar or malagasy republic or malawi or nyasaland or malaysia or malay federation or malaya federation or maldives or indian ocean islands or indian ocean or mali or malta or micronesia or federated states of micronesia or kiribati or marshall islands or nauru or northern mariana islands or palau or tuvalu or mauritania or mauritius or mexico or moldova or moldovian or mongolia or montenegro or "montenegro (republic)" or morocco or ifni or mozambique or portuguese east africa or myanmar or burma or namibia or nepal or netherlands antilles or nicaragua or niger or nigeria or oman or muscat or pakistan or panama or papua new guinea or new guinea or paraguay or peru or philippines or philipines or phillipines or phillippines or poland or "polish people's republic" or portugal or portuguese republic or puerto rico or romania or russia or russian federation or ussr or soviet union or union of soviet socialist republics or rwanda or ruanda or samoa or pacific islands or polynesia or samoan islands or navigator island or navigator islands or "sao tome and principe" or saudi arabia or senegal or serbia or seychelles or sierra leone or slovakia or slovak republic or slovenia or melanesia or solomon island or solomon islands or norfolk island or norfolk islands or somalia or south africa or south sudan or sri lanka or ceylon or "saint kitts and nevis" or "st. kitts and nevis" or saint lucia or "st. lucia" or "saint vincent and the grenadines" or saint vincent or "st. vincent" or grenadines or sudan or suriname or surinam or dutch guiana or netherlands guiana or syria or syrian arab republic or tajikistan or tadjikistan or tadzhikistan or tadzhik or tanzania or tanganyika or thailand or siam or timor leste or east timor or togo or togolese republic or tonga or "trinidad and tobago" or trinidad or tobago or tunisia or "turkey (republic)" or turkey or turkmenistan or turkmen or uganda or ukraine or uruguay or uzbekistan or uzbek or vanuatu or new hebrides or venezuela or vietnam or viet nam or middle east or west bank or gaza or palestine or yemen or yugoslavia or zambia or zimbabwe or northern rhodesia or global south or africa south of the sahara or "sub saharan africa" or subsaharan africa or africa, central or central africa or africa, northern or north africa or northern africa or magreb or maghrib or sahara or africa, southern or southern africa or africa, eastern or east africa or eastern africa or africa, western or west africa or western africa or west indies or indian ocean islands or caribbean region or

caribbean islands or caribbean or central america or latin america or "south and central america" or south america or asia, central or central asia or asia, northern or north asia or northern asia or asia, southeastern or southeastern asia or south eastern asia or southeast asia or south east asia or asia, western or western asia or europe, eastern or east europe or eastern europe or developing country or developing countries or developing nation? or developing population? or developing world or less developed countr* or less developed nation? or less developed population? or less developed world or lesser developed countr* or lesser developed nation? or lesser developed population? or lesser developed world or under developed countr* or under developed nation? or under developed population? or under developed world or underdeveloped countr* or underdeveloped nation? or underdeveloped population? or underdeveloped world or middle income countr* or middle income nation? or middle income population? or low income countr* or low income nation? or low income population? or lower income countr* or lower income nation? or lower income population? or underserved countr* or underserved nation? or underserved population? or underserved world or under served countr* or under served nation? or under served population? or under served world or deprived countr* or deprived nation? or deprived population? or deprived world or poor countr* or poor nation? or poor population? or poor world or poorer countr* or poorer nation? or poorer population? or poorer world or developing econom* or less developed econom* or lesser developed econom* or under developed econom* or underdeveloped econom* or middle income econom* or low income econom* or lower income econom* or low gdp or low gnp or low gross domestic or low gross national or lower gdp or lower gnp or lower gross domestic or lower gross national or lmic or lmics or third world or lami countr* or transitional countr* or emerging economies or emerging nation?).ti,ab,sh,kw. (2517925)

44 ("high burden country" or "high burden countries" or "high-burden country" or "high-burden countries" or "countdown country" or "countdown countries").ti,ab,kw,kf. (808)

45 43 or 44 [LMICs] (2518149)

46 42 and 45 [PERs, PREGNANCY - DATA COLLECTION, REGISTRIES - LMICs] (18846)

47 global health/ (16219)

48 ((global or international* or world*) and health*).ti,kw,kf. (45947)

49 exp adverse drug reaction/ (586248)

50 ((drug or drugs or medicine or medicines or medication* or pharmaceutical* or pharma-ceutical*) and expos*).ti,kw,kf. (9329)

51 (ae or co).fs. (2915988)

52 (safe or safety or side effect or side effects or undesirable effect or undesirable effects or treatment emergent or tolerability or toxicity or adrs).ti,kw,kf. (514988)

53 (adverse effect or adverse effects or adverse reaction or adverse reactions or adverse event or adverse events or adverse outcome or adverse outcomes).ti,kw,kf. (70731)

54 (AEFI and adverse).ti,kw,kf. (157)

55 "Global Alignment of Immuni#ation Safety Assessment in Pregnancy".tw,kw,kf. (20)

56 (GAIA and (alignment or immuni#ation or safety or pregnan*)).tw,kw,kf. (71)

57 exp vaccination/ (205483)

58 vaccine/ (67719)

59 exp bacterial vaccine/ or exp cell-based vaccine/ or conjugate vaccine/ or edible vaccine/ or exp fungus vaccine/ or exp inactivated vaccine/ or live vaccine/ or exp meningitis vaccine/ or exp nucleic acid vaccine/ or exp parasite vaccine/ or exp peptide vaccine/ or protein vaccine/ or exp subunit vaccine/ or exp toxoid vaccine/ or exp vector vaccine/ or virosome vaccine/ or exp virus vaccine/ (304544)

60 vaccin*.ti,kw,kf. (251220)

61 immunization/ (104541)

62 passive immunization/ (12623)
63 (immunit* and transfer*).ti,kw,kf. (1262)
64 (immunit* and (maternally-acqui* or passive*)).ti,kw,kf. (766)
65 ((antibod* or anti-bod*) and transfer*).ti,kw,kf. (2375)
66 ((maternal or pregnan*) and immuni#ation*).ti,kw,kf. (1766)
67 exp malaria/pc [Prevention] (14654)
68 exp antimalarial agent/ (161880)
69 (antimalarial* or anti-malarial*).ti,kw,kf. (11586)
70 exp cytomegalovirus infection/pc [Prevention] (5146)
71 respiratory syncytial virus infection/pc [Prevention] (1057)
72 exp Streptococcus infection/pc [Prevention] (10568)
73 exp Zika fever/pc [Prevention] (780)
74 exp antiretrovirus agent/ (216196)
75 (antiretroviral* or anti-retroviral* or "anti-HIV" or "anti-AIDS" or "AIDS drug" or "AIDS drugs").ti,kw,kf. (46286)
76 exp fetus development/ (29871)
77 ((fetal or foetal or fetus* or foetus*) and develop*).ti,kw,kf. (14926)
78 exp prenatal exposure/ (37294)
79 ((fetal or foetal or fetus* or foetus* or prenatal* or pre-natal*) and expos*).ti,kw,kf. (16619)
80 pregnancy outcome/ (72177)
81 ((birth or births or matern* or neonat* or neo-nat* or perinatal* or peri-natal*or peripartum or "peri-partum" or postnatal* or post-natal* or postpartum or "post-partum" or post-birth* or pregnanc*) and outcome*).ti,kw,kf. (55800)
82 ((birth or births) and defect*).ti,kw,kf. (4849)
83 child health/ (32206)
84 maternal welfare/ (15842)
85 (MNCH and (maternal* or newborn* or child*)).ti,kw,kf. (49)
86 or/47-85 [DRUGS, VACCINES, SAFETY, OUTCOMES] (4592324)
87 46 and 86 [PERs, PREGNANCY - DATA COLLECTION, REGISTRIES - LMICs - DRUGS, VACCINES, SAFETY, OUTCOMES] (5663)
88 exp animal/ or exp animal experimentation/ or exp animal model/ or exp animal experiment/ or nonhuman/ or exp vertebrate/ (30677570)
89 exp human/ or exp human experimentation/ or exp human experiment/ (23737031)
90 88 not 89 (6941713)
91 87 not 90 [ANIMAL-ONLY REMOVED] (5643)
92 editorial.pt. (729310)
93 91 not 92 [EDITORIALS REMOVED] (5614)
94 limit 93 to yr="2000-current" (5299)

****

CINAHL

#	Query	Results
S92	S90 and S91	878
S91	DT 2000 - 2022	7,386,209

S90	S88 NOT S89	885
S89	PT editorial or opinion or commentary	684,750
S88	S45 AND S87	891
S87	S46 OR S47 OR S48 OR S49 OR S50 OR S51 OR S52 OR S53 OR S54 OR S55 OR S56 OR S57 OR S58 OR S59 OR S60 OR S61 OR S62 OR S63 OR S64 OR S65 OR S66 OR S67 OR S68 OR S69 OR S70 OR S71 OR S72 OR S73 OR S74 OR S75 OR S76 OR S77 OR S78 OR S79 OR S80 OR S81 OR S82 OR S83 OR S84 OR S85 OR S86	354,157
S86	TI (MNCH AND (maternal* OR newborn* OR child*))	17
S85	(MH "Maternal-Child Health")	3,727
S84	(MH "Child Health")	17,073
S83	TI (birth OR births) and defect*	969
S82	TI (birth OR births OR matern* OR neonat* OR neo-nat* OR perinatal* OR peri-natal* OR peripartum OR "peri-partum" OR postnatal* OR post-natal* OR postpartum OR "post-partum" OR post-birth* OR pregnanc*) AND outcome*	17,343
S81	(MH "Pregnancy Outcomes")	27,127
S80	TI (fetal OR foetal OR fetus* OR foetus* or prenatal* or pre-natal*) AND expos*	2,603
S79	(MH "Prenatal Exposure Delayed Effects")	6,314
S78	TI (fetal OR foetal OR fetus* OR foetus*) AND develop*	1,028
S77	(MH "Fetal Development")	7,851
S76	TI antiretroviral* OR anti-retroviral* OR "anti-HIV" OR "anti-AIDS" OR "AIDS drug" OR "AIDS drugs"	9,126
S75	(MH "Anti-Retroviral Agents+")	24,911
S74	(MH "Zika Virus Infections/PC")	168
S73	(MH "Streptococcal Infections+/PC")	2,280
S72	(MH "Respiratory Syncytial Virus Infections/PC")	583
S71	(MH "Hepatitis E/PC")	69
S70	(MH "Cytomegalovirus Infections+/PC")	509
S69	TI antimalarial* or (anti W0 malarial*)	714
S68	(MH "Antimalarials+")	8,320
S67	(MH "Malaria/PC")	3,293

S66	TI (maternal* or pregnan*) and immuni?ation*	253
S65	TI ((antibod* or (anti W0 bod*)) and transfer*)	79
S64	TI immunit* AND (passive* or "maternally-acquired")	13
S63	TI immunit* AND transfer*	16
S62	(MH "Immunity, Maternally Acquired")	232
S61	(MH "Immunization Programs")	6,426
S60	(MH "Viral Vaccines+")	36,206
S59	(MH "Vaccines, Combined+")	3,562
S58	(MH "Toxoids+")	2,372
S57	(MH "Bacterial Vaccines+")	10,296
S56	(MH "Vaccines")	9,533
S55	(MH "Immunization+")	31,914
S54	TI (GAIA AND (alignment or immuni?ation or safety or pregnan*)) OR AB (GAIA AND (alignment or immuni?ation or safety or pregnan*))	6
S53	TI ("Global Alignment of Immunisation Safety Assessment in Pregnancy" or "Global Alignment of Immunization Safety Assessment in Pregnancy") OR AB ("Global Alignment of Immunisation Safety Assessment in Pregnancy" or "Global Alignment of Immunization Safety Assessment in Pregnancy")	0
S52	TI AEFI and adverse	5
S51	TI "adverse effect" or "adverse effects" or "adverse reaction" or "adverse reactions" or "adverse event" or "adverse events" or "adverse outcome" or "adverse outcomes"	12,615
S50	TI safe or safety or "side effect" or "side effects" or "undesirable effect" or "undesirable effects" or "treatment emergent" or tolerability or toxicity or adrs	113,937
S49	TI (drug or drugs or medicine or medicines or medication* or pharmaceutical* or pharma-ceutical*) AND expos*	1,420
S48	(MH "Adverse Drug Event")	16,207
S47	TI (global or international* or world*) and health*	22,194
S46	(MH "World Health")	29,391
S45	S39 AND S44	4,402
S44	S40 OR S41 OR S42 OR S43	379,212

S43	TI ("high burden country" or "high burden countries" or "high-burden country" or "high-burden countries" or "countdown country" or "countdown countries") OR AB ("high burden country" or "high burden countries" or "high-burden country" or "high-burden countries" or "countdown country" or "countdown countries")	188
S42	TI ("developing country" or "developing countries" or "developing nation" or "developing nations" or "developing population" or "developing populations" or "developing world" or "less developed country" or "less developed countries" or "less developed nation" or "less developed nations" or "less developed population" or "less developed populations" or "less developed world" or "lesser developed country" or "lesser developed countries" or "lesser developed nation" or "lesser developed nations" or "lesser developed population" or "lesser developed populations" or "lesser developed world" or "under developed country" or "under developed countries" or "under developed nation" or "under developed nations" or "under developed population" or "under developed populations" or "under developed world" or "underdeveloped country" or "underdeveloped countries" or "underdeveloped nation" or "underdeveloped nations" or "underdeveloped population" or "underdeveloped populations" or "underdeveloped world" or "middle income country" or "middle income countries" or "middle income nation" or "middle income nations" or "middle income population" or "middle income populations" or "low income country" or "low income countries" or "low income nation" or "low income nations" or "low income population" or "low income populations" or "lower income country" or "lower income countries" or "lower income nation" or "lower income nations" or "lower income population" or "lower income populations" or "underserved country" or "underserved countries" or "underserved nation" or "underserved nations" or "underserved population" or "underserved populations" or "underserved world" or "under served country" or "under served countries" or "under served nation" or "under served nations" or "under served population" or "under served populations" or "under served world" or "deprived country" or "deprived countries" or "deprived nation" or "deprived nations" or "deprived population" or "deprived populations" or "deprived world" or "poor country" or "poor countries" or "poor nation" or "poor nations" or "poor population" or "poor populations" or "poor world" or "poorer country" or "poorer countries" or "poorer nation" or "poorer nations" or "poorer population" or "poorer populations" or "poorer world" or "developing economy" or "developing economies" or "less developed economy" or "less developed economics" or "lesser developed economy" or "lesser developed economies" or "under developed economy" or "under developed economies" or "underdeveloped economy" or "underdeveloped economies" or "middle income economy" or "middle income economies" or "low income economy" or "low income economies" or "lower income economy" or "lower income economies" or "low gdp" or "low gnp" or "low gross domestic" or "low gross national" or "lower gdp" or "lower gnp" or "lower gross domestic" or "lower gross national" or lmic or lmics or "third world" or "lami country" or "lami	39,219

countries" or "transitional country" or "transitional economies" or "emerging economy" or "emerging economies" or "emerging nation" or "emerging nations") OR AB ("developing country" or "developing countries" or "developing nation" or "developing nations" or "developing population" or "developing populations" or "developing world" or "less developed country" or "less developed countries" or "less developed nation" or "less developed nations" or "less developed population" or "less developed populations" or "less developed world" or "lesser developed country" or "lesser developed countries" or "lesser developed nation" or "lesser developed nations" or "lesser developed population" or "lesser developed populations" or "lesser developed world" or "under developed country" or "under developed countries" or "under developed nation" or "under developed nations" or "under developed population" or "under developed populations" or "under developed world" or "underdeveloped country" or "underdeveloped countries" or "underdeveloped nation" or "underdeveloped nations" or "underdeveloped population" or "underdeveloped populations" or "underdeveloped world" or "middle income country" or "middle income countries" or "middle income nation" or "middle income nations" or "middle income population" or "middle income populations" or "low income country" or "low income countries" or "low income nation" or "low income nations" or "low income population" or "low income populations" or "lower income country" or "lower income countries" or "lower income nation" or "lower income nations" or "lower income population" or "lower income populations" or "underserved country" or "underserved countries" or "underserved nation" or "underserved nations" or "underserved population" or "underserved populations" or "underserved world" or "under served country" or "under served countries" or "under served nation" or "under served nations" or "under served population" or "under served populations" or "under served world" or "deprived country" or "deprived countries" or "deprived nation" or "deprived nations" or "deprived population" or "deprived populations" or "deprived world" or "poor country" or "poor countries" or "poor nation" or "poor nations" or "poor population" or "poor populations" or "poor world" or "poorer country" or "poorer countries" or "poorer nation" or "poorer nations" or "poorer population" or "poorer populations" or "poorer world" or "developing economy" or "developing economies" or "less developed economy" or "less developed economics" or "lesser developed economy" or "lesser developed economies" or "under developed economy" or "under developed economies" or "underdeveloped economy" or "underdeveloped economies" or "middle income economy" or "middle income economies" or "low income economy" or "low income economies" or "lower income economy" or "lower income economies" or "low gdp" or "low gnp" or "low gross domestic" or "low gross national" or "lower gdp" or "lower gnp" or "lower gross domestic" or "lower gross national" or lmic or lmics or "third world" or "lami country" or "lami countries" or "transitional country" or "transitional economies" or "emerging economy" or "emerging economies" or "emerging nation" or "emerging nations")

S41	TI (mali or malta or micronesia or "federated states of micronesia" or kiribati or "marshall islands" or nauru or "northern mariana islands" or palau or tuvalu or mauritania or mauritius or mexico or moldova or moldovian or mongolia or montenegro or morocco or ifni or mozambique or "portuguese east africa" or myanmar or burma or namibia or nepal or "netherlands antilles" or nicaragua or niger or nigeria or oman or muscat or pakistan or panama or "papua new guinea" or "new guinea" or paraguay or peru or philippines or philipines or phillipines or phillippines or poland or "polish people's republic" or portugal or "portuguese republic" or "puerto rico" or romania or russia or "russian federation" or ussr or "soviet union" or "union of soviet socialist republics" or rwanda or ruanda or samoa or "pacific islands" or polynesia or "samoan islands" or "navigator island" or "navigator islands" or "sao tome and principe" or "saudi arabia" or senegal or serbia or seychelles or "sierra leone" or slovakia or "slovak republic" or slovenia or melanesia or "solomon island" or "solomon islands" or "norfolk island" or "norfolk islands" or somalia or "south africa" or "south sudan" or "sri lanka" or ceylon or "saint kitts and nevis" or "st. kitts and nevis" or "saint lucia" or "st. lucia" or "saint Vincent and the grenadines" or "saint vincent" or "st. vincent" or grenadines or sudan or suriname or surinam or "dutch guiana" or "netherlands guiana" or syria or "syrian arab republic" or tajikistan or tadjikistan or tadzhikistan or tadzhik or tanzania or tanganyika or thailand or siam or "timor leste" or "east timor" or togo or "togolese republic" or tonga or "Trinidad and tobago" or trinidad or tobago or tunisia or turkey or turkmenistan or turkmen or uganda or ukraine or uruguay or uzbekistan or uzbek or vanuatu or "new hebrides" or venezuela or vietnam or "viet nam" or "middle east" or "west bank" or gaza or palestine or yemen or yugoslavia or zambia or zimbabwe or "northern rhodesia" or "global south" or "africa south of the sahara" or "sub-saharan africa" or "subsaharan africa" or "africa, central" or "central africa" or "africa, northern" or "north africa" or "northern africa" or magreb or maghrib or sahara or "africa, southern" or "southern africa" or "africa, eastern" or "east africa" or "eastern africa" or "africa, western" or "west africa" or "western africa" or "west indies" or caribbean or "central america" or "latin america" or "south and central america" or "south america" or "asia, central" or "central asia" or "asia, northern" or "north asia" or "northern asia" or "asia, southeastern" or "southeastern asia" or "south eastern asia" or "southeast asia" or "south east asia" or "asia, western" or "western asia" or "europe, eastern" or "east europe" or "eastern europe") OR AB (mali or malta or micronesia or "federated states of micronesia" or kiribati or "marshall islands" or nauru or "northern mariana islands" or palau or tuvalu or mauritania or mauritius or mexico or moldova or moldovian or mongolia or montenegro or morocco or ifni or mozambique or "portuguese east africa" or myanmar or burma or namibia or nepal or "netherlands antilles" or nicaragua or niger or nigeria or oman or muscat or pakistan or panama or "papua new guinea" or "new guinea" or paraguay or peru or philippines or philipines or phillipines or phillippines or poland or "polish people's republic" or portugal or "portuguese republic" or "puerto rico" or romania or russia or "russian federation" or ussr or "soviet union" or "union of soviet socialist republics" or rwanda or ruanda	148,518

	or samoa or "pacific islands" or polynesia or "samoan islands" or "navigator island" or "navigator islands" or "sao tome and principe" or "saudi arabia" or senegal or serbia or seychelles or "sierra leone" or slovakia or "slovak republic" or slovenia or melanesia or "solomon island" or "solomon islands" or "norfolk island" or "norfolk islands" or somalia or "south africa" or "south sudan" or "sri lanka" or ceylon or "saint kitts and nevis" or "st. kitts and nevis" or "saint lucia" or "st. lucia" or "saint Vincent and the grenadines" or "saint vincent" or "st. vincent" or grenadines or sudan or suriname or surinam or "dutch guiana" or "netherlands guiana" or syria or "syrian arab republic" or tajikistan or tadjikistan or tadzhikistan or tadzhik or tanzania or tanganyika or thailand or siam or "timor leste" or "east timor" or togo or "togolese republic" or tonga or "Trinidad and tobago" or trinidad or tobago or tunisia or turkey or turkmenistan or turkmen or uganda or ukraine or uruguay or uzbekistan or uzbek or vanuatu or "new hebrides" or venezuela or vietnam or "viet nam" or "middle east" or "west bank" or gaza or palestine or yemen or yugoslavia or zambia or zimbabwe or "northern rhodesia" or "global south" or "africa south of the sahara" or "sub-saharan africa" or "subsaharan africa" or "africa, central" or "central africa" or "africa, northern" or "north africa" or "northern africa" or magreb or maghrib or sahara or "africa, southern" or "southern africa" or "africa, eastern" or "east africa" or "eastern africa" or "africa, western" or "west africa" or "western africa" or "west indies" or caribbean or "central america" or "latin america" or "south and central america" or "south america" or "asia, central" or "central asia" or "asia, northern" or "north asia" or "northern asia" or "asia, southeastern" or "southeastern asia" or "south eastern asia" or "southeast asia" or "south east asia" or "asia, western" or "western asia" or "europe, eastern" or "east europe" or "eastern europe")	
S40	TI (afghanistan or albania or algeria or "american samoa" or angola or antigua or barbuda or argentina or armenia or armenian or aruba or azerbaijan or bahrain or bangladesh or barbados or belarus or byelarus or belorussia or byelorussian or belize or "british honduras" or benin or dahomey or bhutan or bolivia or "bosnia herzegovina" or bosnia or herzegovina or botswana or bechuanaland or brazil or brasil or bulgaria or "burkina faso" or "burkina fasso" or "upper volta" or burundi or urundi or "cabo verde" or "cape verde" or cambodia or kampuchea or "khmer republic" or cameroon or cameron or cameroun or "central african republic" or "ubangi shari" or chad or chile or china or colombia or comoros or "comoro islands" or "iles comores" or mayotte or "democratic republic of the congo" or "democratic republic congo" or congo or zaire or "costa rica" or "cote d’ivoire" or "cote d’ ivoire" or "cote divoire" or "cote d ivoire" or "ivory coast" or croatia or cuba or cyprus or "czech republic" or czechoslovakia or djibouti or "french somaliland" or dominica or "dominican republic" or ecuador or egypt or "united arab republic" or "el salvador" or "equatorial guinea" or "spanish guinea" or eritrea or estonia or eswatini or swaziland or ethiopia or fiji or gabon or "gabonese republic" or gambia or "georgia (republic)" or georgian or ghana or "gold coast" or gibraltar or greece or grenada or guam or guatemala or guinea or "guinea bissau" or guyana or "british guiana" or haiti or hispaniola or	230,968

	honduras or hungary or india or indonesia or timor or iran or iraq or "isle of man" or jamaica or jordan or kazakhstan or kazakh or kenya or "democratic people’s republic of korea" or "republic of korea" or "north korea" or "south korea" or korea or kosovo or kyrgyzstan or kirghizia or kirgizstan or "kyrgyz republic" or kirghiz or laos or "lao pdr" or "lao people's democratic republic" or latvia or lebanon or lebanese republic or lesotho or basutoland or liberia or libya or "libyan arab jamahiriya" or lithuania or macau or macao or republic of "north macedonia" or macedonia or madagascar or "malagasy republic" or malawi or nyasaland or malaysia or "malay federation" or "malaya federation" or maldives or "indian ocean islands" or "indian ocean") OR AB (afghanistan or albania or algeria or "american samoa" or angola or antigua or barbuda or argentina or armenia or armenian or aruba or azerbaijan or bahrain or bangladesh or barbados or belarus or byelarus or belorussia or byelorussian or belize or "british honduras" or benin or dahomey or bhutan or bolivia or "bosnia herzegovina" or bosnia or herzegovina or botswana or bechuanaland or brazil or brasil or bulgaria or "burkina faso" or "burkina fasso" or "upper volta" or burundi or urundi or "cabo verde" or "cape verde" or cambodia or kampuchea or "khmer republic" or cameroon or cameron or cameroun or "central african republic" or "ubangi shari" or chad or chile or china or colombia or comoros or "comoro islands" or "iles comores" or mayotte or "democratic republic of the congo" or "democratic republic congo" or congo or zaire or "costa rica" or "cote d’ivoire" or "cote d’ ivoire" or "cote divoire" or "cote d ivoire" or "ivory coast" or croatia or cuba or cyprus or "czech republic" or czechoslovakia or djibouti or "french somaliland" or dominica or "dominican republic" or ecuador or egypt or "united arab republic" or "el salvador" or "equatorial guinea" or "spanish guinea" or eritrea or estonia or eswatini or swaziland or ethiopia or fiji or gabon or "gabonese republic" or gambia or "georgia (republic)" or georgian or ghana or "gold coast" or gibraltar or greece or grenada or guam or guatemala or guinea or "guinea bissau" or guyana or "british guiana" or haiti or hispaniola or honduras or hungary or india or indonesia or timor or iran or iraq or "isle of man" or jamaica or jordan or kazakhstan or kazakh or kenya or "democratic people’s republic of korea" or "republic of korea" or "north korea" or "south korea" or korea or kosovo or kyrgyzstan or kirghizia or kirgizstan or "kyrgyz republic" or kirghiz or laos or "lao pdr" or "lao people's democratic republic" or latvia or lebanon or lebanese republic or lesotho or basutoland or liberia or libya or "libyan arab jamahiriya" or lithuania or macau or macao or republic of "north macedonia" or macedonia or madagascar or "malagasy republic" or malawi or nyasaland or malaysia or "malay federation" or "malaya federation" or maldives or "indian ocean islands" or "indian ocean")	
S39	S1 OR S38	15,244
S38	S16 AND S37	15,243
S37	S17 OR S18 OR S19 OR S20 OR S21 OR S22 OR S23 OR S24 OR S25 OR S26 OR S27 OR S28 OR S29 OR S30 OR S31 OR S32 OR S33 OR S34 OR S35 OR S36	354,620

S36	(MH "Product Surveillance+/SN")	1
S35	(MH "Maternal Health Services+/SN")	1,245
S34	TI pharmacovigilan* or (pharmaco W0 vigilan*)	846
S33	(MH "Product Surveillance+")	2,417
S32	TI "data system" or "data systems" or "information system" or "information systems"	4,466
S31	(MH "Health Information Systems")	3,566
S30	TI (decision* AND support* AND clinical*)	1,865
S29	(MH "Decision Support Systems, Clinical")	6,139
S28	TI surveillance*	15,975
S27	(MH "Population Surveillance+")	11,277
S26	TI survey*	71,609
S25	(MH "Surveys")	157,567
S24	TI registry or registries or eregistr* or "e-registry" or "e-registries"	13,946
S23	TI (preliminary or "pilot project" or "pilot projects") AND data	682
S22	TI "focus group" or "focus groups"	2,957
S21	(MH "Focus Groups")	48,414
S20	TI databas* or "data base" or "data bases" or databank* or "data bank" or "data banks" or dataset* or "data set" or "data sets"	16,019
S19	TI data AND (accumulat* or accura* or assembl* or captur* or collect* or compil* or coordinat* or co-ordinat* or gather* or hub or hubs)	4,425
S18	(MH "Data Curation")	184
S17	(MH "Data Collection")	49,942
S16	S2 OR S3 OR S4 OR S5 OR S6 OR S7 OR S8 OR S9 OR S10 OR S11 OR S12 OR S13 OR S14 OR S15	290,175
S15	TI maternal AND (fetal or fetus or foetal or foetus)	4,004
S14	TI (maternal* or maternity) AND (care or health* or service or services)	9,718
S13	(MH "Maternal Health Services")	11,199
S12	TI (maternal* or maternity) AND (contact* or expos*)	1,028
S11	(MH "Maternal Exposure")	2,588

S10	TI perinatal* or "peri-natal" or "peri-natally" or peripartum or "peri-partum"	12,858
S9	(MH "Perinatal Care")	5,019
S8	TI prenatal* or antenatal* or "ante-natal" or "ante-natally" or antepartum or "ante-partum"	20,877
S7	(MH "Prenatal Care")	19,422
S6	TI pregnan*	80,123
S5	(MH "Pregnancy Trimesters+")	13,521
S4	(MH "Expectant Mothers")	11,382
S3	(MH "Pregnancy Complications+")	106,825
S2	(MH "Pregnancy") OR (MH "Pregnancy, Multiple+") OR (MH "Pregnancy, Unplanned") OR (MH "Pregnancy, Unwanted") OR (MH "Pregnancy, High Risk") OR (MH "Pregnancy, Prolonged")	227,240
S1	TI ("pregnancy exposure database" OR "pregnancy exposure databases" OR "pregnancy exposure data base" OR "pregnancy exposure data bases" OR "pregnancy exposure registry" OR "pregnancy exposure registries") OR AB ("pregnancy exposure database" OR "pregnancy exposure databases" OR "pregnancy exposure data base" OR "pregnancy exposure data bases" OR "pregnancy exposure registry" OR "pregnancy exposure registries")	7

Global Index Medicus

tw:((tw:(pregnan* exposure* database*)) OR (tw:(pregnan* exposure* "data base")) OR (tw:(pregnan* exposure* "data bases")) OR (tw:(pregnan* exposure* databank*)) OR (tw:(pregnan* exposure* "data bank")) OR (tw:(pregnan* exposure* "data banks")) OR (tw:(pregnan* exposure* register)) OR (tw:(pregnan* exposure* registries)) OR (tw:(pregnan* exposure* registry)) OR (tw:(pregnan* exposure* registries))) – *108 records*

tw:((ti:((pregnan* OR prenatal* OR antenatal* OR "ante-natal" OR "ante-natally" OR antepartum OR "ante-partum" OR perinatal* OR "peri-natal" OR "peri-natally" OR peripartum OR "peri-partum" OR maternal*) AND (data OR database* OR databank* OR register OR registers OR registry OR registries OR survey* OR surveillance OR pharmacovigilan* OR "pharmaco-vigilance") AND (safe OR safety OR "side effect" of "side effects" OR "undesirable effect" OR "undesirable effects" OR "treatment emergent" OR tolerability OR toxicity OR adrs OR aefi)))) – *361 records*

tw:(ti:((pregnan* OR prenatal* OR antenatal* OR "ante-natal" OR "ante-natally" OR antepartum OR "ante-partum" OR perinatal* OR "peri-natal" OR "peri-natally" OR peripartum OR "peri-partum" OR maternal*) AND (data OR database* OR databank* OR register OR registers OR registry OR registries OR survey* OR surveillance OR pharmacovigilan* OR "pharmaco-vigilance") AND (MNCH or "maternal health" or "child health" or "infant health" or "birth defect" or "birth defects" or "birth outcome" or

"birth outcomes" or "pregnancy outcome" or "pregnancy outcomes" or "neonatal outcome" or "neonatal outcomes"))) – *50 records*

tw:(ti:((pregnan* OR prenatal* OR antenatal* OR "ante-natal" OR "ante-natally" OR antepartum OR "ante-partum" OR perinatal* OR "peri-natal" OR "peri-natally" OR peripartum OR "peri-partum" OR maternal*) AND (data OR database* OR databank* OR register OR registers OR registry OR registries OR survey* OR surveillance OR pharmacovigilan* OR "pharmaco-vigilance") AND (vaccine or vaccines or vaccination* or immunization* or immunization* or “maternally-acquired”))) – *0 records*

tw:((ti:((pregnan* OR prenatal* OR antenatal* OR "ante-natal" OR "ante-natally" OR antepartum OR "ante-partum" OR perinatal* OR "peri-natal" OR "peri-natally" OR peripartum OR "peri-partum" OR maternal*) AND (data OR database* OR databank* OR register OR registers OR registry OR registries OR survey* OR surveillance OR pharmacovigilan* OR "pharmaco-vigilance") AND (drug OR drugs OR medicine OR medicines OR medication* OR pharmaceutical* OR "pharma-ceutical" OR "pharma-ceuticals" OR vaccin* OR immun* OR antimalarial* OR "anti-malarial" OR "anti-malarials" OR antiviral* OR "anti-viral" OR "anti-virals" OR antiretroviral* OR "anti-retroviral" OR "anti-retrovirals" OR "Anti-HIV" OR "Anti-AIDS")))) – *10 records*

tw:((ti:((pregnan* OR prenatal* OR antenatal* OR "ante-natal" OR "ante-natally" OR antepartum OR "ante-partum" OR perinatal* OR "peri-natal" OR "peri-natally" OR peripartum OR "peri-partum" OR maternal*) AND (data OR database* OR databank* OR register OR registers OR registry OR registries OR survey* OR surveillance OR pharmacovigilan* OR "pharmaco-vigilance") AND (fetal* OR foetal* OR fetus* OR foetus* OR neonat* OR newborn* OR infant OR infants OR infanc* OR child*)))) – *125 records*

tw:((ti:((pregnan* OR prenatal* OR antenatal* OR "ante-natal" OR "ante-natally" OR antepartum OR "ante-partum" OR perinatal* OR "peri-natal" OR "peri-natally" OR peripartum OR "peri-partum" OR maternal*) AND (data OR database* OR databank* OR register OR registers OR registry OR registries OR survey* OR surveillance OR pharmacovigilan* OR "pharmaco-vigilance"))) AND (tw:(drug OR drugs OR medicine OR medicines OR medication* OR pharmaceutical* OR "pharma-ceutical" OR "pharma-ceuticals" OR vaccin* OR immunisation* OR immunization* OR antimalarial* OR "anti-malarial" OR "anti-malarials" OR antiviral* OR "anti-viral" OR "anti-virals" OR antiretroviral* OR "anti-retroviral" OR "anti-retrovirals" OR "Anti-HIV" OR "Anti-AIDS"))) – *71 records*

TOTAL: 725 records

Google Scholar

"pregnant|pregnancy|prenatal"+"registry|registries|surveillance|pharmacovigilance" +

"expose|exposed|exposes|exposure|exposures"+[names of LMICs]
