# Supplementary material for: Pregnancy exposure registries for drugs and vaccines in low-income and middle-income countries: scoping review protocol
Source: BMJ Open. 2023 May 8;13(5):e070543. doi: 10.1136/bmjopen-2022-070543 (PMC10174003; doi:10.1136/bmjopen-2022-070543)
Supplement: Supplementary data [file bmjopen-2022-070543supp002.pdf]

Data Extraction Form

PART 1: STUDY IDENTIFICATION / INCLUSION STATUS

Resource title:

Author(s):

Year of publication:

Resource type:

1. 1 - Peer reviewed journal
2. 2 - Grey literature
3. 3 - Registry
4. 4 - Website
5. 5 - Key informant survey / interview
6. Other

Database resource pulled from:

Final status:

1. 1 - included
2. 2 - excluded
3. 3 - incomplete/unclear

If excluded, explain reasoning:

PART 2: REGISTRY INFORMATION OR OTHER RESOURCE

Name of registry or other resource:

Primary goals/aims:

Funding source:

Years of operation:

Current status

1. Active
2. Inactive
3. On hold

Country(ies) where located:

Country representativeness:

1. Multi-national
2. National
3. State/Provincial
4. District
5. Sub-district
6. Community
7. Health clinic
8. Hospital
9. Other

Drug/vaccine exposure name and type:

Duration of follow up:

Current sample size:

1. <500 participants
2. 500 - 1,999 participants
3. 2,000 - 5,000 participants
4. 5,001 - 10,000 participants
5. 10,001 - 20,000 participants
6. >20,000 participants

Data collection:

1. Administrative databases
2. Medical databases
3. Registries
4. Research study
5. Program implementation
6. Other

Methodology:
Terminology and data system used:

PART 3: CHARACTERISTICS OF INCLUDED POPULATION

Age ranges included:
Target population:

1. Pregnant women
2. Non-pregnant women / women of reproductive age
3. Children
4. Infants (<28 days old)
5. General population
6. Other

Gestational age

1. ☐ First trimester
2. ☐ Second trimester
3. ☐ Third trimester

Underlying medical condition(s):

Maternal outcome(s) recorded:

1. ☐ Thrombosis and/or thrombocytopenia syndrome
2. ☐ Antenatal hospitalization (not including delivery)
3. ☐ Disability
4. ☐ Gestational diabetes
5. ☐ Gestational hypertension
6. ☐ Premature rupture of membranes
7. ☐ Preterm labor
8. ☐ Preeclampsia / Eclampsia
9. ☐ Post-partum hemorrhage
10. ☐ Antenatal hemorrhage
11. ☐ Corporeal infection
12. ☐ Spontaneous abortion / miscarriage / pregnancy loss (example: prior to 20 weeks gestation)
13. ☐ Maternal death (example: within 42 days of termination of pregnancy)
14. ☐ Late maternal death (example: >42 days of termination of pregnancy)
15. ☐ Other

Maternal death cause:

1. ○ Direct cause
2. ○ Indirect cause

Neonatal outcome(s) recorded:

1. ☐ Preterm birth
2. ☐ Small size for gestational age / restricted fetal growth
3. ☐ Still birth (death after 28 weeks of pregnancy but before birth)
4. ☐ Live birth
5. ☐ Congenital anomaly / birth defect
6. ☐ Death
7. ☐ Other

Neonatal death timeframe

1. Early neonatal (0-7 days)
2. Late neonatal (8-28 days)
3. Post neonatal (29 days - 1 year)

Which congenital anomaly does this monitor (if any)?

Infant/child outcome(s) recorded:

1. Infections
2. Respiratory illness
3. Developmental outcomes
4. Other

What is the duration of follow up?

PART 4: KEY FINDINGS

Populate as best you can if information is provided; answer N/A if it is not relevant or N/P if it is not provided.

Strengths of the registry or other resource:

Weaknesses / gaps of registry or other resource:

Challenges of registry or other resource in its specific context:

Does this resource have the possibility to add new interventions?

1. Yes
2. No
3. Maybe
4. Unknown

Why?

Can this resource be combined with other systems?

1. Yes
2. No
3. Maybe
4. Unknown

Why?

Any upcoming changes to the resource:

Additional comments
