# Supplementary material for: Pregnancy exposure registries for drugs and vaccines in low-income and middle-income countries: scoping review protocol
Source: BMJ Open. 2023 May 8;13(5):e070543. doi: 10.1136/bmjopen-2022-070543 (PMC10174003; doi:10.1136/bmjopen-2022-070543)
Supplement: Author's manuscript [file bmjopen-2022-070543.draft_revisions.pdf]

BMJ Open

BMJ Open is committed to open peer review. As part of this commitment we make the peer review history of every article we publish publicly available.

When an article is published we post the peer reviewers' comments and the authors' responses online. We also post the versions of the paper that were used during peer review. These are the versions that the peer review comments apply to.

The versions of the paper that follow are the versions that were submitted during the peer review process. They are not the versions of record or the final published versions. They should not be cited or distributed as the published version of this manuscript.

BMJ Open is an open access journal and the full, final, typeset and author-corrected version of record of the manuscript is available on our site with no access controls, subscription charges or pay-per-view fees (http://bmjopen.bmj.com).

If you have any questions on BMJ Open's open peer review process please email info.bmjopen@bmj.com

BMJ Open

# Pregnancy Exposure Registries in Low- and Middle-Income Countries: Scoping Review Protocol

| | |
|---|---|
| Journal: | *BMJ Open* |
| Manuscript ID | bmjopen-2022-070543 |
| Article Type: | Protocol |
| Date Submitted by the Author: | 26-Nov-2022 |
| Complete List of Authors: | AbuShweimeh, Rahmeh; University of Washington, School of Public Health<br>Knudson, Sophie; PATH, Center for Vaccine Innovation and Access<br>Chaabane, Sonia; World Health Organization, Regulation and Safety<br>Pal, Shanthi Narayan; World Health Organization<br>Skidmore, Becky; 30. Independent Information Specialist<br>Stergachis, Andy; University of Washington School of Public Health, Pharmacy;<br>Bhat, Niranjan ; Center for Vaccine Innovation and Access, PATH, |
| Keywords: | CLINICAL PHARMACOLOGY, Maternal medicine < OBSTETRICS, EPIDEMIOLOGY |
| | |

SCHOLARONE™
Manuscripts

# Pregnancy Exposure Registries in Low- and Middle-Income Countries: Scoping Review Protocol

**Authors:** Rahmeh AbuShweimeh[1], Sophia Knudson[2], Sonia Chaabane[3], Shanthi Narayan Pal[3], Becky Skidmore,[4] Andy Stergachis[1,5], Niranjan Bhat[2]

**Affiliations:**

1. School of Public Health, Hans Rosling Center for Population Health, University of Washington, 3980 15th Ave NE, Seattle, WA 98195
2. PATH, 2201 Westlake Ave, Suite 200, Seattle, WA 98121
3. Regulation and Safety, World Health Organization, Avenue Appia 20, 1211, Geneva, Switzerland
4. Independent Information Specialist, Ottawa, Canada
5. School of Pharmacy, University of Washington, Box 357631, Seattle, WA 98195

**Corresponding Author:**

Dr. Niranjan Bhat; nbhat@path.org

## Abstract

***Background***: Data regarding the safety of drugs and vaccines in pregnant women are typically unavailable before licensure. Pregnancy exposure registries (PERs) are an important source of post-marketing safety information. PERs in low- and middle-income countries (LMICs) are uncommon but can provide valuable safety data from their distinct contexts and will become more relevant as the use of new drugs and vaccines in pregnancy increase worldwide. Strategies to support PERs in LMICs must be based on a better understanding of their current status. We developed a scoping review protocol to assess the landscape of PERs that operate in LMICs and characterize their strengths and challenges.



For peer review only - http://bmjopen.bmj.com/site/about/guidelines.xhtml

For peer review only

***Methods and Analysis*** This protocol follows the Joanna Briggs Institute (JBI) manual for scoping reviews, and the search strategy will be reported using the Preferred Reporting Items for Systematic reviews and Meta-Analyses extension for Scoping Reviews (PRISMA-ScR) Checklist. We will search PubMed, Embase, CINAHL, and WHO's Global Index Medicus, as well as the reference lists of retrieved full-text records, for articles published between 2000 and 2022 that describe PERs or other resources that systematically record exposures to medical products during pregnancy and maternal and infant outcomes in LMICs. Title and abstracts will be screened by two authors and data extracted using a standardized form. We will undertake a grey literature search using Google Scholar and targeted websites. We will distribute an online survey to and conduct semi-structured interviews with selected experts and key informants. Identified PERs will be summarized in tables and analyzed.

***Ethics and dissemination*** Ethical approval is not required for this activity, as it was determined not to involve human-subjects research. Findings will be submitted to an open access peer-reviewed journal and may be presented at conferences, with underlying data and other materials made publicly available.

***Registration*** Open Science Framework (DOI: https://doi.org/10.17605/OSF.IO/FU5AT).



For peer review only - http://bmjopen.bmj.com/site/about/guidelines.xhtml

## Background

Newly introduced vaccines and drugs hold the promise of reducing morbidity and mortality among pregnant women and infants living in low- and middle-income countries (LMICs). However, since pregnant women are actively excluded from most pre-registration clinical trials, safety information for this group is rarely available at the time of a medical product's licensure or approval.[1,2] Consequently, the safety of drugs and vaccines administered during pregnancy must be evaluated throughout their life cycle, including through active surveillance approaches during the post-licensure or post-authorization phase. A commonly used method to systematically assess post-approval safety of drugs and vaccines in pregnant women and their offspring is through the use of a pregnancy exposure registry (PER). A PER is an observational study that systematically collects health information on exposure to medical products such as drugs and vaccines during pregnancy.

PERs, particularly in high-income countries (HICs), are used throughout the post-marketing phase to monitor the safety of drugs and vaccines used during pregnancy.[3,4] Pregnancy exposure registries have been used infrequently in LMICs, where there are unique challenges with respect to knowledge of background rates for obstetric and neonatal outcomes, access to the interventions under evaluation, and the availability of data collection resources and infrastructure. This latter point underscores the limitations in capturing information on the use of vaccines and other medicines in pregnancy, as well as information on the occurrence of obstetric/perinatal complications and other outcomes, and the capacity to link these data sources together[2]. Examples of pregnancy exposure registries in LMICs include those established for drugs or vaccines of particular relevance for their populations, such as those used to treat malaria



For peer review only - http://bmjopen.bmj.com/site/about/guidelines.xhtml

and HIV and prevent COVID-19.[5,6,7] Maternal immunization (MI) has been shown to be an effective method to protect women during pregnancy and their newborns, and use in LMICs is growing.[8] MI is anticipated to increase further in the coming years as a result of the continued adoption of COVID-19 vaccines and the introduction of promising new MI-specific vaccines, such as those for respiratory syncytial virus (RSV) and group B *Streptococcus* (GBS). Pre-licensure safety data on these vaccines will largely come from HIC settings, highlighting the importance of monitoring their post-marketing safety in LMICs.[9,10]

Most research assessing the global status of drug and vaccine safety monitoring in pregnancy has focused on HICs.[11] However, one recent study assessed existing maternal, newborn, and child health (MNCH) data collection systems in LMICs that could be used to monitor drug or vaccine safety and another study assessed the feasibility of use of Global Alignment of Immunization Safety Assessment in pregnancy (GAIA) case definitions for neonatal outcomes and maternal vaccination in LMICs.[12,13] In contrast to these broader surveillance systems, PERs focus on the active data collection specifically related to medical product exposures and safety outcomes, and may be conducted by private as well as public agencies.[14] An improved understanding of PERs in LMICs can better inform how future public health efforts in their maternal populations, such as new vaccine introductions and treatment programs, can be supported. To address this need, we aim to conduct a scoping review to identify and describe PERs and other similar resources that operate in LMICs. Here, we report the methodology for our planned scoping review.


For peer review only - http://bmjopen.bmj.com/site/about/guidelines.xhtml

## Study objective

This scoping review aims to identify resources that systematically record exposures to medical products during pregnancy and maternal and infant outcomes in LMICs. Pregnancy exposure registries, databases and other routinely collected health data of relevance will be targeted. This scoping review will consist of a systematic search of the scientific and grey literature, supplemented by an online survey and interviews with selected key informants, as needed.

## Study Design

This protocol follows the Joanna Briggs Institute (JBI) manual for scoping reviews, and the search strategy will be reported using the Preferred Reporting Items for Systematic reviews and Meta-Analyses extension for Scoping Reviews (PRISMA-ScR) Checklist.[15,16,17] This protocol has been registered with the Open Science Framework (DOI: https://doi.org/10.17605/OSF.IO/FU5AT).[18]

Following multiple consultations with key stakeholders, three primary review questions were selected:

1. What pregnancy exposure cohorts, databases and registries exist in LMICs?
2. What types of data, processes, and tools are included in these databases and registries?
3. What are the strengths and weaknesses of the identified databases and registries?

In addition, two secondary questions were identified:

1. Can the pregnancy exposure registries that have been identified in LMICs be used or adapted to monitor additional new vaccines or drugs that may be introduced for pregnant women?



For peer review only - http://bmjopen.bmj.com/site/about/guidelines.xhtml

2. What is the potential for data harmonization and/or combining of data across databases and registries?

Based on these objectives, the following eligibility criteria for publication selection were developed:

*Inclusion criteria*

1. Publications and documents published or produced from January 2000 to the present, to ensure that identified registries possess features that are more relevant to current scientific and technological conditions; online sources will be accessed.
2. Populations studied are located entirely or at least partially in low- and middle-income countries.[19] (World Bank definition, as of 2016).
3. Reference to prospective and retrospective electronic or combined paper-electronic data collection systems including demographic national registers in LMICs.
4. Reference to prospective and retrospective cohort studies, with no restrictions regarding age range other than women of childbearing age (i.e., 15-49 years of age) or underlying conditions other than pregnancy.
5. Reference to systems that collect data on exposure to one or more drugs or vaccines during pregnancy.
6. Reference to systems that collect data on pregnancy outcomes, including delivery, post-partum, and neonatal (may include an extended time frame to include birth defects detected later).

*Exclusion criteria*

1. Editorials, opinion pieces, promotional literature
2. Guidelines or guidance documents.



For peer review only - http://bmjopen.bmj.com/site/about/guidelines.xhtml

3. Reference to non-allopathic (e.g., traditional, homeopathic, or naturopathic) interventions

***Search strategy and information sources***

Using an iterative process and in consultation with the review team, an information specialist will develop a strategy in PubMed incorporating controlled vocabulary/Medical Subject Headings (e.g., “Pregnancy”, “Datasets as Topic”, “Product Surveillance, Postmarketing”) and free text (e.g., prenatal, registries, pharmacovigilance). We will apply a LMIC filter to focus results to the geographic regions of interest. Another information specialist will peer review the strategy using the PRESS Checklist[6]. Any necessary edits will be made before finalizing in PubMed and subsequently translating the search to Embase, CINAHL, and WHO’s Global Index Medicus. We will also search the reference lists of potentially relevant records and articles to ensure that our search results are as comprehensive as possible.

Additionally, we will undertake a grey literature search, including Google Scholar search and relevant websites, such as industry and professional organizations, associations and alliances (e.g., Developing Countries Vaccine Manufactures Network (DCVMN), Society for Maternal-Fetal Medicine, American College of Obstetricians and Gynecologists, International Society for Pharmacoepidemiology, International Society of Pharmacovigilance); selected Ministries of Health (including regulatory agencies and pharmacovigilance centers) in LMICs; and selected HIC organizations, including US FDA, EMA, NIH, World Health Organization, Maternal and Child Survival Programs,  Measure Evaluation, UK Teratology Information Service, UK Obstetric Surveillance System, and selected academic and other non-governmental groups. The final search strategy is provided in the appendix.



For peer review only - http://bmjopen.bmj.com/site/about/guidelines.xhtml

## Study selection

Records retrieved by the search strategy will be downloaded to EndNote Version 9.3.3 (Clarivate) for de-duplication and then uploaded to review management software (Covidence) to enable independent screening and track disagreements and consensuses among reviewers. Each title and abstract will be screened by two independent reviewer authors to determine eligibility. Each item will be categorized into one of three categories (Yes, Maybe, No), and disagreements between reviewers will be resolved by a third reviewer. After title and abstract screening, a second round of screening will be conducted for full-text review by two reviewers and a decision will be made for data extraction. An adapted version of the PRISMA flow diagram[7] will be constructed to summarize the number of records screened, assessed for eligibility, and included in the review, with reasons for exclusions at each stage.

### *Data extraction*

A form for data extraction will be used to extract key information regarding the registries from the selected full-text articles and screened grey literature. The form will be pilot-tested and refined during the full-text screening stage in order to capture information more efficiently. Additional categories that may emerge during data extraction will be added accordingly. Key data elements to be collected from the included articles include the following.:

a. Author(s)
b. Year of publication
c. Country(ies) where the registry is located
d. Name and aims/purpose of the registry



For peer review only - http://bmjopen.bmj.com/site/about/guidelines.xhtml

e. Years of registry operation

f. Characteristics of the included population (specifically, the characteristics or eligibility criteria used to be included in the registry)

g. Country representativeness (local/national/regional)

h. Funding source for the registry

i. Current sample size (proportion in LMIC)

j. Methodology/methods, including database type

k. Terminology and data system used (e.g., MedDRA, ICD, Brighton Collaboration)

l. Intervention/Exposure type (e.g., drugs, vaccines,), comparator and details of these, including trimester of exposure

m. Maternal, perinatal, and neonatal outcomes including, but not limited to spontaneous abortions, stillbirths, congenital anomalies and details of these. In addition, documenting whether GAIA definitions or other standardized classifications are used.

n. Duration of follow-up

o. Key findings that relate to the scoping review questions, including strengths, weaknesses, ability to add new interventions, and ability to combine data with other systems

p. Demographic factors, socioeconomic factors, and lifestyle factors

Data will be entered into a database that will allow searching and categorization according to selected characteristics.



For peer review only - http://bmjopen.bmj.com/site/about/guidelines.xhtml

## Informant Survey and Interviews

In addition to searching bibliographic databases and grey literature, an online survey will be sent to experts and key informants to identify additional registries that may not have been captured or to provide additional detail on those already identified. A subset of survey respondents will be identified for semi-structured interviews if additional information is needed about the registries. Interviewees will be asked to discuss the strengths and weaknesses of the registry, relevant contextual factors, and the usability of these resources in their particular settings. All responses will be recorded in an electronic database for analysis.

## Data summary and synthesis of results

Identified PERs will be summarized in tables organized according to relevant characteristics, including geography, methodology, types of interventions included, outcomes captured, and citations. The selected PERs will be further grouped and evaluated based on the primary and secondary scoping review questions (strengths, weaknesses, ability to add new interventions, and ability to combine data with other systems) and other methods of appraisal, and the quality of the existing registries may be discussed as part of the findings. Geographic coverage or other spatial characteristics may be presented using maps.

## Consultation

As part of this activity, a technical working group was established to provide assistance and guidance throughout the course of the review. Members of this group are experts in multiple disciplines, including pharmacovigilance, perinatology, and pediatrics, particularly as practiced in low-resource settings. In addition to this group, the protocol and results of this scoping review



For peer review only - http://bmjopen.bmj.com/site/about/guidelines.xhtml

will be reviewed by an Expert Steering Committee on Safety Surveillance in Pregnancy in LMICs, established by WHO. Feedback from both groups will be incorporated to produce a final document.

## Dissemination and ethics

Ethical approval is not required for this activity, though it has been reviewed by PATH's Research Determination Committee and deemed not research nor human-subjects research. Results of this landscape analysis will be submitted to an open-access peer-reviewed journal for publication and may be presented at conferences, while underlying data and other materials will be made publicly available.

## Funding

This work was supported by a grant from the Bill & Melinda Gates Foundation [grant number INV-037810]. The funders had no role in the design, analysis, or writing of this manuscript.

## Acknowledgments

The authors would like to thank Kaitryn Campbell, MLIS, MSc for peer review of the PubMed search strategy. We also thank the members of our Technical Working Group: Pierre Buekens, Ushma Mehta, Deshayne Fell, Smaragda Lamprianou, and Kate Fay, Ajoke ter Meulen, Nancy Salt, and Jessica Fleming. The authors alone are responsible for the views expressed in this article and they do not necessarily represent the views, decisions or policies of the institutions with which they are affiliated.

## Appendices



For peer review only - http://bmjopen.bmj.com/site/about/guidelines.xhtml

Search Strategy by Database:

**PubMed**

| Set # | Query | Results |
|---|---|---|
| 132 | #130 NOT #131 | 2,016 |
| 131 | address[Publication Type] OR autobiography[Publication Type] OR bibliography[Publication Type] OR biography[Publication Type] OR comment[Publication Type] OR dictionary[Publication Type] OR directory[Publication Type] OR editorial[Publication Type] OR "expression of concern"[Publication Type] OR festschrift[Publication Type] OR historical article[Publication Type] OR interactive tutorial[Publication Type] OR lecture[Publication Type] OR news[Publication Type] OR newspaper article[Publication Type] OR portrait[Publication Type] OR video-audio media[Publication Type] OR webcast[Publication Type] | 2,050,653 |
| 130 | #128 NOT #129 | 2,048 |
| 129 | Animals[mesh] NOT Humans[mesh] | 5,016,862 |
| 128 | #126 AND #127 | 2,051 |
| 127 | ("2000"[Date - Publication] : "3000"[Date - Publication]) | 20,721,844 |
| 126 | #61 AND #125 | 2,597 |
| 125 | #62 OR #63 OR #64 OR #65 OR #66 OR #67 OR #68 OR #69 OR #70 OR #71 OR #72 OR #73 OR #74 OR #75 OR #76 OR #77 OR #78 OR #79 OR #80 OR #81 OR #82 OR #83 OR #84 OR #85 OR #86 OR #87 OR #88 OR #89 OR #90 OR #91 OR #92 OR #93 OR #94 OR #95 OR #96 OR #97 OR #98 OR #99 OR #100 OR #101 OR #102 OR #103 OR #104 OR #105 OR #106 OR #107 OR #108 OR #109 OR #110 OR #111 OR #112 OR #113 OR #114 OR #115 OR #116 OR #117 OR #118 OR #119 OR #120 OR #121 OR #122 OR #123 OR #124 | 7,704,889 |
| 124 | MNCH[Other Term] AND (maternal*[Other Term] OR newborn*[Other Term] OR child*[Other Term]) | 11 |
| 123 | MNCH[Title] AND (maternal*[Title] OR newborn*[Title] OR child*[Title]) | 22 |
| 122 | "Maternal Health"[mesh] | 2,142 |
| 121 | "Child Health"[mesh] | 4,447 |
| 120 | "Infant Health"[mesh] | 1,175 |
| 119 | (birth[Other Term] OR births[Other Term]) AND defect*[Other Term] | 1,029 |
| 118 | (birth[Title] OR births[Title]) AND defect*[Title] | 2,671 |



For peer review only - http://bmjopen.bmj.com/site/about/guidelines.xhtml

| | | |
|---|---|---|
| 117 | (birth[Other Term] OR births[Other Term] OR matern*[Other Term] OR neonat*[Other Term] OR neo-nat*[Other Term] OR perinatal*[Other Term] OR peri-natal*[Other Term] OR peripartum[Other Term] OR "peri-partum"[Other Term] OR postnatal*[Other Term] OR post-natal*[Other Term] OR postpartum[Other Term] OR "post-partum"[Other Term] OR post-birth*[Other Term] OR pregnanc*[Other Term]) AND outcome*[Other Term] | 9,426 |
| 116 | (birth[Title] OR births[Title] OR matern*[Title] OR neonat*[Title] OR neo-nat*[Title] OR perinatal*[Title] OR peri-natal*[Title] OR peripartum[Title] OR "peri-partum"[Title] OR postnatal*[Title] OR post-natal*[Title] OR postpartum[Title] OR "post-partum"[Title] OR post-birth*[Title] OR pregnanc*[Title]) AND outcome*[Title] | 31,066 |
| 115 | Pregnancy Outcome[mesh] | 82,242 |
| 114 | (fetal[OT] OR foetal[OT] OR fetus*[OT] OR foetus*[OT] OR prenatal*[OT] OR pre-natal*[OT]) AND expos*[OT] | 2,033 |
| 113 | (fetal[Title] OR foetal[Title] OR fetus*[Title] OR foetus*[Title] OR prenatal*[Title] OR pre-natal*[Title]) AND expos*[Title] | 10,858 |
| 112 | "Prenatal Exposure Delayed Effects"[mesh] | 33,016 |
| 111 | (fetal[Other Term] OR foetal[Other Term] OR fetus*[Other Term] OR foetus*[Other Term]) AND develop*[Other Term] | 2,531 |
| 110 | (fetal[Title] OR foetal[Title] OR fetus*[Title] OR foetus*[Title]) AND develop*[Title] | 8,734 |
| 109 | "Fetal Development"[mesh:noexp] | 9,246 |
| 108 | antiretroviral*[Other Term] OR anti-retroviral*[Other Term] OR "anti-HIV"[Other Term] OR "anti-AIDS"[Other Term] OR "AIDS drug"[Other Term] OR "AIDS drugs"[Other Term] | 7,307 |
| 107 | (antiretroviral*[Title] OR anti-retroviral*[Title] OR "anti-HIV"[Title] OR "anti-AIDS"[Title] OR "AIDS drug"[Title] OR "AIDS drugs"[Title]) | 29,826 |
| 106 | "Anti-Retroviral Agents"[mesh] | 69,532 |
| 105 | "Zika Virus Infection/prevention and control"[MeSH] | 1,096 |
| 104 | "Streptococcal Infections/prevention and control"[MeSH:noexp] | 2,944 |
| 103 | "Respiratory Syncytial Virus Infections/prevention and control"[MeSH Terms] | 1,832 |
| 102 | "Hepatitis E/prevention and control"[MeSH Terms] | 346 |
| 101 | "Cytomegalovirus Infections/prevention and control"[MeSH Terms] | 3,130 |
| 100 | antimalarial*[Other Term] OR anti-malarial*[Other Term] | 2,577 |
| 99 | antimalarial*[Title] OR anti-malarial*[Title] | 8,562 |
| 98 | Antimalarials[mesh] | 28,226 |

For peer review only

| | | |
|---|---|---|
| 97 | "Malaria/prevention and control"[MeSH Terms] | 17,528 |
| 96 | (maternal[Other Term] OR pregnan*[Other Term]) AND (immunisation*[Other Term] OR immunization*[Other Term]) | 754 |
| 95 | (maternal[Title] OR pregnan*[Title]) AND (immunisation*[Title] OR immunization*[Title]) | 1,153 |
| 94 | (antibod*[Other Term] OR anti-bod*[Other Term]) AND transfer*[Other Term] | 338 |
| 93 | (antibod*[Title] OR anti-bod*[Title]) AND transfer*[Title] | 1,620 |
| 92 | immunit*[Other Term] AND maternally-acqui*[Other Term] | 8 |
| 91 | immunit*[Title] AND maternally-acqui*[Title] | 8 |
| 90 | immunit*[Other Term] AND transfer*[Other Term] | 161 |
| 89 | immunit*[Title] AND transfer*[Title] | 882 |
| 88 | "Immunity, Maternally-Acquired"[mesh] | 5,842 |
| 87 | "Immunization Programs"[mesh:noexp] | 12,435 |
| 86 | Immunization[mesh:noexp] | 144,668 |
| 85 | vaccin*[ti] OR vaccin*[ot] | 220,223 |
| 84 | "Viral Vaccines"[mesh] | 132,729 |
| 83 | "Vaccines, Live, Unattenuated"[mesh] | 82 |
| 82 | "Vaccines, Synthetic"[mesh] | 31,589 |
| 81 | "Vaccines, Inactivated"[mesh:noexp] | 6,082 |
| 80 | "Vaccines, Combined"[mesh] | 10,562 |
| 79 | "Vaccines, Attenuated"[mesh] | 12,810 |
| 78 | "Protozoan Vaccines"[mesh] | 6,894 |
| 77 | "Bacterial Vaccines"[mesh] | 72,365 |
| 76 | Vaccines[mesh:noexp] | 25,746 |
| 75 | Vaccination[mesh] | 100,718 |
| 74 | GAIA[Text Word] AND (alignment[Text Word] OR immunisation[Text Word] OR immunization[Text Word] OR safety[Text Word] OR pregnan*[Text Word]) | 35 |
| 73 | "Global Alignment of Immunization Safety Assessment in Pregnancy"[tw] - Schema: all | 0 |
| 72 | AEFI[Other Term] AND adverse[Other Term] | 68 |
| 71 | AEFI[Title] AND adverse[Title] | 35 |
| 70 | adverse effect[Other Term] OR adverse effects[Other Term] OR adverse reaction[Other Term] OR adverse reactions[Other Term] OR adverse event[Other Term] OR adverse events[Other Term] OR adverse outcome[Other Term] OR adverse outcomes[Other Term] | 17,729 |
| 69 | adverse effect[Title] OR adverse effects[Title] OR adverse reaction[Title] OR adverse reactions[Title] OR adverse event[Title] OR adverse events[Title] OR adverse outcome[Title] OR adverse outcomes[Title] | 31,848 |


For peer review only - http://bmjopen.bmj.com/site/about/guidelines.xhtml

| | | |
|---|---|---|
| 68 | safe[Other Term] OR safety[Other Term] OR side effect[Other Term] OR side effects[Other Term] OR undesirable effect[Other Term] OR undesirable effects[Other Term] OR treatment emergent[Other Term] OR tolerability[Other Term] OR toxicity[Other Term] OR adrs[Other Term] | 98,171 |
| 67 | safe[Title] OR safety[Title] OR side effect[Title] OR side effects[Title] OR undesirable effect[Title] OR undesirable effects[Title] OR treatment emergent[Title] OR tolerability[Title] OR toxicity[Title] OR adrs[Title] | 296,710 |
| 66 | ("adverse effects"[MeSH Subheading]) OR (Complications[MeSH Subheading]) OR ("drug effects"[MeSH Subheading]) | 6,963,724 |
| 65 | (drug[Title] OR drugs[Title] OR medicine[Title] OR medicines[Title] OR medication*[Title] OR pharmaceutical*[Title] OR pharma-ceutical*[Title]) AND expos*[Title] | 4,387 |
| 64 | "Drug-Related Side Effects and Adverse Reactions"[MeSH Terms] | 127,437 |
| 63 | (global[Title] OR international*[Title] OR world*[Title]) AND health*[Title] | 27,778 |
| 62 | Global Health[mesh] | 53,845 |
| 61 | #56 AND #60 | 8,450 |
| 60 | #57 OR #58 OR #59 | 1,104,126 |
| 59 | "high burden country"[Other Term] OR "high burden countries"[Other Term] OR "high-burden country"[Other Term] OR "high-burden countries"[Other Term] OR "countdown country"[Other Term] OR "countdown countries"[Other Term] | 10 |
| 58 | "high burden country"[Title/Abstract] OR "high burden countries"[Title/Abstract] OR "high-burden country"[Title/Abstract] OR "high-burden countries"[Title/Abstract] OR "countdown country"[Title/Abstract] OR "countdown countries"[Title/Abstract] | 633 |



For peer review only - http://bmjopen.bmj.com/site/about/guidelines.xhtml

| | | |
|---|---|---|
| 57 | afghanistan[Text Word] OR albania[Text Word] OR algeria[Text Word] OR "american samoa"[Text Word] OR angola[Text Word] OR antigua[Text Word] OR barbuda[Text Word] OR argentina[Text Word] OR armenia[Text Word] OR armenian[Text Word] OR aruba[Text Word] OR azerbaijan[Text Word] OR bahrain[Text Word] OR bangladesh[Text Word] OR barbados[Text Word] OR belarus[Text Word] OR byelarus[Text Word] OR belorussia[Text Word] OR byelorussian[Text Word] OR belize[Text Word] OR "british honduras"[Text Word] OR benin[Text Word] OR dahomey[Text Word] OR bhutan[Text Word] OR bolivia[Text Word] OR "bosnia herzegovina"[Text Word] OR bosnia[Text Word] OR herzegovina[Text Word] OR botswana[Text Word] OR bechuanaland[Text Word] OR brazil[Text Word] OR brasil[Text Word] OR bulgaria[Text Word] OR "burkina faso"[Text Word] OR "burkina fasso"[Text Word] OR "upper volta"[Text Word] OR burundi[Text Word] OR urundi[Text Word] OR "cabo verde"[Text Word] OR "cape verde"[Text Word] OR cambodia[Text Word] OR kampuchea[Text Word] OR "khmer republic"[Text Word] OR cameroon[Text Word] OR cameron[Text Word] OR cameroun[Text Word] OR "central african republic"[Text Word] OR "ubangi shari"[Text Word] OR chad[Text Word] OR chile[Text Word] OR china[Text Word] OR colombia[Text Word] OR comoros[Text Word] OR "comoro islands"[Text Word] OR "iles comores"[Text Word] OR mayotte[Text Word] OR "democratic republic of the congo"[Text Word] OR "democratic republic congo"[Text Word] OR congo[Text Word] OR zaire[Text Word] OR "costa rica"[Text Word] OR "cote d'ivoire"[Text Word] OR "cote d' ivoire"[Text Word] OR "cote divoire"[Text Word] OR "cote d ivoire"[Text Word] OR "ivory coast"[Text Word] OR croatia[Text Word] OR cuba[Text Word] OR cyprus[Text Word] OR "czech republic"[Text Word] OR czechoslovakia[Text Word] OR djibouti[Text Word] OR "french somaliland"[Text Word] OR dominica[Text Word] OR "dominican republic"[Text Word] OR ecuador[Text Word] OR egypt[Text Word] OR "united arab republic"[Text Word] OR "el salvador"[Text Word] OR "equatorial guinea"[Text Word] OR "spanish guinea"[Text Word] OR eritrea[Text Word] OR estonia[Text Word] OR eswatini[Text Word] OR swaziland[Text Word] OR ethiopia[Text Word] OR fiji[Text Word] OR gabon[Text Word] OR "gabonese republic"[Text Word] OR gambia[Text Word] OR "georgia (republic)"[Text Word] OR georgian[Text Word] OR ghana[Text Word] OR "gold coast"[Text Word] OR gibraltar[Text Word] OR greece[Text Word] OR grenada[Text Word] OR guam[Text Word] OR guatemala[Text Word] OR guinea[Text Word] OR "guinea bissau"[Text Word] OR guyana[Text Word] OR "british guiana"[Text Word] OR haiti[Text Word] OR hispaniola[Text Word] OR honduras[Text Word] OR hungary[Text Word] OR india[Text Word] OR indonesia[Text Word] OR timor[Text Word] OR iran[Text Word] OR iraq[Text Word] OR "isle | 1,103,822 |



For peer review only - http://bmjopen.bmj.com/site/about/guidelines.xhtml

of man"[Text Word] OR jamaica[Text Word] OR jordan[Text Word] OR kazakhstan[Text Word] OR kazakh[Text Word] OR kenya[Text Word] OR "democratic people’s republic of korea"[Text Word] OR "republic of korea"[Text Word] OR "north korea"[Text Word] OR "south korea"[Text Word] OR korea[Text Word] OR kosovo[Text Word] OR kyrgyzstan[Text Word] OR kirghizia[Text Word] OR kirgizstan[Text Word] OR "kyrgyz republic"[Text Word] OR kirghiz[Text Word] OR laos[Text Word] OR "lao pdr"[Text Word] OR "lao people's democratic republic"[Text Word] OR latvia[Text Word] OR lebanon[Text Word] OR lebanese republic[Text Word] OR lesotho[Text Word] OR basutoland[Text Word] OR liberia[Text Word] OR libya[Text Word] OR "libyan arab jamahiriya"[Text Word] OR lithuania[Text Word] OR macau[Text Word] OR macao[Text Word] OR republic of "north macedonia"[Text Word] OR macedonia[Text Word] OR madagascar[Text Word] OR "malagasy republic"[Text Word] OR malawi[Text Word] OR nyasaland[Text Word] OR malaysia[Text Word] OR "malay federation"[Text Word] OR "malaya federation"[Text Word] OR maldives[Text Word] OR "indian ocean islands"[Text Word] OR "indian ocean"[Text Word] OR mali[Text Word] OR malta[Text Word] OR micronesia[Text Word] OR "federated states of micronesia"[Text Word] OR kiribati[Text Word] OR "marshall islands"[Text Word] OR nauru[Text Word] OR "northern mariana islands"[Text Word] OR palau[Text Word] OR tuvalu[Text Word] OR mauritania[Text Word] OR mauritius[Text Word] OR mexico[Text Word] OR moldova[Text Word] OR moldovian[Text Word] OR mongolia[Text Word] OR montenegro[Text Word] OR morocco[Text Word] OR ifni[Text Word] OR mozambique[Text Word] OR "portuguese east africa"[Text Word] OR myanmar[Text Word] OR burma[Text Word] OR namibia[Text Word] OR nepal[Text Word] OR "netherlands antilles"[Text Word] OR nicaragua[Text Word] OR niger[Text Word] OR nigeria[Text Word] OR oman[Text Word] OR muscat[Text Word] OR pakistan[Text Word] OR panama[Text Word] OR "papua new guinea"[Text Word] OR "new guinea"[Text Word] OR paraguay[Text Word] OR peru[Text Word] OR philippines[Text Word] OR philipines[Text Word] OR phillipines[Text Word] OR phillippines[Text Word] OR poland[Text Word] OR "polish people's republic"[Text Word] OR portugal[Text Word] OR "portuguese republic"[Text Word] OR "puerto rico"[Text Word] OR romania[Text Word] OR russia[Text Word] OR "russian federation"[Text Word] OR ussr[Text Word] OR "soviet union"[Text Word] OR "union of soviet socialist republics"[Text Word] OR rwanda[Text Word] OR ruanda[Text Word] OR samoa[Text Word] OR "pacific islands"[Text Word] OR polynesia[Text Word] OR "samoan islands"[Text Word] OR "navigator island"[Text Word] OR "navigator islands"[Text Word] OR "sao tome and principe"[Text Word] OR "saudi arabia"[Text Word] OR senegal[Text Word] OR serbia[Text Word] OR



For peer review only - http://bmjopen.bmj.com/site/about/guidelines.xhtml

seychelles[Text Word] OR "sierra leone"[Text Word] OR slovakia[Text Word] OR "slovak republic"[Text Word] OR slovenia[Text Word] OR melanesia[Text Word] OR "solomon island"[Text Word] OR "solomon islands"[Text Word] OR "norfolk island"[Text Word] OR "norfolk islands"[Text Word] OR somalia[Text Word] OR "south africa"[Text Word] OR "south sudan"[Text Word] OR "sri lanka"[Text Word] OR ceylon[Text Word] OR "saint kitts and nevis"[Text Word] OR "st. kitts and nevis"[Text Word] OR "saint lucia"[Text Word] OR "st. lucia"[Text Word] OR "saint Vincent and the grenadines"[Text Word] OR "saint vincent"[Text Word] OR "st. vincent"[Text Word] OR grenadines[Text Word] OR sudan[Text Word] OR suriname[Text Word] OR surinam[Text Word] OR "dutch guiana"[Text Word] OR "netherlands guiana"[Text Word] OR syria[Text Word] OR "syrian arab republic"[Text Word] OR tajikistan[Text Word] OR tadjikistan[Text Word] OR tadzhikistan[Text Word] OR tadzhik[Text Word] OR tanzania[Text Word] OR tanganyika[Text Word] OR thailand[Text Word] OR siam[Text Word] OR "timor leste"[Text Word] OR "east timor"[Text Word] OR togo[Text Word] OR "togolese republic"[Text Word] OR tonga[Text Word] OR "Trinidad and tobago"[Text Word] OR trinidad[Text Word] OR tobago[Text Word] OR tunisia[Text Word] OR turkey[Text Word] OR turkmenistan[Text Word] OR turkmen[Text Word] OR uganda[Text Word] OR ukraine[Text Word] OR uruguay[Text Word] OR uzbekistan[Text Word] OR uzbek[Text Word] OR vanuatu[Text Word] OR "new hebrides"[Text Word] OR venezuela[Text Word] OR vietnam[Text Word] OR "viet nam"[Text Word] OR "middle east"[Text Word] OR "west bank"[Text Word] OR gaza[Text Word] OR palestine[Text Word] OR yemen[Text Word] OR yugoslavia[Text Word] OR zambia[Text Word] OR zimbabwe[Text Word] OR "northern rhodesia"[Text Word] OR "global south"[Text Word] OR "africa south of the sahara"[Text Word] OR "sub-saharan africa"[Text Word] OR "subsaharan africa"[Text Word] OR "africa, central"[Text Word] OR "central africa"[Text Word] OR "africa, northern"[Text Word] OR "north africa"[Text Word] OR "northern africa"[Text Word] OR magreb[Text Word] OR maghrib[Text Word] OR sahara[Text Word] OR "africa, southern"[Text Word] OR "southern africa"[Text Word] OR "africa, eastern"[Text Word] OR "east africa"[Text Word] OR "eastern africa"[Text Word] OR "africa, western"[Text Word] OR "west africa"[Text Word] OR "western africa"[Text Word] OR "west indies"[Text Word] OR caribbean[Text Word] OR "central america"[Text Word] OR "latin america"[Text Word] OR "south and central america"[Text Word] OR "south america"[Text Word] OR "asia, central"[Text Word] OR "central asia"[Text Word] OR "asia, northern"[Text Word] OR "north asia"[Text Word] OR "northern asia"[Text Word] OR "asia, southeastern"[Text Word] OR "southeastern asia"[Text Word] OR



For peer review only - http://bmjopen.bmj.com/site/about/guidelines.xhtml

"south eastern asia"[Text Word] OR "southeast asia"[Text Word] OR "south east asia"[Text Word] OR "asia, western"[Text Word] OR "western asia"[Text Word] OR "europe, eastern"[Text Word] OR "east europe"[Text Word] OR "eastern europe"[Text Word] OR "developing country"[Text Word] OR "developing countries"[Text Word] OR "developing nation"[Text Word] OR "developing nations"[Text Word] OR "developing population"[Text Word] OR "developing populations"[Text Word] OR "developing world"[Text Word] OR "less developed country"[Text Word] OR "less developed countries"[Text Word] OR "less developed nation"[Text Word] OR "less developed nations"[Text Word] OR "less developed population"[Text Word] OR "less developed populations"[Text Word] OR "less developed world"[Text Word] OR "lesser developed country"[Text Word] OR "lesser developed countries"[Text Word] OR "lesser developed nation"[Text Word] OR "lesser developed nations"[Text Word] OR "lesser developed population"[Text Word] OR "lesser developed populations"[Text Word] OR "lesser developed world"[Text Word] OR "under developed country"[Text Word] OR "under developed countries"[Text Word] OR "under developed nation"[Text Word] OR "under developed nations"[Text Word] OR "under developed population"[Text Word] OR "under developed populations"[Text Word] OR "under developed world"[Text Word] OR "underdeveloped country"[Text Word] OR "underdeveloped countries"[Text Word] OR "underdeveloped nation"[Text Word] OR "underdeveloped nations"[Text Word] OR "underdeveloped population"[Text Word] OR "underdeveloped populations"[Text Word] OR "underdeveloped world"[Text Word] OR "middle income country"[Text Word] OR "middle income countries"[Text Word] OR "middle income nation"[Text Word] OR "middle income nations"[Text Word] OR "middle income population"[Text Word] OR "middle income populations"[Text Word] OR "low income country"[Text Word] OR "low income countries"[Text Word] OR "low income nation"[Text Word] OR "low income nations"[Text Word] OR "low income population"[Text Word] OR "low income populations"[Text Word] OR "lower income country"[Text Word] OR "lower income countries"[Text Word] OR "lower income nation"[Text Word] OR "lower income nations"[Text Word] OR "lower income population"[Text Word] OR "lower income populations"[Text Word] OR "underserved country"[Text Word] OR "underserved countries"[Text Word] OR "underserved nation"[Text Word] OR "underserved nations"[Text Word] OR "underserved population"[Text Word] OR "underserved populations"[Text Word] OR "underserved world"[Text Word] OR "under served country"[Text Word] OR "under served countries"[Text Word] OR "under served nation"[Text Word] OR "under served nations"[Text Word] OR "under served population"[Text Word] OR "under served populations"[Text



For peer review only - http://bmjopen.bmj.com/site/about/guidelines.xhtml

| | | |
|---|---|---|
| | Word] OR "under served world"[Text Word] OR "deprived country"[Text Word] OR "deprived countries"[Text Word] OR "deprived nation"[Text Word] OR "deprived nations"[Text Word] OR "deprived population"[Text Word] OR "deprived populations"[Text Word] OR "deprived world"[Text Word] OR "poor country"[Text Word] OR "poor countries"[Text Word] OR "poor nation"[Text Word] OR "poor nations"[Text Word] OR "poor population"[Text Word] OR "poor populations"[Text Word] OR "poor world"[Text Word] OR "poorer country"[Text Word] OR "poorer countries"[Text Word] OR "poorer nation"[Text Word] OR "poorer nations"[Text Word] OR "poorer population"[Text Word] OR "poorer populations"[Text Word] OR "poorer world"[Text Word] OR "developing economy"[Text Word] OR "developing economies"[Text Word] OR "less developed economy"[Text Word] OR "less developed economics"[Text Word] OR "lesser developed economy"[Text Word] OR "lesser developed economies"[Text Word] OR "under developed economy"[Text Word] OR "under developed economies"[Text Word] OR "underdeveloped economy"[Text Word] OR "underdeveloped economies"[Text Word] OR "middle income economy"[Text Word] OR "middle income economies"[Text Word] OR "low income economy"[Text Word] OR "low income economies"[Text Word] OR "lower income economy"[Text Word] OR "lower income economies"[Text Word] OR "low gdp"[Text Word] OR "low gnp"[Text Word] OR "low gross domestic"[Text Word] OR "low gross national"[Text Word] OR "lower gdp"[Text Word] OR "lower gnp"[Text Word] OR "lower gross domestic"[Text Word] OR "lower gross national"[Text Word] OR lmic[Text Word] OR lmics[Text Word] OR "third world"[Text Word] OR "lami country"[Text Word] OR "lami countries"[Text Word] OR "transitional country"[Text Word] OR "transitional economies"[Text Word] OR "emerging economy"[Text Word] OR "emerging economies"[Text Word] OR "emerging nation"[Text Word] OR "emerging nations"[Text Word] | |
| 56 | #1 OR #55 | 39,866 |
| 55 | #21 AND #54 | 39,859 |
| 54 | #22 OR #23 OR #24 OR #25 OR #26 OR #27 OR #28 OR #29 OR #30 OR #31 OR #32 OR #33 OR #34 OR #35 OR #36 OR #37 OR #38 OR #39 OR #40 OR #41 OR #42 OR #43 OR #44 OR #45 OR #46 OR #47 OR #48 OR #49 OR #50 OR #51 OR #52 OR #53 | 850,027 |
| 53 | "Product Surveillance, Postmarketing/statistics and numerical data"[MeSH Terms] | 2,361 |
| 52 | "Maternal Health Services/statistics and numerical data"[MeSH Terms] | 7,290 |
| 51 | "Maternal Exposure/statistics and numerical data"[MeSH Terms] | 1,123 |



For peer review only - http://bmjopen.bmj.com/site/about/guidelines.xhtml

| | | |
|---|---|---|
| 50 | pharmacovigilan*[ti] OR pharmaco-vigilan*[ti] OR pharmacovigilan*[ot] OR pharmaco-vigilan*[ot] | 3,751 |
| 49 | "Product Surveillance, Postmarketing"[mesh:noexp] | 7,553 |
| 48 | "data system"[ti] OR "data systems"[ti] OR "information system"[ti] OR "information systems"[ti] OR "data system"[ot] OR "data systems"[ot] OR "information system"[ot] OR "information systems"[ot] | 13,581 |
| 47 | "Health Information Systems"[mesh] | 1,546 |
| 46 | (decision*[ti] AND support*[ti] AND clinical*[ti]) OR (decision*[ot] AND support*[ot] AND clinical*[ot]) | 4,467 |
| 45 | "Decision Support Systems, Clinical"[mesh] | 9,106 |
| 44 | "Databases, Factual"[mesh] | 163,862 |
| 43 | "Databases as Topic"[mesh:noexp] | 9,688 |
| 42 | surveillance*[ti] OR surveillance*[ot] | 61,304 |
| 41 | "Population Surveillance"[mesh] | 74,026 |
| 40 | survey*[ti] OR survey*[ot] | 189,840 |
| 39 | "Health Surveys"[mesh:noexp] | 66,266 |
| 38 | "Health Care Surveys"[MeSH Major Topic] | 10,167 |
| 37 | "Surveys and Questionnaires"[MeSH Major Topic:noexp] | 49,876 |
| 36 | registry[ot] OR registries[ot] OR eregistr*[ot] OR "e-registry"[ot] OR "e-registries"[ot] | 8,509 |
| 35 | registry[ti] OR registries[ti] OR eregistr*[ti] OR "e-registry"[ti] OR "e-registries"[ti] | 34,311 |
| 34 | Registries[mesh:noexp] | 104,749 |
| 33 | (preliminary[ot] OR "pilot project"[ot] OR "pilot projects"[ot]) AND data[ot] | 35 |
| 32 | (preliminary[ti] OR "pilot project"[ti] OR "pilot projects"[ti]) AND data[ti] | 2,947 |
| 31 | "Preliminary Data"[mesh] | 724 |
| 30 | "focus group"[ti] OR "focus groups"[ti] OR "focus group"[ot] OR "focus groups"[ot] | 6,048 |
| 29 | "Focus Groups"[mesh] | 34,384 |
| 28 | databas*[ot] OR "data base"[ot] OR "data bases"[ot] OR databank*[ot] OR "data bank"[ot] OR "data banks"[ot] OR dataset*[ot] OR "data set"[ot] OR "data sets"[ot] | 14,502 |
| 27 | databas*[ti] OR "data base"[ti] OR "data bases"[ti] OR databank*[ti] OR "data bank"[ti] OR "data banks"[ti] OR dataset*[ti] OR "data set"[ti] OR "data sets"[ti] | 55,119 |
| 26 | "Datasets as Topic"[mesh] | 7,252 |
| 25 | data[ot] AND (accumulat*[ot] OR accura*[ot] OR assembl*[ot] OR captur*[ot] OR collect*[ot] OR compil*[ot] OR coordinat*[ot] OR co-ordinat*[ot] OR gather*[ot] OR hub[ot] OR hubs[ot]) | 5,205 |



For peer review only - http://bmjopen.bmj.com/site/about/guidelines.xhtml

| | | |
|---|---|---|
| 24 | data[ti] AND (accumulat*[ti] OR accura*[ti] OR assembl*[ti] OR captur*[ti] OR collect*[ti] OR compil*[ti] OR coordinat*[ti] OR co-ordinat*[ti] OR gather*[ti] OR hub[ti] OR hubs[ti]) | 11,365 |
| 23 | "Data Accuracy"[mesh] | 3,726 |
| 22 | "Data Collection"[mesh:noexp] | 91,761 |
| 21 | #2 OR #3 OR #4 OR #5 OR #6 OR #7 OR #8 OR #9 OR #10 OR #11 OR #12 OR #13 OR #14 OR #15 OR #16 OR #17 OR #18 OR #19 OR #20 | 1,067,642 |
| 20 | maternal[ot] AND (fetal[ot] OR fetus[ot] OR foetal[ot] OR foetus[ot]) | 3,848 |
| 19 | (maternal*[ot] OR maternity[ot]) AND (care[ot] OR health*[ot] OR service[ot] OR services[ot]) | 11,210 |
| 18 | (maternal*[ot] or maternity[ot]) AND (contact*[ot] or expos*[ot]) | 601 |
| 17 | perinatal*[ot] OR "peri-natal"[ot] OR "peri-natally"[ot] OR peripartum[ot] or "peri-partum"[ot] | 7,552 |
| 16 | prenatal*[ot] OR antenatal*[ot] OR "ante-natal"[ot] OR "ante-natally"[ot] OR antepartum[ot] or "ante-partum"[ot] | 15,862 |
| 15 | maternal[ti] AND (fetal[ti] OR fetus[ti] OR foetal[ti] OR foetus[ti]) | 13,964 |
| 14 | (maternal*[ti] OR maternity[ti]) AND (care[ti] OR health*[ti] OR service[ti] OR services[ti]) | 13,467 |
| 13 | "Maternal Health Services"[mesh:noexp] | 15,617 |
| 12 | (maternal*[ti] or maternity[ti]) AND (contact*[ti] or expos*[ti]) | 3,847 |
| 11 | "Maternal Exposure"[mesh] | 10,689 |
| 10 | perinatal*[ti] OR "peri-natal"[ti] OR "peri-natally"[ti] OR peripartum[ti] or "peri-partum"[ti] | 30,982 |
| 9 | "Perinatal Care"[mesh] | 11,334 |
| 8 | prenatal*[ti] OR antenatal*[ti] OR "ante-natal"[ti] OR "ante-natally"[ti] OR antepartum[ti] or "ante-partum"[ti] | 59,414 |
| 7 | "Prenatal Care"[mesh] | 31,249 |
| 6 | pregnan*[ti] | 252,201 |
| 5 | "Pregnancy Trimesters"[mesh] | 43,874 |
| 4 | "Pregnant Women"[mesh] | 12,334 |
| 3 | "Pregnancy Complications"[mesh] | 460,551 |
| 2 | Pregnancy[mesh] | 970,546 |
| 1 | "pregnancy exposure database" [tw] OR "pregnancy exposure databases" [tw] OR "pregnancy exposure data base" [tw] OR "pregnancy exposure data bases" [tw] OR "pregnancy exposure registry" [tw] OR "pregnancy exposure registries" [tw] | 34 |


For peer review only - http://bmjopen.bmj.com/site/about/guidelines.xhtml

**Embase**

--------------------------------------------------------------------------------
1 ("pregnancy exposure database" or "pregnancy exposure databases" or "pregnancy exposure data base" or "pregnancy exposure data bases" or "pregnancy exposure registry" or "pregnancy exposure registries").tw,kw,kf. (113)
2 exp pregnancy/ (727815)
3 exp pregnancy complication/ (141579)
4 exp named groups by pregnancy/ (130431)
5 pregnan*.ti,kw,kf. (329712)
6 exp prenatal care/ (167165)
7 (prenatal* or antenatal* or "ante-natal" or "ante-natally" or antepartum or "ante-partum").ti,kw,kf. (87488)
8 exp perinatal care/ (65235)
9 (perinatal* or peri-natal* or peripartum or "peri-partum").ti,kw,kf. (46195)
10 maternal exposure/ (3606)
11 ((maternal* or maternity) and (contact* or expos*)).ti,kw,kf. (6357)
12 maternal health service/ (2454)
13 ((maternal* or maternity) and (care or health* or service or services)).ti,kw,kf. (22738)
14 (maternal and (fetal or fetus or foetal or foetus)).ti,kw,kf. (20741)
15 or/2-14 [PREGNANCY] (1035932)
16 *information processing/ (37078)
17 data aggregation/ (374)
18 data accuracy/ (1596)
19 (data and (accumulat* or accura* or assembl* or captur* or collect* or compil* or coordinat* or co-ordinat* or gather* or hub or hubs)).ti,kw,kf. (18259)
20 (databas* or "data base" or "data bases" or databank* or "data bank" or "data banks" or dataset* or "data set" or "data sets").ti,kw,kf. (86633)
21 (focus group or focus groups).ti,kw,kf. (7608)
22 preliminary data/ (31472)
23 ((preliminary or pilot project or pilot projects) and data).ti,kw,kf. (5481)
24 exp register/ (180900)
25 (registry or registries or eregistr* or "e-registry" or "e-registries").ti,kw,kf. (68347)
26 questionnaire/ (814172)
27 *health care survey/ (2606)
28 health survey/ (213987)
29 survey*.ti,kw,kf. (220889)
30 population surveillance/ (117)
31 surveillance*.ti,kw,kf. (82221)
32 data base/ (248533)
33 factual database/ (27912)
34 clinical decision support system/ (4716)
35 (decision* and support* and clinical*).ti,kw,kf. (5904)
36 medical information system/ (22591)
37 ("data system" or "data systems" or "information system" or "information systems").ti,kw,kf. (18222)
38 exp postmarketing surveillance/ (37924)
39 (pharmacovigilan* or pharmaco-vigilan*).ti,kw,kf. (7417)



For peer review only - http://bmjopen.bmj.com/site/about/guidelines.xhtml

40 or/16-39 [DATA COLLECTION, REGISTRIES] (1815231)
41 15 and 40 [PREGNANCY - DATA COLLECTION, REGISTRIES] (71373)
42 1 or 41 [PERs, PREGNANCY - DATA COLLECTION, REGISTRIES] (71381)
43 (afghanistan or albania or algeria or american samoa or angola or "antigua and barbuda" or antigua or barbuda or argentina or armenia or armenian or aruba or azerbaijan or bahrain or bangladesh or barbados or republic of belarus or belarus or byelarus or belorussia or byelorussian or belize or british honduras or benin or dahomey or bhutan or bolivia or "bosnia and herzegovina" or bosnia or herzegovina or botswana or bechuanaland or brazil or brasil or bulgaria or burkina faso or burkina fasso or upper volta or burundi or urundi or cabo verde or cape verde or cambodia or kampuchea or khmer republic or cameroon or cameron or cameroun or central african republic or ubangi shari or chad or chile or china or colombia or comoros or comoro islands or iles comores or mayotte or democratic republic of the congo or democratic republic congo or congo or zaire or costa rica or "cote d’ivoire" or "cote d’ ivoire" or cote divoire or cote d ivoire or ivory coast or croatia or cuba or cyprus or czech republic or czechoslovakia or djibouti or french somaliland or dominica or dominican republic or ecuador or egypt or united arab republic or el salvador or equatorial guinea or spanish guinea or eritrea or estonia or eswatini or swaziland or ethiopia or fiji or gabon or gabonese republic or gambia or "georgia (republic)" or georgian or ghana or gold coast or gibraltar or greece or grenada or guam or guatemala or guinea or guinea bissau or guyana or british guiana or haiti or hispaniola or honduras or hungary or india or indonesia or timor or iran or iraq or isle of man or jamaica or jordan or kazakhstan or kazakh or kenya or "democratic people’s republic of korea" or republic of korea or north korea or south korea or korea or kosovo or kyrgyzstan or kirghizia or kirgizstan or kyrgyz republic or kirghiz or laos or lao pdr or "lao people's democratic republic" or latvia or lebanon or lebanese republic or lesotho or basutoland or liberia or libya or libyan arab jamahiriya or lithuania or macau or macao or republic of north macedonia or macedonia or madagascar or malagasy republic or malawi or nyasaland or malaysia or malay federation or malaya federation or maldives or indian ocean islands or indian ocean or mali or malta or micronesia or federated states of micronesia or kiribati or marshall islands or nauru or northern mariana islands or palau or tuvalu or mauritania or mauritius or mexico or moldova or moldovian or mongolia or montenegro or "montenegro (republic)" or morocco or ifni or mozambique or portuguese east africa or myanmar or burma or namibia or nepal or netherlands antilles or nicaragua or niger or nigeria or oman or muscat or pakistan or panama or papua new guinea or new guinea or paraguay or peru or philippines or philipines or phillipines or phillippines or poland or "polish people's republic" or portugal or portuguese republic or puerto rico or romania or russia or russian federation or ussr or soviet union or union of soviet socialist republics or rwanda or ruanda or samoa or pacific islands or polynesia or samoan islands or navigator island or navigator islands or "sao tome and principe" or saudi arabia or senegal or serbia or seychelles or sierra leone or slovakia or slovak republic or slovenia or melanesia or solomon island or solomon islands or norfolk island or norfolk islands or somalia or south africa or south sudan or sri lanka or ceylon or "saint kitts and nevis" or "st. kitts and nevis" or saint lucia or "st. lucia" or "saint vincent and the grenadines" or saint vincent or "st. vincent" or grenadines or sudan or suriname or surinam or dutch guiana or netherlands guiana or syria or syrian arab republic or tajikistan or tadjikistan or tadzhikistan or tadzhik or tanzania or tanganyika or thailand or siam or timor leste or east timor or togo or togolese republic or tonga or "trinidad and tobago" or trinidad or tobago or tunisia or "turkey (republic)" or turkey or turkmenistan or turkmen or uganda or ukraine or uruguay or uzbekistan or uzbek or vanuatu or new hebrides or venezuela or vietnam or viet nam or middle east or west bank or gaza or palestine or yemen or yugoslavia or zambia or zimbabwe or northern rhodesia or global south or africa south of the sahara or "sub saharan africa" or subsaharan africa or africa, central or central africa or africa, northern or north africa or northern africa or magreb or maghrib or sahara or africa, southern or southern africa or africa, eastern or east africa or eastern africa or africa, western or west africa or western africa or west indies or indian ocean islands or caribbean region or



For peer review only - http://bmjopen.bmj.com/site/about/guidelines.xhtml

caribbean islands or caribbean or central america or latin america or "south and central america" or south america or asia, central or central asia or asia, northern or north asia or northern asia or asia, southeastern or southeastern asia or south eastern asia or southeast asia or south east asia or asia, western or western asia or europe, eastern or east europe or eastern europe or developing country or developing countries or developing nation? or developing population? or developing world or less developed countr* or less developed nation? or less developed population? or less developed world or lesser developed countr* or lesser developed nation? or lesser developed population? or lesser developed world or under developed countr* or under developed nation? or under developed population? or under developed world or underdeveloped countr* or underdeveloped nation? or underdeveloped population? or underdeveloped world or middle income countr* or middle income nation? or middle income population? or low income countr* or low income nation? or low income population? or lower income countr* or lower income nation? or lower income population? or underserved countr* or underserved nation? or underserved population? or underserved world or under served countr* or under served nation? or under served population? or under served world or deprived countr* or deprived nation? or deprived population? or deprived world or poor countr* or poor nation? or poor population? or poor world or poorer countr* or poorer nation? or poorer population? or poorer world or developing econom* or less developed econom* or lesser developed econom* or under developed econom* or underdeveloped econom* or middle income econom* or low income econom* or lower income econom* or low gdp or low gnp or low gross domestic or low gross national or lower gdp or lower gnp or lower gross domestic or lower gross national or lmic or lmics or third world or lami countr* or transitional countr* or emerging economies or emerging nation?).ti,ab,sh,kw. (2517925)

44 ("high burden country" or "high burden countries" or "high-burden country" or "high-burden countries" or "countdown country" or "countdown countries").ti,ab,kw,kf. (808)

45 43 or 44 [LMICs] (2518149)

46 42 and 45 [PERs, PREGNANCY - DATA COLLECTION, REGISTRIES - LMICs] (18846)

47 global health/ (16219)

48 ((global or international* or world*) and health*).ti,kw,kf. (45947)

49 exp adverse drug reaction/ (586248)

50 ((drug or drugs or medicine or medicines or medication* or pharmaceutical* or pharma-ceutical*) and expos*).ti,kw,kf. (9329)

51 (ae or co).fs. (2915988)

52 (safe or safety or side effect or side effects or undesirable effect or undesirable effects or treatment emergent or tolerability or toxicity or adrs).ti,kw,kf. (514988)

53 (adverse effect or adverse effects or adverse reaction or adverse reactions or adverse event or adverse events or adverse outcome or adverse outcomes).ti,kw,kf. (70731)

54 (AEFI and adverse).ti,kw,kf. (157)

55 "Global Alignment of Immuni#ation Safety Assessment in Pregnancy".tw,kw,kf. (20)

56 (GAIA and (alignment or immuni#ation or safety or pregnan*)).tw,kw,kf. (71)

57 exp vaccination/ (205483)

58 vaccine/ (67719)

59 exp bacterial vaccine/ or exp cell-based vaccine/ or conjugate vaccine/ or edible vaccine/ or exp fungus vaccine/ or exp inactivated vaccine/ or live vaccine/ or exp meningitis vaccine/ or exp nucleic acid vaccine/ or exp parasite vaccine/ or exp peptide vaccine/ or protein vaccine/ or exp subunit vaccine/ or exp toxoid vaccine/ or exp vector vaccine/ or virosome vaccine/ or exp virus vaccine/ (304544)

60 vaccin*.ti,kw,kf. (251220)

61 immunization/ (104541)



For peer review only - http://bmjopen.bmj.com/site/about/guidelines.xhtml

62 passive immunization/ (12623)
63 (immunit* and transfer*).ti,kw,kf. (1262)
64 (immunit* and (maternally-acqui* or passive*)).ti,kw,kf. (766)
65 ((antibod* or anti-bod*) and transfer*).ti,kw,kf. (2375)
66 ((maternal or pregnan*) and immuni#ation*).ti,kw,kf. (1766)
67 exp malaria/pc [Prevention] (14654)
68 exp antimalarial agent/ (161880)
69 (antimalarial* or anti-malarial*).ti,kw,kf. (11586)
70 exp cytomegalovirus infection/pc [Prevention] (5146)
71 respiratory syncytial virus infection/pc [Prevention] (1057)
72 exp Streptococcus infection/pc [Prevention] (10568)
73 exp Zika fever/pc [Prevention] (780)
74 exp antiretrovirus agent/ (216196)
75 (antiretroviral* or anti-retroviral* or "anti-HIV" or "anti-AIDS" or "AIDS drug" or "AIDS drugs").ti,kw,kf. (46286)
76 exp fetus development/ (29871)
77 ((fetal or foetal or fetus* or foetus*) and develop*).ti,kw,kf. (14926)
78 exp prenatal exposure/ (37294)
79 ((fetal or foetal or fetus* or foetus* or prenatal* or pre-natal*) and expos*).ti,kw,kf. (16619)
80 pregnancy outcome/ (72177)
81 ((birth or births or matern* or neonat* or neo-nat* or perinatal* or peri-natal*or peripartum or "peri-partum" or postnatal* or post-natal* or postpartum or "post-partum" or post-birth* or pregnanc*) and outcome*).ti,kw,kf. (55800)
82 ((birth or births) and defect*).ti,kw,kf. (4849)
83 child health/ (32206)
84 maternal welfare/ (15842)
85 (MNCH and (maternal* or newborn* or child*)).ti,kw,kf. (49)
86 or/47-85 [DRUGS, VACCINES, SAFETY, OUTCOMES] (4592324)
87 46 and 86 [PERs, PREGNANCY - DATA COLLECTION, REGISTRIES - LMICs - DRUGS, VACCINES, SAFETY, OUTCOMES] (5663)
88 exp animal/ or exp animal experimentation/ or exp animal model/ or exp animal experiment/ or nonhuman/ or exp vertebrate/ (30677570)
89 exp human/ or exp human experimentation/ or exp human experiment/ (23737031)
90 88 not 89 (6941713)
91 87 not 90 [ANIMAL-ONLY REMOVED] (5643)
92 editorial.pt. (729310)
93 91 not 92 [EDITORIALS REMOVED] (5614)
94 limit 93 to yr="2000-current" (5299)

***************************

**CINAHL**

| # | Query | Results |
|---|---|---|
| S92 | S90 and S91 | 878 |
| S91 | DT 2000 - 2022 | 7,386,209 |



For peer review only - http://bmjopen.bmj.com/site/about/guidelines.xhtml

| | | |
|---|---|---|
| S90 | S88 NOT S89 | 885 |
| S89 | PT editorial or opinion or commentary | 684,750 |
| S88 | S45 AND S87 | 891 |
| S87 | S46 OR S47 OR S48 OR S49 OR S50 OR S51 OR S52 OR S53 OR S54 OR S55 OR S56 OR S57 OR S58 OR S59 OR S60 OR S61 OR S62 OR S63 OR S64 OR S65 OR S66 OR S67 OR S68 OR S69 OR S70 OR S71 OR S72 OR S73 OR S74 OR S75 OR S76 OR S77 OR S78 OR S79 OR S80 OR S81 OR S82 OR S83 OR S84 OR S85 OR S86 | 354,157 |
| S86 | TI (MNCH AND (maternal* OR newborn* OR child*)) | 17 |
| S85 | (MH "Maternal-Child Health") | 3,727 |
| S84 | (MH "Child Health") | 17,073 |
| S83 | TI (birth OR births) and defect* | 969 |
| S82 | TI (birth OR births OR matern* OR neonat* OR neo-nat* OR perinatal* OR peri-natal* OR peripartum OR "peri-partum" OR postnatal* OR post-natal* OR postpartum OR "post-partum" OR post-birth* OR pregnanc*) AND outcome* | 17,343 |
| S81 | (MH "Pregnancy Outcomes") | 27,127 |
| S80 | TI (fetal OR foetal OR fetus* OR foetus* or prenatal* or pre-natal*) AND expos* | 2,603 |
| S79 | (MH "Prenatal Exposure Delayed Effects") | 6,314 |
| S78 | TI (fetal OR foetal OR fetus* OR foetus*) AND develop* | 1,028 |
| S77 | (MH "Fetal Development") | 7,851 |
| S76 | TI antiretroviral* OR anti-retroviral* OR "anti-HIV" OR "anti-AIDS" OR "AIDS drug" OR "AIDS drugs" | 9,126 |
| S75 | (MH "Anti-Retroviral Agents+") | 24,911 |
| S74 | (MH "Zika Virus Infections/PC") | 168 |
| S73 | (MH "Streptococcal Infections+/PC") | 2,280 |
| S72 | (MH "Respiratory Syncytial Virus Infections/PC") | 583 |
| S71 | (MH "Hepatitis E/PC") | 69 |
| S70 | (MH "Cytomegalovirus Infections+/PC") | 509 |
| S69 | TI antimalarial* or (anti W0 malarial*) | 714 |
| S68 | (MH "Antimalarials+") | 8,320 |
| S67 | (MH "Malaria/PC") | 3,293 |



For peer review only - http://bmjopen.bmj.com/site/about/guidelines.xhtml

| | | |
|---|---|---|
| S66 | TI (maternal* or pregnan*) and immuni?ation* | 253 |
| S65 | TI ((antibod* or (anti W0 bod*)) and transfer*) | 79 |
| S64 | TI immunit* AND (passive* or "maternally-acquired") | 13 |
| S63 | TI immunit* AND transfer* | 16 |
| S62 | (MH "Immunity, Maternally Acquired") | 232 |
| S61 | (MH "Immunization Programs") | 6,426 |
| S60 | (MH "Viral Vaccines+") | 36,206 |
| S59 | (MH "Vaccines, Combined+") | 3,562 |
| S58 | (MH "Toxoids+") | 2,372 |
| S57 | (MH "Bacterial Vaccines+") | 10,296 |
| S56 | (MH "Vaccines") | 9,533 |
| S55 | (MH "Immunization+") | 31,914 |
| S54 | TI ( GAIA AND (alignment or immuni?ation or safety or pregnan*) ) OR AB ( GAIA AND (alignment or immuni?ation or safety or pregnan*) ) | 6 |
| S53 | TI ( "Global Alignment of Immunisation Safety Assessment in Pregnancy" or "Global Alignment of Immunization Safety Assessment in Pregnancy" ) OR AB ( "Global Alignment of Immunisation Safety Assessment in Pregnancy" or "Global Alignment of Immunization Safety Assessment in Pregnancy" ) | 0 |
| S52 | TI AEFI and adverse | 5 |
| S51 | TI "adverse effect" or "adverse effects" or "adverse reaction" or "adverse reactions" or "adverse event" or "adverse events" or "adverse outcome" or "adverse outcomes" | 12,615 |
| S50 | TI safe or safety or "side effect" or "side effects" or "undesirable effect" or "undesirable effects" or "treatment emergent" or tolerability or toxicity or adrs | 113,937 |
| S49 | TI (drug or drugs or medicine or medicines or medication* or pharmaceutical* or pharma-ceutical*) AND expos* | 1,420 |
| S48 | (MH "Adverse Drug Event") | 16,207 |
| S47 | TI (global or international* or world*) and health* | 22,194 |
| S46 | (MH "World Health") | 29,391 |
| S45 | S39 AND S44 | 4,402 |
| S44 | S40 OR S41 OR S42 OR S43 | 379,212 |



For peer review only - http://bmjopen.bmj.com/site/about/guidelines.xhtml

| | | |
|---|---|---|
| S43 | TI ( "high burden country" or "high burden countries" or "high-burden country" or "high-burden countries" or "countdown country" or "countdown countries" ) OR AB ( "high burden country" or "high burden countries" or "high-burden country" or "high-burden countries" or "countdown country" or "countdown countries" ) | 188 |
| S42 | TI ( "developing country" or "developing countries" or "developing nation" or "developing nations" or "developing population" or "developing populations" or "developing world" or "less developed country" or "less developed countries" or "less developed nation" or "less developed nations" or "less developed population" or "less developed populations" or "less developed world" or "lesser developed country" or "lesser developed countries" or "lesser developed nation" or "lesser developed nations" or "lesser developed population" or "lesser developed populations" or "lesser developed world" or "under developed country" or "under developed countries" or "under developed nation" or "under developed nations" or "under developed population" or "under developed populations" or "under developed world" or "underdeveloped country" or "underdeveloped countries" or "underdeveloped nation" or "underdeveloped nations" or "underdeveloped population" or "underdeveloped populations" or "underdeveloped world" or "middle income country" or "middle income countries" or "middle income nation" or "middle income nations" or "middle income population" or "middle income populations" or "low income country" or "low income countries" or "low income nation" or "low income nations" or "low income population" or "low income populations" or "lower income country" or "lower income countries" or "lower income nation" or "lower income nations" or "lower income population" or "lower income populations" or "underserved country" or "underserved countries" or "underserved nation" or "underserved nations" or "underserved population" or "underserved populations" or "underserved world" or "under served country" or "under served countries" or "under served nation" or "under served nations" or "under served population" or "under served populations" or "under served world" or "deprived country" or "deprived countries" or "deprived nation" or "deprived nations" or "deprived population" or "deprived populations" or "deprived world" or "poor country" or "poor countries" or "poor nation" or "poor nations" or "poor population" or "poor populations" or "poor world" or "poorer country" or "poorer countries" or "poorer nation" or "poorer nations" or "poorer population" or "poorer populations" or "poorer world" or "developing economy" or "developing economies" or "less developed economy" or "less developed economics" or "lesser developed economy" or "lesser developed economies" or "under developed economy" or "under developed economies" or "underdeveloped economy" or "underdeveloped economies" or "middle income economy" or "middle income economies" or "low income economy" or "low income economies" or "lower income economy" or "lower income economies" or "low gdp" or "low gnp" or "low gross domestic" or "low gross national" or "lower gdp" or "lower gnp" or "lower gross domestic" or "lower gross national" or lmic or lmics or "third world" or "lami country" or "lami | 39,219 |



For peer review only - http://bmjopen.bmj.com/site/about/guidelines.xhtml

countries" or "transitional country" or "transitional economies" or "emerging economy" or "emerging economies" or "emerging nation" or "emerging nations" ) OR AB ( "developing country" or "developing countries" or "developing nation" or "developing nations" or "developing population" or "developing populations" or "developing world" or "less developed country" or "less developed countries" or "less developed nation" or "less developed nations" or "less developed population" or "less developed populations" or "less developed world" or "lesser developed country" or "lesser developed countries" or "lesser developed nation" or "lesser developed nations" or "lesser developed population" or "lesser developed populations" or "lesser developed world" or "under developed country" or "under developed countries" or "under developed nation" or "under developed nations" or "under developed population" or "under developed populations" or "under developed world" or "underdeveloped country" or "underdeveloped countries" or "underdeveloped nation" or "underdeveloped nations" or "underdeveloped population" or "underdeveloped populations" or "underdeveloped world" or "middle income country" or "middle income countries" or "middle income nation" or "middle income nations" or "middle income population" or "middle income populations" or "low income country" or "low income countries" or "low income nation" or "low income nations" or "low income population" or "low income populations" or "lower income country" or "lower income countries" or "lower income nation" or "lower income nations" or "lower income population" or "lower income populations" or "underserved country" or "underserved countries" or "underserved nation" or "underserved nations" or "underserved population" or "underserved populations" or "underserved world" or "under served country" or "under served countries" or "under served nation" or "under served nations" or "under served population" or "under served populations" or "under served world" or "deprived country" or "deprived countries" or "deprived nation" or "deprived nations" or "deprived population" or "deprived populations" or "deprived world" or "poor country" or "poor countries" or "poor nation" or "poor nations" or "poor population" or "poor populations" or "poor world" or "poorer country" or "poorer countries" or "poorer nation" or "poorer nations" or "poorer population" or "poorer populations" or "poorer world" or "developing economy" or "developing economies" or "less developed economy" or "less developed economics" or "lesser developed economy" or "lesser developed economies" or "under developed economy" or "under developed economies" or "underdeveloped economy" or "underdeveloped economies" or "middle income economy" or "middle income economies" or "low income economy" or "low income economies" or "lower income economy" or "lower income economies" or "low gdp" or "low gnp" or "low gross domestic" or "low gross national" or "lower gdp" or "lower gnp" or "lower gross domestic" or "lower gross national" or lmic or lmics or "third world" or "lami country" or "lami countries" or "transitional country" or "transitional economies" or "emerging economy" or "emerging economies" or "emerging nation" or "emerging nations" )



For peer review only - http://bmjopen.bmj.com/site/about/guidelines.xhtml

| | | |
|---|---|---|
| S41 | TI ( mali or malta or micronesia or "federated states of micronesia" or kiribati or "marshall islands" or nauru or "northern mariana islands" or palau or tuvalu or mauritania or mauritius or mexico or moldova or moldovian or mongolia or montenegro or morocco or ifni or mozambique or "portuguese east africa" or myanmar or burma or namibia or nepal or "netherlands antilles" or nicaragua or niger or nigeria or oman or muscat or pakistan or panama or "papua new guinea" or "new guinea" or paraguay or peru or philippines or philipines or phillipines or phillippines or poland or "polish people's republic" or portugal or "portuguese republic" or "puerto rico" or romania or russia or "russian federation" or ussr or "soviet union" or "union of soviet socialist republics" or rwanda or ruanda or samoa or "pacific islands" or polynesia or "samoan islands" or "navigator island" or "navigator islands" or "sao tome and principe" or "saudi arabia" or senegal or serbia or seychelles or "sierra leone" or slovakia or "slovak republic" or slovenia or melanesia or "solomon island" or "solomon islands" or "norfolk island" or "norfolk islands" or somalia or "south africa" or "south sudan" or "sri lanka" or ceylon or "saint kitts and nevis" or "st. kitts and nevis" or "saint lucia" or "st. lucia" or "saint Vincent and the grenadines" or "saint vincent" or "st. vincent" or grenadines or sudan or suriname or surinam or "dutch guiana" or "netherlands guiana" or syria or "syrian arab republic" or tajikistan or tadjikistan or tadzhikistan or tadzhik or tanzania or tanganyika or thailand or siam or "timor leste" or "east timor" or togo or "togolese republic" or tonga or "Trinidad and tobago" or trinidad or tobago or tunisia or turkey or turkmenistan or turkmen or uganda or ukraine or uruguay or uzbekistan or uzbek or vanuatu or "new hebrides" or venezuela or vietnam or "viet nam" or "middle east" or "west bank" or gaza or palestine or yemen or yugoslavia or zambia or zimbabwe or "northern rhodesia" or "global south" or "africa south of the sahara" or "sub-saharan africa" or "subsaharan africa" or "africa, central" or "central africa" or "africa, northern" or "north africa" or "northern africa" or magreb or maghrib or sahara or "africa, southern" or "southern africa" or "africa, eastern" or "east africa" or "eastern africa" or "africa, western" or "west africa" or "western africa" or "west indies" or caribbean or "central america" or "latin america" or "south and central america" or "south america" or "asia, central" or "central asia" or "asia, northern" or "north asia" or "northern asia" or "asia, southeastern" or "southeastern asia" or "south eastern asia" or "southeast asia" or "south east asia" or "asia, western" or "western asia" or "europe, eastern" or "east europe" or "eastern europe" ) OR AB ( mali or malta or micronesia or "federated states of micronesia" or kiribati or "marshall islands" or nauru or "northern mariana islands" or palau or tuvalu or mauritania or mauritius or mexico or moldova or moldovian or mongolia or montenegro or morocco or ifni or mozambique or "portuguese east africa" or myanmar or burma or namibia or nepal or "netherlands antilles" or nicaragua or niger or nigeria or oman or muscat or pakistan or panama or "papua new guinea" or "new guinea" or paraguay or peru or philippines or philipines or phillipines or phillippines or poland or "polish people's republic" or portugal or "portuguese republic" or "puerto rico" or romania or russia or "russian federation" or ussr or "soviet union" or "union of soviet socialist republics" or rwanda or ruanda | 148,518 |



For peer review only - http://bmjopen.bmj.com/site/about/guidelines.xhtml

| | | |
|---|---|---|
| | or samoa or "pacific islands" or polynesia or "samoan islands" or "navigator island" or "navigator islands" or "sao tome and principe" or "saudi arabia" or senegal or serbia or seychelles or "sierra leone" or slovakia or "slovak republic" or slovenia or melanesia or "solomon island" or "solomon islands" or "norfolk island" or "norfolk islands" or somalia or "south africa" or "south sudan" or "sri lanka" or ceylon or "saint kitts and nevis" or "st. kitts and nevis" or "saint lucia" or "st. lucia" or "saint Vincent and the grenadines" or "saint vincent" or "st. vincent" or grenadines or sudan or suriname or surinam or "dutch guiana" or "netherlands guiana" or syria or "syrian arab republic" or tajikistan or tadjikistan or tadzhikistan or tadzhik or tanzania or tanganyika or thailand or siam or "timor leste" or "east timor" or togo or "togolese republic" or tonga or "Trinidad and tobago" or trinidad or tobago or tunisia or turkey or turkmenistan or turkmen or uganda or ukraine or uruguay or uzbekistan or uzbek or vanuatu or "new hebrides" or venezuela or vietnam or "viet nam" or "middle east" or "west bank" or gaza or palestine or yemen or yugoslavia or zambia or zimbabwe or "northern rhodesia" or "global south" or "africa south of the sahara" or "sub-saharan africa" or "subsaharan africa" or "africa, central" or "central africa" or "africa, northern" or "north africa" or "northern africa" or magreb or maghrib or sahara or "africa, southern" or "southern africa" or "africa, eastern" or "east africa" or "eastern africa" or "africa, western" or "west africa" or "western africa" or "west indies" or caribbean or "central america" or "latin america" or "south and central america" or "south america" or "asia, central" or "central asia" or "asia, northern" or "north asia" or "northern asia" or "asia, southeastern" or "southeastern asia" or "south eastern asia" or "southeast asia" or "south east asia" or "asia, western" or "western asia" or "europe, eastern" or "east europe" or "eastern europe" ) | |
| S40 | TI ( afghanistan or albania or algeria or "american samoa" or angola or antigua or barbuda or argentina or armenia or armenian or aruba or azerbaijan or bahrain or bangladesh or barbados or belarus or byelarus or belorussia or byelorussian or belize or "british honduras" or benin or dahomey or bhutan or bolivia or "bosnia herzegovina" or bosnia or herzegovina or botswana or bechuanaland or brazil or brasil or bulgaria or "burkina faso" or "burkina fasso" or "upper volta" or burundi or urundi or "cabo verde" or "cape verde" or cambodia or kampuchea or "khmer republic" or cameroon or cameron or cameroun or "central african republic" or "ubangi shari" or chad or chile or china or colombia or comoros or "comoro islands" or "iles comores" or mayotte or "democratic republic of the congo" or "democratic republic congo" or congo or zaire or "costa rica" or "cote d’ivoire" or "cote d’ ivoire" or "cote divoire" or "cote d ivoire" or "ivory coast" or croatia or cuba or cyprus or "czech republic" or czechoslovakia or djibouti or "french somaliland" or dominica or "dominican republic" or ecuador or egypt or "united arab republic" or "el salvador" or "equatorial guinea" or "spanish guinea" or eritrea or estonia or eswatini or swaziland or ethiopia or fiji or gabon or "gabonese republic" or gambia or "georgia (republic)" or georgian or ghana or "gold coast" or gibraltar or greece or grenada or guam or guatemala or guinea or "guinea bissau" or guyana or "british guiana" or haiti or hispaniola or | 230,968 |



For peer review only - http://bmjopen.bmj.com/site/about/guidelines.xhtml

| | | |
|---|---|---|
| | honduras or hungary or india or indonesia or timor or iran or iraq or "isle of man" or jamaica or jordan or kazakhstan or kazakh or kenya or "democratic people's republic of korea" or "republic of korea" or "north korea" or "south korea" or korea or kosovo or kyrgyzstan or kirghizia or kirgizstan or "kyrgyz republic" or kirghiz or laos or "lao pdr" or "lao people's democratic republic" or latvia or lebanon or lebanese republic or lesotho or basutoland or liberia or libya or "libyan arab jamahiriya" or lithuania or macau or macao or republic of "north macedonia" or macedonia or madagascar or "malagasy republic" or malawi or nyasaland or malaysia or "malay federation" or "malaya federation" or maldives or "indian ocean islands" or "indian ocean" ) OR AB ( afghanistan or albania or algeria or "american samoa" or angola or antigua or barbuda or argentina or armenia or armenian or aruba or azerbaijan or bahrain or bangladesh or barbados or belarus or byelarus or belorussia or byelorussian or belize or "british honduras" or benin or dahomey or bhutan or bolivia or "bosnia herzegovina" or bosnia or herzegovina or botswana or bechuanaland or brazil or brasil or bulgaria or "burkina faso" or "burkina fasso" or "upper volta" or burundi or urundi or "cabo verde" or "cape verde" or cambodia or kampuchea or "khmer republic" or cameroon or cameron or cameroun or "central african republic" or "ubangi shari" or chad or chile or china or colombia or comoros or "comoro islands" or "iles comores" or mayotte or "democratic republic of the congo" or "democratic republic congo" or congo or zaire or "costa rica" or "cote d'ivoire" or "cote d' ivoire" or "cote divoire" or "cote d ivoire" or "ivory coast" or croatia or cuba or cyprus or "czech republic" or czechoslovakia or djibouti or "french somaliland" or dominica or "dominican republic" or ecuador or egypt or "united arab republic" or "el salvador" or "equatorial guinea" or "spanish guinea" or eritrea or estonia or eswatini or swaziland or ethiopia or fiji or gabon or "gabonese republic" or gambia or "georgia (republic)" or georgian or ghana or "gold coast" or gibraltar or greece or grenada or guam or guatemala or guinea or "guinea bissau" or guyana or "british guiana" or haiti or hispaniola or honduras or hungary or india or indonesia or timor or iran or iraq or "isle of man" or jamaica or jordan or kazakhstan or kazakh or kenya or "democratic people's republic of korea" or "republic of korea" or "north korea" or "south korea" or korea or kosovo or kyrgyzstan or kirghizia or kirgizstan or "kyrgyz republic" or kirghiz or laos or "lao pdr" or "lao people's democratic republic" or latvia or lebanon or lebanese republic or lesotho or basutoland or liberia or libya or "libyan arab jamahiriya" or lithuania or macau or macao or republic of "north macedonia" or macedonia or madagascar or "malagasy republic" or malawi or nyasaland or malaysia or "malay federation" or "malaya federation" or maldives or "indian ocean islands" or "indian ocean" ) | |
| S39 | S1 OR S38 | 15,244 |
| S38 | S16 AND S37 | 15,243 |
| S37 | S17 OR S18 OR S19 OR S20 OR S21 OR S22 OR S23 OR S24 OR S25 OR S26 OR S27 OR S28 OR S29 OR S30 OR S31 OR S32 OR S33 OR S34 OR S35 OR S36 | 354,620 |



For peer review only - http://bmjopen.bmj.com/site/about/guidelines.xhtml

| | | |
|---|---|---|
| S36 | (MH "Product Surveillance+/SN") | 1 |
| S35 | (MH "Maternal Health Services+/SN") | 1,245 |
| S34 | TI pharmacovigilan* or (pharmaco W0 vigilan*) | 846 |
| S33 | (MH "Product Surveillance+") | 2,417 |
| S32 | TI "data system" or "data systems" or "information system" or "information systems" | 4,466 |
| S31 | (MH "Health Information Systems") | 3,566 |
| S30 | TI (decision* AND support* AND clinical*) | 1,865 |
| S29 | (MH "Decision Support Systems, Clinical") | 6,139 |
| S28 | TI surveillance* | 15,975 |
| S27 | (MH "Population Surveillance+") | 11,277 |
| S26 | TI survey* | 71,609 |
| S25 | (MH "Surveys") | 157,567 |
| S24 | TI registry or registries or eregistr* or "e-registry" or "e-registries" | 13,946 |
| S23 | TI (preliminary or "pilot project" or "pilot projects") AND data | 682 |
| S22 | TI "focus group" or "focus groups" | 2,957 |
| S21 | (MH "Focus Groups") | 48,414 |
| S20 | TI databas* or "data base" or "data bases" or databank* or "data bank" or "data banks" or dataset* or "data set" or "data sets" | 16,019 |
| S19 | TI data AND (accumulat* or accura* or assembl* or captur* or collect* or compil* or coordinat* or co-ordinat* or gather* or hub or hubs) | 4,425 |
| S18 | (MH "Data Curation") | 184 |
| S17 | (MH "Data Collection") | 49,942 |
| S16 | S2 OR S3 OR S4 OR S5 OR S6 OR S7 OR S8 OR S9 OR S10 OR S11 OR S12 OR S13 OR S14 OR S15 | 290,175 |
| S15 | TI maternal AND (fetal or fetus or foetal or foetus) | 4,004 |
| S14 | TI (maternal* or maternity) AND (care or health* or service or services) | 9,718 |
| S13 | (MH "Maternal Health Services") | 11,199 |
| S12 | TI (maternal* or maternity) AND (contact* or expos*) | 1,028 |
| S11 | (MH "Maternal Exposure") | 2,588 |


For peer review only - http://bmjopen.bmj.com/site/about/guidelines.xhtml

| | | |
|---|---|---|
| S10 | TI perinatal* or "peri-natal" or "peri-natally" or peripartum or "peri-partum" | 12,858 |
| S9 | (MH "Perinatal Care") | 5,019 |
| S8 | TI prenatal* or antenatal* or "ante-natal" or "ante-natally" or antepartum or "ante-partum" | 20,877 |
| S7 | (MH "Prenatal Care") | 19,422 |
| S6 | TI pregnan* | 80,123 |
| S5 | (MH "Pregnancy Trimesters+") | 13,521 |
| S4 | (MH "Expectant Mothers") | 11,382 |
| S3 | (MH "Pregnancy Complications+") | 106,825 |
| S2 | (MH "Pregnancy") OR (MH "Pregnancy, Multiple+") OR (MH "Pregnancy, Unplanned") OR (MH "Pregnancy, Unwanted") OR (MH "Pregnancy, High Risk") OR (MH "Pregnancy, Prolonged") | 227,240 |
| S1 | TI ( "pregnancy exposure database" OR "pregnancy exposure databases" OR "pregnancy exposure data base" OR "pregnancy exposure data bases" OR "pregnancy exposure registry" OR "pregnancy exposure registries" ) OR AB ( "pregnancy exposure database" OR "pregnancy exposure databases" OR "pregnancy exposure data base" OR "pregnancy exposure data bases" OR "pregnancy exposure registry" OR "pregnancy exposure registries" ) | 7 |

**Global Index Medicus**

tw:((tw:(pregnan* exposure* database*)) OR (tw:(pregnan* exposure* "data base")) OR (tw:(pregnan* exposure* "data bases")) OR (tw:(pregnan* exposure* databank*)) OR (tw:(pregnan* exposure* "data bank")) OR (tw:(pregnan* exposure* "data banks")) OR (tw:(pregnan* exposure* register)) OR (tw:(pregnan* exposure* registries)) OR (tw:(pregnan* exposure* registry)) OR (tw:(pregnan* exposure* registries))) – *108 records*

tw:((ti:((pregnan* OR prenatal* OR antenatal* OR "ante-natal" OR "ante-natally" OR antepartum OR "ante-partum" OR perinatal* OR "peri-natal" OR "peri-natally" OR peripartum OR "peri-partum" OR maternal*) AND (data OR database* OR databank* OR register OR registers OR registry OR registries OR survey* OR surveillance OR pharmacovigilan* OR "pharmaco-vigilance") AND (safe OR safety OR "side effect" of "side effects" OR "undesirable effect" OR "undesirable effects" OR "treatment emergent" OR tolerability OR toxicity OR adrs OR aefi)))) – *361 records*

tw:(ti:((pregnan* OR prenatal* OR antenatal* OR "ante-natal" OR "ante-natally" OR antepartum OR "ante-partum" OR perinatal* OR "peri-natal" OR "peri-natally" OR peripartum OR "peri-partum" OR maternal*) AND (data OR database* OR databank* OR register OR registers OR registry OR registries OR survey* OR surveillance OR pharmacovigilan* OR "pharmaco-vigilance") AND (MNCH or "maternal health" or "child health" or "infant health" or "birth defect" or "birth defects" or "birth outcome" or



For peer review only - http://bmjopen.bmj.com/site/about/guidelines.xhtml

"birth outcomes" or "pregnancy outcome" or "pregnancy outcomes" or "neonatal outcome" or "neonatal outcomes"))) – *50 records*

tw:(ti:((pregnan* OR prenatal* OR antenatal* OR "ante-natal" OR "ante-natally" OR antepartum OR "ante-partum" OR perinatal* OR "peri-natal" OR "peri-natally" OR peripartum OR "peri-partum" OR maternal*) AND (data OR database* OR databank* OR register OR registers OR registry OR registries OR survey* OR surveillance OR pharmacovigilan* OR "pharmaco-vigilance") AND (vaccine or vaccines or vaccination* or immunization* or immunization* or “maternally-acquired”))) – *0 records*

tw:((ti:((pregnan* OR prenatal* OR antenatal* OR "ante-natal" OR "ante-natally" OR antepartum OR "ante-partum" OR perinatal* OR "peri-natal" OR "peri-natally" OR peripartum OR "peri-partum" OR maternal*) AND (data OR database* OR databank* OR register OR registers OR registry OR registries OR survey* OR surveillance OR pharmacovigilan* OR "pharmaco-vigilance") AND (drug OR drugs OR medicine OR medicines OR medication* OR pharmaceutical* OR "pharma-ceutical" OR "pharma-ceuticals" OR vaccin* OR immun* OR antimalarial* OR "anti-malarial" OR "anti-malarials" OR antiviral* OR "anti-viral" OR "anti-virals" OR antiretroviral* OR "anti-retroviral" OR "anti-retrovirals" OR "Anti-HIV" OR "Anti-AIDS")))) – *10 records*

tw:((ti:((pregnan* OR prenatal* OR antenatal* OR "ante-natal" OR "ante-natally" OR antepartum OR "ante-partum" OR perinatal* OR "peri-natal" OR "peri-natally" OR peripartum OR "peri-partum" OR maternal*) AND (data OR database* OR databank* OR register OR registers OR registry OR registries OR survey* OR surveillance OR pharmacovigilan* OR "pharmaco-vigilance") AND (fetal* OR foetal* OR fetus* OR foetus* OR neonat* OR newborn* OR infant OR infants OR infanc* OR child*) ))) – *125 records*

tw:((ti:((pregnan* OR prenatal* OR antenatal* OR "ante-natal" OR "ante-natally" OR antepartum OR "ante-partum" OR perinatal* OR "peri-natal" OR "peri-natally" OR peripartum OR "peri-partum" OR maternal*)  AND (data OR database* OR databank* OR register OR registers OR registry OR registries OR survey* OR surveillance OR pharmacovigilan* OR "pharmaco-vigilance"))) AND (tw:(drug OR drugs OR medicine OR medicines OR medication* OR pharmaceutical* OR "pharma-ceutical" OR "pharma-ceuticals" OR vaccin* OR immunisation* OR immunization* OR antimalarial* OR "anti-malarial" OR "anti-malarials" OR antiviral* OR "anti-viral" OR "anti-virals" OR antiretroviral* OR "anti-retroviral" OR "anti-retrovirals" OR "Anti-HIV" OR "Anti-AIDS"))) – *71 records*

**TOTAL: 725 records**

## Google Scholar

"pregnant|pregnancy|prenatal"+"registry|registries|surveillance|pharmacovigilance" +

"expose|exposed|exposes|exposure|exposures"+[names of LMICs]



For peer review only - http://bmjopen.bmj.com/site/about/guidelines.xhtml

**Abbreviations**

| Abbreviation | Definition |
|---|---|
| DCVMN | Developing Countries Vaccine Manufactures Network |
| EMA | European Medicine Agency |
| GAIA | Global Alignment of Immunization Safety Assessment in pregnancy |
| HICs | High Income Countries |
| ICD | International Classification of Disease |
| JBI | Joanna Briggs Instituite |
| LMICs | Low and Middle-Income Countries |
| MedDRA | Medical Dictionary for Regulatory Activities |
| MeSH | Medical Subject Headings |
| MI | Maternal Immunization |
| MNCH | Maternal, Newborn, and Child Health |
| NIH | National Institute of Health |
| OSF | Open Science Framework |
| PER | Pregnancy Exposure Registry |
| PRISMA-ScR | Preferred Reporting Items for Systematic reviews and Meta-Analyses extension for Scoping Reviews |
| RSV | Respiratory Syncytial Virus |
| UK | United Kingdom |
| US FDA | United States Food and Drug Administration |
| WHO | World Health Organization |



For peer review only - http://bmjopen.bmj.com/site/about/guidelines.xhtml

## References

[1] White NJ, McGready RM, Nosten FH (2008) New Medicines for Tropical Diseases in Pregnancy: Catch-22. PLoS Med 2008;5(6): e133.

[2] David AL, Ahmadzia H, Ashcroft R, Bucci-Rechtweg C, Spencer RN, Thornton S. Improving Development of Drug Treatments for Pregnant Women and the Fetus. Ther Innov Regul Sci. 2022 Nov;56(6):976-990.

[3] Gelperin K, Hammad H, Leishear K, Bird ST, Taylor L, Hampp C, Sahin L. A systematic review of pregnancy exposure registries: examination of protocol-specified pregnancy outcomes, target sample size, and comparator selection. Pharmacoepidemiol Drug Saf. 2017 Feb;26(2):208-214.

[4] Roque Pereira L, Durán CE, Layton D, Poulentzas G, Lalagkas PN, Kontogiorgis C, Sturkenboom M. A Landscape Analysis of Post-Marketing Studies Registered in the EU PAS Register and ClinicalTrials.gov Focusing on Pregnancy Outcomes or Breastfeeding Effects: A Contribution from the ConcePTION Project. Drug Saf. 2022 Apr;45(4):333-344.

[5] Tinto H, Sevene E, Dellicour S, Calip GS, d'Alessandro U, Macete E, Nakanabo-Diallo S, Kazienga A, Valea I, Sorgho H, Valá A, Augusto O, Ruperez M, Menendez C, Ouma P, Desai M, ter Kuile F, Stergachis A. Assessment of the safety of antimalarial drug use during early pregnancy (ASAP): protocol for a multicenter prospective cohort study in Burkina Faso, Kenya and Mozambique. Reproductive Health. 2015 Dec, 12:112.

[6] Mehta U, Clerk C, Allen E, Yore M, Sevene E, Singlovic J, Petzold M, Mangiaterra V, Elefant E, Sullivan FM, Holmes LB, Gomes M. Protocol for a drugs exposure pregnancy registry for implementation in resource-limited settings. BMC Pregnancy Childbirth. 2012 Sep 3;12:89.

[7] Clinicaltrials.gov. COVID-19 Vaccines International Pregnancy Exposure Registry (C-VIPER). https://clinicaltrials.gov/ct2/show/NCT04705116. Accessed October 25, 2022.

[8] Vargesson N. Thalidomide-induced teratogenesis: History and mechanisms. Birth Defects Res C Embryo Today. 2015;105(2):140-156.

[9] Engmann C, Fleming JA, Khan S, et al. Closer and closer? Maternal immunization: current promise, future horizons. J Perinatol. 2020;40(6):844-857. doi:10.1038/s41372-020-0668-3

[10] Sobanjo-ter Meulen A, Munoz FM, Kaslow DC, Klugman KP, Omer SB, Vora P, Stergachis A. Maternal Interventions Vigilance Harmonization in Low- and Middle-Income Countries: Stakeholder Meeting Report; Amsterdam, May 1-2, 2018. Vaccine. 2019 May 6;37(20):2643-2650.

[11] Gelperin K, Hammad H, Leishear K, Bird ST, Taylor L, Hampp C, Sahin L. A systematic review of pregnancy exposure registries: examination of protocol-specified pregnancy outcomes,



For peer review only - http://bmjopen.bmj.com/site/about/guidelines.xhtml

target sample size, and comparator selection. Pharmacoepidemiol Drug Saf. 2017 Feb;26(2):208-214.

[12] Berrueta M, Ciapponi A, Bardach A, Cairoli FR, Castellano FJ, Xiong X, Stergachis A, Zaraa S, Sobanjo-ter Meulen A, Buekens P and Scoping Review Collaboration Group. Maternal and neonatal data collection systems in low- and middle-income countries for maternal vaccines active safety surveillance systems: A scoping review. BMC Pregnancy and Childbirth. 2021 Mar 17;21:217.

[13] Stuurman AL, Riera M, Lamprianou S, Perez-Vilar S, Anderson SA, Mangtani P, Devlieger H, Verstraeten T, Zuber PLF, Guillard Maure C. Vaccine safety surveillance in pregnancy in low- and middle-income countries using GAIA case definitions: A feasibility assessment. Vaccine. 2018 Oct 29;36(45):6736-6743.

[14] Gliklich RE, Dreyer NA, Leavy MB, editors. Registries for Evaluating Patient Outcomes: A User's Guide [Internet]. 3rd edition. Rockville (MD): Agency for Healthcare Research and Quality (US); 2014 Apr. 21, Pregnancy Registries. Available from: https://www.ncbi.nlm.nih.gov/books/NBK208605

[15] Chapter 11: Scoping reviews - JBI Manual for Evidence Synthesis - JBI Global Wiki. Accessed March 7, 2022. https://jbi-global-wiki.refined.site/space/MANUAL/3283910770/Chapter+11%3A+Scoping+reviews.

[16] McGowan J, Sampson M, Salzwedel DM, Cogo E, Foerster V, Lefebvre C. PRESS Peer Review of Electronic Search Strategies: 2015 Guideline Statement. J Clin Epidemiol. 2016;75:40-46. doi:10.1016/j.jclinepi.2016.01.021.

[17] Tricco AC, Lillie E, Zarin W, et al. PRISMA Extension for Scoping Reviews (PRISMA-ScR): Checklist and Explanation. Ann Intern Med. 2018;169(7):467-473. doi:10.7326/M18-0850.

[18] OSF Registries. Accessed July 8, 2022. https://osf.io/registries.

[19] World Bank Country and Lending Groups. World Bank. https://datahelpdesk.worldbank.org/knowledgebase/articles/906519-world-bank-country-and-lending-groups. Accessed July 8, 2022.



For peer review only - http://bmjopen.bmj.com/site/about/guidelines.xhtml

BMJ Open

# Pregnancy Exposure Registries for Drugs and Vaccines in Low- and Middle-Income Countries: Scoping Review Protocol

| | |
|---|---|
| Journal: | *BMJ Open* |
| Manuscript ID | bmjopen-2022-070543.R1 |
| Article Type: | Protocol |
| Date Submitted by the Author: | 22-Mar-2023 |
| Complete List of Authors: | AbuShweimeh, Rahmeh; University of Washington, School of Public Health<br>Knudson, Sophie; PATH, Center for Vaccine Innovation and Access<br>Chaabane, Sonia; World Health Organization, Regulation and Safety<br>Pal, Shanthi Narayan; World Health Organization<br>Skidmore, Becky; 30. Independent Information Specialist<br>Stergachis, Andy; University of Washington School of Public Health, Pharmacy;<br>Bhat, Niranjan ; Center for Vaccine Innovation and Access, PATH, |
| <b>Primary Subject Heading</b>: | Obstetrics and gynaecology |
| Secondary Subject Heading: | Epidemiology, Pharmacology and therapeutics |
| Keywords: | CLINICAL PHARMACOLOGY, Maternal medicine < OBSTETRICS, EPIDEMIOLOGY |
| | |

SCHOLARONE™
Manuscripts

# Pregnancy Exposure Registries for Drugs and Vaccines in Low- and Middle-Income Countries: Scoping Review Protocol

**Authors:** Rahmeh AbuShweimeh[1], Sophia Knudson[2], Sonia Chaabane[3], Shanthi Narayan Pal[3], Becky Skidmore,[4] Andy Stergachis[1,5], Niranjan Bhat[2]

**Affiliations:**

1. School of Public Health, Hans Rosling Center for Population Health, University of Washington, 3980 15th Ave NE, Seattle, WA 98195
2. PATH, 2201 Westlake Ave, Suite 200, Seattle, WA 98121
3. Regulation and Safety, World Health Organization, Avenue Appia 20, 1211, Geneva, Switzerland
4. Independent Information Specialist, Ottawa, Canada
5. School of Pharmacy, University of Washington, Box 357631, Seattle, WA 98195

**Corresponding Author:**

Dr. Andy Stergachis; stergach@uw.edu

## Abstract

***Introduction**:* Data regarding the safety of drugs and vaccines in pregnant women are typically unavailable before licensure. Pregnancy exposure registries (PERs) are an important source of post-marketing safety information. PERs in low- and middle-income countries (LMICs) are uncommon but can provide valuable safety data from their distinct contexts and will become more relevant as the use of new drugs and vaccines in pregnancy increase worldwide. Strategies to support PERs in LMICs must be based on a better understanding of their current status. We



For peer review only

developed a scoping review protocol to assess the landscape of PERs that operate in LMICs and characterize their strengths and challenges.

***Methods and Analysis*** This scoping review follows the Joanna Briggs Institute (JBI) manual for scoping reviews. The search strategy will be reported using the Preferred Reporting Items for Systematic reviews and Meta-Analyses extension for Scoping Reviews (PRISMA-ScR) Checklist. We will search PubMed, Embase, CINAHL, and WHO's Global Index Medicus, as well as the reference lists of retrieved full-text records, for articles published between 2000 and 2022 that describe PERs or other resources that systematically record exposures to medical products during pregnancy and maternal and infant outcomes in LMICs. Title and abstracts will be screened by two authors and data extracted using a standardized form. We will undertake a grey literature search using Google Scholar and targeted websites. We will distribute an online survey to and conduct semi-structured interviews with selected experts and key informants. Identified PERs will be summarized in tables and analyzed.

***Ethics and dissemination*** Ethical approval is not required for this activity, as it was determined not to involve human-subjects research. Findings will be submitted to an open access peer-reviewed journal and may be presented at conferences, with underlying data and other materials made publicly available.

***Registration*** Open Science Framework (DOI: https://doi.org/10.17605/OSF.IO/FU5AT).



For peer review only

For peer review only - http://bmjopen.bmj.com/site/about/guidelines.xhtml

## STRENGTHS AND LIMITATIONS OF THIS STUDY

- This scoping review identify the landscape of pregnancy exposure registries that operate in low- and middle-income countries and characterize their strengths and challenges for use in assessing the safety of new vaccines introductions.
- The methodology is guided by Joanna Briggs Institute Scoping Review Manual and reporting by PRISMA checklist and informed by an expert technical working group.
- To augment limitations with bibliographic database and grey literature searches, experts and key informants will identify additional pregnancy exposure registries and systems that that record exposure to medical products during pregnancy and maternal and perinatal outcomes in low- and middle-income countries.

For peer review only



For peer review only - http://bmjopen.bmj.com/site/about/guidelines.xhtml

## Introduction

Newly introduced vaccines and drugs hold the promise of reducing morbidity and mortality among pregnant women and infants living in low- and middle-income countries (LMICs). However, since pregnant women are actively excluded from most pre-registration clinical trials, safety information for this group is rarely available at the time of a medical product's licensure or approval. (1,2) Consequently, the safety of drugs and vaccines administered during pregnancy must be evaluated throughout their life cycle, including through active surveillance approaches during the post-licensure or post-authorization phase. A commonly used method to systematically assess post-approval safety of drugs and vaccines in pregnant women and their offspring is through the use of a pregnancy exposure registry (PER). A PER is an observational study that systematically collects health information on exposure to medical products such as drugs and vaccines during pregnancy.

PERs, particularly in high-income countries (HICs), are used throughout the post-marketing phase to monitor the safety of drugs and vaccines used during pregnancy. (3,4) Pregnancy exposure registries have been used infrequently in LMICs, where there are unique challenges with respect to knowledge of background rates for obstetric and neonatal outcomes, access to the interventions under evaluation, and the availability of data collection resources and infrastructure. This latter point underscores the limitations in capturing information on the use of vaccines and other medicines in pregnancy, as well as information on the occurrence of obstetric/perinatal complications and other outcomes, and the capacity to link these data sources together[2]. Examples of pregnancy exposure registries in LMICs include those established for



For peer review only - http://bmjopen.bmj.com/site/about/guidelines.xhtml

drugs or vaccines of particular relevance for their populations, such as those used to treat malaria and HIV and prevent COVID-19. (5-7) Maternal immunization (MI) has been shown to be an effective method to protect women during pregnancy and their newborns, and use in LMICs is growing. (8) MI is anticipated to increase further in the coming years as a result of the continued adoption of COVID-19 vaccines and the introduction of promising new MI-specific vaccines, such as those for respiratory syncytial virus (RSV) and group B *Streptococcus* (GBS). Pre-licensure safety data on these vaccines will largely come from HIC settings, highlighting the importance of monitoring their post-marketing safety in LMICs. (9,10)

Most research assessing the global status of drug and vaccine safety monitoring in pregnancy has focused on HICs. (11) However, one recent study assessed existing maternal, newborn, and child health (MNCH) data collection systems in LMICs that could be used to monitor drug or vaccine safety and another study assessed the feasibility of use of Global Alignment of Immunization Safety Assessment in pregnancy (GAIA) case definitions for neonatal outcomes and maternal vaccination in LMICs. (12, 13) In contrast to these broader surveillance systems, PERs focus on the active data collection specifically related to medical product exposures and safety outcomes and may be conducted by private as well as public agencies. (14) An improved understanding of PERs in LMICs can better inform how future public health efforts in their maternal populations, such as new vaccine introductions and treatment programs, can be supported. To address this need, we aim to conduct a scoping review to identify and describe PERs and other similar resources that operate in LMICs. Here, we report the methodology for our planned scoping review.



For peer review only - http://bmjopen.bmj.com/site/about/guidelines.xhtml

## Study objective

This scoping review aims to identify pregnancy exposure registries, databases and other routinely collected health data that systematically record exposures to medical products during pregnancy and maternal and infant outcomes in LMICs.  . This scoping review will consist of a systematic search of the scientific and grey literature, supplemented by an online survey and interviews with selected key informants, as needed.

## Method

### Protocol design

This scoping review follows the Joanna Briggs Institute (JBI) manual for scoping reviews, and the search strategy will be reported using the Preferred Reporting Items for Systematic reviews and Meta-Analyses extension for Scoping Reviews (PRISMA-ScR) Checklist. (15-17)  This protocol has been registered with the Open Science Framework (DOI: https://doi.org/10.17605/OSF.IO/FU5AT). (18)  The scoping review start and estimated end dates are July 1, 2022 and June 30, 2023, respectively.

### Review questions

Following multiple consultations with key stakeholders, three primary review questions were selected:

1. What pregnancy exposure cohorts, databases and registries exist in LMICs?
2. What types of data, processes, and tools are included in these databases and registries?
3. What are the strengths and weaknesses of the identified databases and registries?



For peer review only - http://bmjopen.bmj.com/site/about/guidelines.xhtml

In addition, two secondary questions were identified:

1. Can the pregnancy exposure registries that have been identified in LMICs be used or adapted to monitor additional new vaccines or drugs that may be introduced for pregnant women?
2. What is the potential for data harmonization and/or combining of data across databases and registries?

Based on these objectives, the following eligibility criteria for selection were developed:

**Inclusion criteria**

1. Publications and documents published or produced from January 2000 to the present, to ensure that identified registries possess features that are more relevant to current scientific and technological conditions; online sources will be accessed.
2. Populations studied are located entirely or at least partially in low- and middle-income countries. (19)
3. Reference to prospective and retrospective electronic or combined paper-electronic data collection systems including demographic national registers in LMICs.
4. Reference to prospective and retrospective cohort studies, with no restrictions regarding age range other than women of childbearing age (i.e., 15-49 years of age) or underlying conditions other than pregnancy.
5. Reference to systems that collect data on exposure to one or more drugs or vaccines during pregnancy.
6. Reference to systems that collect data on pregnancy outcomes, including delivery, post-partum, and neonatal (may include an extended time frame to include birth defects detected later).



For peer review only - http://bmjopen.bmj.com/site/about/guidelines.xhtml

**Exclusion criteria**

1. Editorials, opinion pieces, promotional literature
2. Guidelines or guidance documents.
3. Reference to non-allopathic (e.g., traditional, homeopathic, or naturopathic) interventions

**Search strategy and information sources**

Using an iterative process and in consultation with the review team, an information specialist will develop a strategy in PubMed incorporating controlled vocabulary/Medical Subject Headings (e.g., "Pregnancy", "Datasets as Topic", "Product Surveillance, Postmarketing") and free text (e.g., prenatal, registries, pharmacovigilance) (see Appendix 1). We will apply a LMIC filter to focus results to the geographic regions of interest. Another information specialist will peer review the strategy using the PRESS Checklist (6). Any necessary edits will be made before finalizing in PubMed and subsequently translating the search to Embase, CINAHL, and WHO's Global Index Medicus. We will also search the reference lists of potentially relevant records and articles to ensure that our search results are as comprehensive as possible.

Additionally, we will undertake a grey literature search, including Google Scholar search and relevant websites, such as industry and professional organizations, associations and alliances (e.g., Developing Countries Vaccine Manufactures Network (DCVMN), Society for Maternal-Fetal Medicine, American College of Obstetricians and Gynecologists, International Society for Pharmacoepidemiology, International Society of Pharmacovigilance); selected Ministries of Health (including regulatory agencies and pharmacovigilance centers) in LMICs; and selected HIC organizations, including US FDA, EMA, NIH, World Health Organization, Maternal and



For peer review only - http://bmjopen.bmj.com/site/about/guidelines.xhtml

Child Survival Programs, Measure Evaluation, UK Teratology Information Service, UK Obstetric Surveillance System, and selected academic and other non-governmental groups. The final search strategy is provided in the appendix.

## Study selection

Records retrieved by the search strategy will be downloaded to EndNote Version 9.3.3 (Clarivate) for de-duplication and then uploaded to review management software (Covidence) to enable independent screening and track disagreements and consensuses among reviewers. Each title and abstract will be screened by two independent reviewer authors to determine eligibility. Each item will be categorized into one of three categories (Yes, Maybe, No), and disagreements between reviewers will be resolved by a third reviewer. After title and abstract screening, a second round of screening will be conducted for full-text review by two reviewers and a decision will be made for data extraction. An adapted version of the PRISMA flow diagram will be constructed to summarize the number of records screened, assessed for eligibility, and included in the review, with reasons for exclusions at each stage. (7)

### Data extraction

A form for data extraction will be used to extract key information regarding the registries from the selected full-text articles and screened grey literature (see Appendix 2). The form was pilot-tested and refined during the full-text screening stage in order to capture information more efficiently. Key data elements to be collected from the included articles include the following:

a. Author(s)
b. Year of publication
c. Country(ies) where the registry is located.



For peer review only - http://bmjopen.bmj.com/site/about/guidelines.xhtml

d. Name and aims/purpose of the registry.

e. Years of registry operation

f. Characteristics of the included population (specifically, the characteristics or eligibility criteria used to be included in the registry)

g. Country representativeness (local/national/regional)

h. Funding source for the registry.

i. Current sample size (proportion in LMIC)

j. Methodology/methods, including database type.

k. Terminology and data system used (e.g., MedDRA, ICD, Brighton Collaboration)

l. Intervention/Exposure type (e.g., drugs, vaccines), comparator and details of these, including trimester of exposure.

m. Maternal, perinatal, and neonatal outcomes including, but not limited to spontaneous abortions, stillbirths, congenital anomalies, and details of these. In addition, documenting whether GAIA definitions or other standardized classifications are used.

n. Duration of follow-up.

o. Key findings that relate to the scoping review questions, including strengths, weaknesses, ability to add new interventions, and ability to combine data with other systems.

p. Demographic factors, socioeconomic factors, and lifestyle factors.

Data will be entered into a database that will allow searching and categorization according to selected characteristics.



For peer review only - http://bmjopen.bmj.com/site/about/guidelines.xhtml

**Informant Survey and Interviews**

In addition to searching bibliographic databases and grey literature, an online survey will be sent to experts and key informants to identify additional pregnancy exposure registries, surveillance registries, databases and routinely collected data that record exposure to medical products during pregnancy and maternal and perinatal outcomes in LMICs that may not have been captured or to provide additional detail on those already identified. Information obtained through key informant interviews will be subject to the same inclusion and exclusion criteria as used with the literature. A subset of survey respondents will be identified for semi-structured interviews if additional information is needed about the registries. Interviewees will be asked to discuss the strengths and weaknesses of the registry, relevant contextual factors, and the usability of these resources in their particular settings. All responses will be recorded in an electronic database for analysis.

**Data analysis**

Identified PERs will be summarized in tables organized according to relevant characteristics, including geography, methodology, types of interventions included, outcomes captured, and citations. The selected PERs will be further grouped and evaluated based on the primary and secondary scoping review questions (strengths, weaknesses, ability to add new interventions, and ability to combine data with other systems) and other methods of appraisal, and the quality of the existing registries may be discussed as part of the findings. Geographic coverage or other spatial characteristics may be presented using maps.



For peer review only - http://bmjopen.bmj.com/site/about/guidelines.xhtml

### Consultation

As part of this activity, a technical working group was established to provide assistance and guidance throughout the course of the review. Members of this group are experts in multiple disciplines, including pharmacovigilance, perinatology, and pediatrics, particularly as practiced in low-resource settings. In addition to this group, the protocol and results of this scoping review will be reviewed by an Expert Steering Committee on Safety Surveillance in Pregnancy in LMICs, established by WHO. Feedback from both groups will be incorporated to produce a final document.

## Patient and public involvement

There will be no patient or public involvement in this scoping review.

## Dissemination and ethics

Ethical approval is not required for this activity, though it has been reviewed by PATH's Research Determination Committee and deemed not research nor human-subjects research. Results of this landscape analysis will be submitted to an open-access peer-reviewed journal for publication and may be presented at conferences, while underlying data and other materials will be made publicly available.

## Funding

This work was supported by a grant from the Bill & Melinda Gates Foundation [grant number INV-037810]. The funders had no role in the design, analysis, or writing of this manuscript.



For peer review only

## Acknowledgments

The authors would like to thank Kaitryn Campbell for peer review of the PubMed search strategy. We also thank the members of our Technical Working Group: Pierre Buekens, Ushma Mehta, Deshayne Fell, Smaragda Lamprianou, and Kate Fay, Ajoke ter Meulen, Nancy Salt, and Jessica Fleming. The authors alone are responsible for the views expressed in this article and they do not necessarily represent the views, decisions or policies of the institutions with which they are affiliated.

## Competing Interest

No competing interest.

## Author Contributions Statement

NB was responsible for conception and design of the review. BS developed the search strategies. SK and RAS are conducting the initial review of manuscripts. NB and AS are second reviewers. SC and SNP provided expert review and input into the protocol. RAS and AS were responsible for producing the initial draft of the review protocol. All authors provided significant editorial comments on the protocol drafts and read and approved the final manuscript.

For peer review only

## References

1. White NJ, McGready RM, Nosten FH. New medicines for tropical diseases in pregnancy: Catch-22. PLoS Med 2008;5(6): e133.

2. David AL, Ahmadzia H, Ashcroft R, Bucci-Rechtweg C, Spencer RN, Thornton S. Improving development of drug treatments for pregnant women and the fetus. Ther Innov Regul Sci. 2022 Nov;56(6):976-990.

3. Gelperin K, Hammad H, Leishear K, Bird ST, Taylor L, Hampp C, Sahin L. A systematic review of pregnancy exposure registries: examination of protocol-specified pregnancy outcomes, target sample size, and comparator selection. Pharmacoepidemiol Drug Saf. 2017 Feb;26(2):208-214.

4. Roque Pereira L, Durán CE, Layton D, Poulentzas G, Lalagkas PN, Kontogiorgis C, Sturkenboom M. A Landscape Analysis of Post-Marketing Studies Registered in the EU PAS Register and ClinicalTrials.gov Focusing on Pregnancy Outcomes or Breastfeeding Effects: A Contribution from the ConcePTION Project. Drug Saf. 2022 Apr;45(4):333-344.

5. Tinto H, Sevene E, Dellicour S, Calip GS, d'Alessandro U, Macete E, Nakanabo-Diallo S, Kazienga A, Valea I, Sorgho H, Valá A, Augusto O, Ruperez M, Menendez C, Ouma P, Desai M, ter Kuile F, Stergachis A. Assessment of the safety of antimalarial drug use during early pregnancy (ASAP): protocol for a multicenter prospective cohort study in Burkina Faso, Kenya and Mozambique. Reproductive Health. 2015 Dec, 12:112.

6. Mehta U, Clerk C, Allen E, Yore M, Sevene E, Singlovic J, Petzold M, Mangiaterra V, Elefant E, Sullivan FM, Holmes LB, Gomes M. Protocol for a drugs exposure pregnancy registry for implementation in resource-limited settings. BMC Pregnancy Childbirth. 2012 Sep 3;12:89.



For peer review only

7. Clinicaltrials.gov. COVID-19 Vaccines International Pregnancy Exposure Registry (C-VIPER). https://clinicaltrials.gov/ct2/show/NCT04705116. Accessed October 25, 2022.

8. Vargesson N. Thalidomide-induced teratogenesis: History and mechanisms. Birth Defects Res C Embryo Today. 2015;105(2):140-156.

9. Engmann C, Fleming JA, Khan S, et al. Closer and closer? Maternal immunization: current promise, future horizons. J Perinatol. 2020;40(6):844-857. doi:10.1038/s41372-020-0668-3

10. Sobanjo-ter Meulen A, Munoz FM, Kaslow DC, Klugman KP, Omer SB, Vora P, Stergachis A. Maternal Interventions Vigilance Harmonization in Low- and Middle-Income Countries: Stakeholder Meeting Report; Amsterdam, May 1-2, 2018. Vaccine. 2019 May 6;37(20):2643-2650.

11. Gelperin K, Hammad H, Leishear K, Bird ST, Taylor L, Hampp C, Sahin L. A systematic review of pregnancy exposure registries: examination of protocol-specified pregnancy outcomes, target sample size, and comparator selection. Pharmacoepidemiol Drug Saf. 2017 Feb;26(2):208-214.

12. Berrueta M, Ciapponi A, Bardach A, Cairoli FR, Castellano FJ, Xiong X, Stergachis A, Zaraa S, Sobanjo-ter Meulen A, Buekens P and Scoping Review Collaboration Group. Maternal and neonatal data collection systems in low- and middle-income countries for maternal vaccines active safety surveillance systems: A scoping review. BMC Pregnancy and Childbirth. 2021 Mar 17;21:217.

13. Stuurman AL, Riera M, Lamprianou S, Perez-Vilar S, Anderson SA, Mangtani P, Devlieger H, Verstraeten T, Zuber PLF, Guillard Maure C. Vaccine safety surveillance in pregnancy in



For peer review only - http://bmjopen.bmj.com/site/about/guidelines.xhtml

low- and middle-income countries using GAIA case definitions: A feasibility assessment. Vaccine. 2018 Oct 29;36(45):6736-6743.

14. Gliklich RE, Dreyer NA, Leavy MB, editors. Registries for Evaluating Patient Outcomes: A User's Guide [Internet]. 3rd edition. Rockville (MD): Agency for Healthcare Research and Quality (US); 2014 Apr. 21, Pregnancy Registries. Available from: https://www.ncbi.nlm.nih.gov/books/NBK208605

15. Chapter 11: Scoping reviews – JBI Manual for Evidence Synthesis – JBI Global Wiki. Accessed March 7, 2022. https://jbi-global-wiki.refined.site/space/MANUAL/3283910770/Chapter+11%3A+Scoping+reviews.

16. McGowan J, Sampson M, Salzwedel DM, Cogo E, Foerster V, Lefebvre C. PRESS Peer Review of Electronic Search Strategies: 2015 Guideline Statement. J Clin Epidemiol. 2016;75:40-46.

17. Tricco AC, Lillie E, Zarin W, et al. PRISMA Extension for Scoping Reviews (PRISMA-ScR): Checklist and Explanation. Ann Intern Med. 2018;169(7):467-473.

18. OSF Registries. Accessed July 8, 2022. https://osf.io/registries.

19. World Bank Country and Lending Groups. World Bank. https://datahelpdesk.worldbank.org/knowledgebase/articles/906519-world-bank-country-and-lending-groups. Accessed July 8, 2022.

For peer review only



For peer review only - http://bmjopen.bmj.com/site/about/guidelines.xhtml

# Search Strategy by Database:

**PubMed**

| Set # | Query | Results |
|---|---|---|
| 132 | #130 NOT #131 | 2,016 |
| 131 | address[Publication Type] OR autobiography[Publication Type] OR bibliography[Publication Type] OR biography[Publication Type] OR comment[Publication Type] OR dictionary[Publication Type] OR directory[Publication Type] OR editorial[Publication Type] OR "expression of concern"[Publication Type] OR festschrift[Publication Type] OR historical article[Publication Type] OR interactive tutorial[Publication Type] OR lecture[Publication Type] OR news[Publication Type] OR newspaper article[Publication Type] OR portrait[Publication Type] OR video-audio media[Publication Type] OR webcast[Publication Type] | 2,050,653 |
| 130 | #128 NOT #129 | 2,048 |
| 129 | Animals[mesh] NOT Humans[mesh] | 5,016,862 |
| 128 | #126 AND #127 | 2,051 |
| 127 | ("2000"[Date - Publication] : "3000"[Date - Publication]) | 20,721,844 |
| 126 | #61 AND #125 | 2,597 |
| 125 | #62 OR #63 OR #64 OR #65 OR #66 OR #67 OR #68 OR #69 OR #70 OR #71 OR #72 OR #73 OR #74 OR #75 OR #76 OR #77 OR #78 OR #79 OR #80 OR #81 OR #82 OR #83 OR #84 OR #85 OR #86 OR #87 OR #88 OR #89 OR #90 OR #91 OR #92 OR #93 OR #94 OR #95 OR #96 OR #97 OR #98 OR #99 OR #100 OR #101 OR #102 OR #103 OR #104 OR #105 OR #106 OR #107 OR #108 OR #109 OR #110 OR #111 OR #112 OR #113 OR #114 OR #115 OR #116 OR #117 OR #118 OR #119 OR #120 OR #121 OR #122 OR #123 OR #124 | 7,704,889 |
| 124 | MNCH[Other Term] AND (maternal*[Other Term] OR newborn*[Other Term] OR child*[Other Term]) | 11 |
| 123 | MNCH[Title] AND (maternal*[Title] OR newborn*[Title] OR child*[Title]) | 22 |
| 122 | "Maternal Health"[mesh] | 2,142 |
| 121 | "Child Health"[mesh] | 4,447 |
| 120 | "Infant Health"[mesh] | 1,175 |
| 119 | (birth[Other Term] OR births[Other Term]) AND defect*[Other Term] | 1,029 |
| 118 | (birth[Title] OR births[Title]) AND defect*[Title] | 2,671 |

For peer review only

| | | |
|---|---|---|
| 117 | (birth[Other Term] OR births[Other Term] OR matern*[Other Term] OR neonat*[Other Term] OR neo-nat*[Other Term] OR perinatal*[Other Term] OR peri-natal*[Other Term] OR peripartum[Other Term] OR "peri-partum"[Other Term] OR postnatal*[Other Term] OR post-natal*[Other Term] OR postpartum[Other Term] OR "post-partum"[Other Term] OR post-birth*[Other Term] OR pregnanc*[Other Term]) AND outcome*[Other Term] | 9,426 |
| 116 | (birth[Title] OR births[Title] OR matern*[Title] OR neonat*[Title] OR neo-nat*[Title] OR perinatal*[Title] OR peri-natal*[Title] OR peripartum[Title] OR "peri-partum"[Title] OR postnatal*[Title] OR post-natal*[Title] OR postpartum[Title] OR "post-partum"[Title] OR post-birth*[Title] OR pregnanc*[Title]) AND outcome*[Title] | 31,066 |
| 115 | Pregnancy Outcome[mesh] | 82,242 |
| 114 | (fetal[OT] OR foetal[OT] OR fetus*[OT] OR foetus*[OT] OR prenatal*[OT] OR pre-natal*[OT]) AND expos*[OT] | 2,033 |
| 113 | (fetal[Title] OR foetal[Title] OR fetus*[Title] OR foetus*[Title] OR prenatal*[Title] OR pre-natal*[Title]) AND expos*[Title] | 10,858 |
| 112 | "Prenatal Exposure Delayed Effects"[mesh] | 33,016 |
| 111 | (fetal[Other Term] OR foetal[Other Term] OR fetus*[Other Term] OR foetus*[Other Term]) AND develop*[Other Term] | 2,531 |
| 110 | (fetal[Title] OR foetal[Title] OR fetus*[Title] OR foetus*[Title]) AND develop*[Title] | 8,734 |
| 109 | "Fetal Development"[mesh:noexp] | 9,246 |
| 108 | antiretroviral*[Other Term] OR anti-retroviral*[Other Term] OR "anti-HIV"[Other Term] OR "anti-AIDS"[Other Term] OR "AIDS drug"[Other Term] OR "AIDS drugs"[Other Term] | 7,307 |
| 107 | (antiretroviral*[Title] OR anti-retroviral*[Title] OR "anti-HIV"[Title] OR "anti-AIDS"[Title] OR "AIDS drug"[Title] OR "AIDS drugs"[Title]) | 29,826 |
| 106 | "Anti-Retroviral Agents"[mesh] | 69,532 |
| 105 | "Zika Virus Infection/prevention and control"[MeSH] | 1,096 |
| 104 | "Streptococcal Infections/prevention and control"[MeSH:noexp] | 2,944 |
| 103 | "Respiratory Syncytial Virus Infections/prevention and control"[MeSH Terms] | 1,832 |
| 102 | "Hepatitis E/prevention and control"[MeSH Terms] | 346 |
| 101 | "Cytomegalovirus Infections/prevention and control"[MeSH Terms] | 3,130 |
| 100 | antimalarial*[Other Term] OR anti-malarial*[Other Term] | 2,577 |
| 99 | antimalarial*[Title] OR anti-malarial*[Title] | 8,562 |
| 98 | Antimalarials[mesh] | 28,226 |

For peer review only

| | | |
|---|---|---|
| 97 | "Malaria/prevention and control"[MeSH Terms] | 17,528 |
| 96 | (maternal[Other Term] OR pregnan*[Other Term]) AND (immunisation*[Other Term] OR immunization*[Other Term]) | 754 |
| 95 | (maternal[Title] OR pregnan*[Title]) AND (immunisation*[Title] OR immunization*[Title]) | 1,153 |
| 94 | (antibod*[Other Term] OR anti-bod*[Other Term]) AND transfer*[Other Term] | 338 |
| 93 | (antibod*[Title] OR anti-bod*[Title]) AND transfer*[Title] | 1,620 |
| 92 | immunit*[Other Term] AND maternally-acqui*[Other Term] | 8 |
| 91 | immunit*[Title] AND maternally-acqui*[Title] | 8 |
| 90 | immunit*[Other Term] AND transfer*[Other Term] | 161 |
| 89 | immunit*[Title] AND transfer*[Title] | 882 |
| 88 | "Immunity, Maternally-Acquired"[mesh] | 5,842 |
| 87 | "Immunization Programs"[mesh:noexp] | 12,435 |
| 86 | Immunization[mesh:noexp] | 144,668 |
| 85 | vaccin*[ti] OR vaccin*[ot] | 220,223 |
| 84 | "Viral Vaccines"[mesh] | 132,729 |
| 83 | "Vaccines, Live, Unattenuated"[mesh] | 82 |
| 82 | "Vaccines, Synthetic"[mesh] | 31,589 |
| 81 | "Vaccines, Inactivated"[mesh:noexp] | 6,082 |
| 80 | "Vaccines, Combined"[mesh] | 10,562 |
| 79 | "Vaccines, Attenuated"[mesh] | 12,810 |
| 78 | "Protozoan Vaccines"[mesh] | 6,894 |
| 77 | "Bacterial Vaccines"[mesh] | 72,365 |
| 76 | Vaccines[mesh:noexp] | 25,746 |
| 75 | Vaccination[mesh] | 100,718 |
| 74 | GAIA[Text Word] AND (alignment[Text Word] OR immunisation[Text Word] OR immunization[Text Word] OR safety[Text Word] OR pregnan*[Text Word]) | 35 |
| 73 | "Global Alignment of Immunization Safety Assessment in Pregnancy"[tw] - Schema: all | 0 |
| 72 | AEFI[Other Term] AND adverse[Other Term] | 68 |
| 71 | AEFI[Title] AND adverse[Title] | 35 |
| 70 | adverse effect[Other Term] OR adverse effects[Other Term] OR adverse reaction[Other Term] OR adverse reactions[Other Term] OR adverse event[Other Term] OR adverse events[Other Term] OR adverse outcome[Other Term] OR adverse outcomes[Other Term] | 17,729 |
| 69 | adverse effect[Title] OR adverse effects[Title] OR adverse reaction[Title] OR adverse reactions[Title] OR adverse event[Title] OR adverse events[Title] OR adverse outcome[Title] OR adverse outcomes[Title] | 31,848 |

For peer review only

| | | |
|---|---|---|
| 68 | safe[Other Term] OR safety[Other Term] OR side effect[Other Term] OR side effects[Other Term] OR undesirable effect[Other Term] OR undesirable effects[Other Term] OR treatment emergent[Other Term] OR tolerability[Other Term] OR toxicity[Other Term] OR adrs[Other Term] | 98,171 |
| 67 | safe[Title] OR safety[Title] OR side effect[Title] OR side effects[Title] OR undesirable effect[Title] OR undesirable effects[Title] OR treatment emergent[Title] OR tolerability[Title] OR toxicity[Title] OR adrs[Title] | 296,710 |
| 66 | ("adverse effects"[MeSH Subheading]) OR (Complications[MeSH Subheading]) OR ("drug effects"[MeSH Subheading]) | 6,963,724 |
| 65 | (drug[Title] OR drugs[Title] OR medicine[Title] OR medicines[Title] OR medication*[Title] OR pharmaceutical*[Title] OR pharma-ceutical*[Title]) AND expos*[Title] | 4,387 |
| 64 | "Drug-Related Side Effects and Adverse Reactions"[MeSH Terms] | 127,437 |
| 63 | (global[Title] OR international*[Title] OR world*[Title]) AND health*[Title] | 27,778 |
| 62 | Global Health[mesh] | 53,845 |
| 61 | #56 AND #60 | 8,450 |
| 60 | #57 OR #58 OR #59 | 1,104,126 |
| 59 | "high burden country"[Other Term] OR "high burden countries"[Other Term] OR "high-burden country"[Other Term] OR "high-burden countries"[Other Term] OR "countdown country"[Other Term] OR "countdown countries"[Other Term] | 10 |
| 58 | "high burden country"[Title/Abstract] OR "high burden countries"[Title/Abstract] OR "high-burden country"[Title/Abstract] OR "high-burden countries"[Title/Abstract] OR "countdown country"[Title/Abstract] OR "countdown countries"[Title/Abstract] | 633 |

For peer review only

| | | |
|---|---|---|
| 57 | afghanistan[Text Word] OR albania[Text Word] OR algeria[Text Word] OR "american samoa"[Text Word] OR angola[Text Word] OR antigua[Text Word] OR barbuda[Text Word] OR argentina[Text Word] OR armenia[Text Word] OR armenian[Text Word] OR aruba[Text Word] OR azerbaijan[Text Word] OR bahrain[Text Word] OR bangladesh[Text Word] OR barbados[Text Word] OR belarus[Text Word] OR byelarus[Text Word] OR belorussia[Text Word] OR byelorussian[Text Word] OR belize[Text Word] OR "british honduras"[Text Word] OR benin[Text Word] OR dahomey[Text Word] OR bhutan[Text Word] OR bolivia[Text Word] OR "bosnia herzegovina"[Text Word] OR bosnia[Text Word] OR herzegovina[Text Word] OR botswana[Text Word] OR bechuanaland[Text Word] OR brazil[Text Word] OR brasil[Text Word] OR bulgaria[Text Word] OR "burkina faso"[Text Word] OR "burkina fasso"[Text Word] OR "upper volta"[Text Word] OR burundi[Text Word] OR urundi[Text Word] OR "cabo verde"[Text Word] OR "cape verde"[Text Word] OR cambodia[Text Word] OR kampuchea[Text Word] OR "khmer republic"[Text Word] OR cameroon[Text Word] OR cameron[Text Word] OR cameroun[Text Word] OR "central african republic"[Text Word] OR "ubangi shari"[Text Word] OR chad[Text Word] OR chile[Text Word] OR china[Text Word] OR colombia[Text Word] OR comoros[Text Word] OR "comoro islands"[Text Word] OR "iles comores"[Text Word] OR mayotte[Text Word] OR "democratic republic of the congo"[Text Word] OR "democratic republic congo"[Text Word] OR congo[Text Word] OR zaire[Text Word] OR "costa rica"[Text Word] OR "cote d'ivoire"[Text Word] OR "cote d' ivoire"[Text Word] OR "cote divoire"[Text Word] OR "cote d ivoire"[Text Word] OR "ivory coast"[Text Word] OR croatia[Text Word] OR cuba[Text Word] OR cyprus[Text Word] OR "czech republic"[Text Word] OR czechoslovakia[Text Word] OR djibouti[Text Word] OR "french somaliland"[Text Word] OR dominica[Text Word] OR "dominican republic"[Text Word] OR ecuador[Text Word] OR egypt[Text Word] OR "united arab republic"[Text Word] OR "el salvador"[Text Word] OR "equatorial guinea"[Text Word] OR "spanish guinea"[Text Word] OR eritrea[Text Word] OR estonia[Text Word] OR eswatini[Text Word] OR swaziland[Text Word] OR ethiopia[Text Word] OR fiji[Text Word] OR gabon[Text Word] OR "gabonese republic"[Text Word] OR gambia[Text Word] OR "georgia (republic)"[Text Word] OR georgian[Text Word] OR ghana[Text Word] OR "gold coast"[Text Word] OR gibraltar[Text Word] OR greece[Text Word] OR grenada[Text Word] OR guam[Text Word] OR guatemala[Text Word] OR guinea[Text Word] OR "guinea bissau"[Text Word] OR guyana[Text Word] OR "british guiana"[Text Word] OR haiti[Text Word] OR hispaniola[Text Word] OR honduras[Text Word] OR hungary[Text Word] OR india[Text Word] OR indonesia[Text Word] OR timor[Text Word] OR iran[Text Word] OR iraq[Text Word] OR "isle | 1,103,822 |

For peer review only

of man"[Text Word] OR jamaica[Text Word] OR jordan[Text Word] OR kazakhstan[Text Word] OR kazakh[Text Word] OR kenya[Text Word] OR "democratic people's republic of korea"[Text Word] OR "republic of korea"[Text Word] OR "north korea"[Text Word] OR "south korea"[Text Word] OR korea[Text Word] OR kosovo[Text Word] OR kyrgyzstan[Text Word] OR kirghizia[Text Word] OR kirgizstan[Text Word] OR "kyrgyz republic"[Text Word] OR kirghiz[Text Word] OR laos[Text Word] OR "lao pdr"[Text Word] OR "lao people's democratic republic"[Text Word] OR latvia[Text Word] OR lebanon[Text Word] OR lebanese republic[Text Word] OR lesotho[Text Word] OR basutoland[Text Word] OR liberia[Text Word] OR libya[Text Word] OR "libyan arab jamahiriya"[Text Word] OR lithuania[Text Word] OR macau[Text Word] OR macao[Text Word] OR republic of "north macedonia"[Text Word] OR macedonia[Text Word] OR madagascar[Text Word] OR "malagasy republic"[Text Word] OR malawi[Text Word] OR nyasaland[Text Word] OR malaysia[Text Word] OR "malay federation"[Text Word] OR "malaya federation"[Text Word] OR maldives[Text Word] OR "indian ocean islands"[Text Word] OR "indian ocean"[Text Word] OR mali[Text Word] OR malta[Text Word] OR micronesia[Text Word] OR "federated states of micronesia"[Text Word] OR kiribati[Text Word] OR "marshall islands"[Text Word] OR nauru[Text Word] OR "northern mariana islands"[Text Word] OR palau[Text Word] OR tuvalu[Text Word] OR mauritania[Text Word] OR mauritius[Text Word] OR mexico[Text Word] OR moldova[Text Word] OR moldovian[Text Word] OR mongolia[Text Word] OR montenegro[Text Word] OR morocco[Text Word] OR ifni[Text Word] OR mozambique[Text Word] OR "portuguese east africa"[Text Word] OR myanmar[Text Word] OR burma[Text Word] OR namibia[Text Word] OR nepal[Text Word] OR "netherlands antilles"[Text Word] OR nicaragua[Text Word] OR niger[Text Word] OR nigeria[Text Word] OR oman[Text Word] OR muscat[Text Word] OR pakistan[Text Word] OR panama[Text Word] OR "papua new guinea"[Text Word] OR "new guinea"[Text Word] OR paraguay[Text Word] OR peru[Text Word] OR philippines[Text Word] OR philipines[Text Word] OR phillipines[Text Word] OR phillippines[Text Word] OR poland[Text Word] OR "polish people's republic"[Text Word] OR portugal[Text Word] OR "portuguese republic"[Text Word] OR "puerto rico"[Text Word] OR romania[Text Word] OR russia[Text Word] OR "russian federation"[Text Word] OR ussr[Text Word] OR "soviet union"[Text Word] OR "union of soviet socialist republics"[Text Word] OR rwanda[Text Word] OR ruanda[Text Word] OR samoa[Text Word] OR "pacific islands"[Text Word] OR polynesia[Text Word] OR "samoan islands"[Text Word] OR "navigator island"[Text Word] OR "navigator islands"[Text Word] OR "sao tome and principe"[Text Word] OR "saudi arabia"[Text Word] OR senegal[Text Word] OR serbia[Text Word] OR

For peer review only

seychelles[Text Word] OR "sierra leone"[Text Word] OR slovakia[Text Word] OR "slovak republic"[Text Word] OR slovenia[Text Word] OR melanesia[Text Word] OR "solomon island"[Text Word] OR "solomon islands"[Text Word] OR "norfolk island"[Text Word] OR "norfolk islands"[Text Word] OR somalia[Text Word] OR "south africa"[Text Word] OR "south sudan"[Text Word] OR "sri lanka"[Text Word] OR ceylon[Text Word] OR "saint kitts and nevis"[Text Word] OR "st. kitts and nevis"[Text Word] OR "saint lucia"[Text Word] OR "st. lucia"[Text Word] OR "saint Vincent and the grenadines"[Text Word] OR "saint vincent"[Text Word] OR "st. vincent"[Text Word] OR grenadines[Text Word] OR sudan[Text Word] OR suriname[Text Word] OR surinam[Text Word] OR "dutch guiana"[Text Word] OR "netherlands guiana"[Text Word] OR syria[Text Word] OR "syrian arab republic"[Text Word] OR tajikistan[Text Word] OR tadjikistan[Text Word] OR tadzhikistan[Text Word] OR tadzhik[Text Word] OR tanzania[Text Word] OR tanganyika[Text Word] OR thailand[Text Word] OR siam[Text Word] OR "timor leste"[Text Word] OR "east timor"[Text Word] OR togo[Text Word] OR "togolese republic"[Text Word] OR tonga[Text Word] OR "Trinidad and tobago"[Text Word] OR trinidad[Text Word] OR tobago[Text Word] OR tunisia[Text Word] OR turkey[Text Word] OR turkmenistan[Text Word] OR turkmen[Text Word] OR uganda[Text Word] OR ukraine[Text Word] OR uruguay[Text Word] OR uzbekistan[Text Word] OR uzbek[Text Word] OR vanuatu[Text Word] OR "new hebrides"[Text Word] OR venezuela[Text Word] OR vietnam[Text Word] OR "viet nam"[Text Word] OR "middle east"[Text Word] OR "west bank"[Text Word] OR gaza[Text Word] OR palestine[Text Word] OR yemen[Text Word] OR yugoslavia[Text Word] OR zambia[Text Word] OR zimbabwe[Text Word] OR "northern rhodesia"[Text Word] OR "global south"[Text Word] OR "africa south of the sahara"[Text Word] OR "sub-saharan africa"[Text Word] OR "subsaharan africa"[Text Word] OR "africa, central"[Text Word] OR "central africa"[Text Word] OR "africa, northern"[Text Word] OR "north africa"[Text Word] OR "northern africa"[Text Word] OR magreb[Text Word] OR maghrib[Text Word] OR sahara[Text Word] OR "africa, southern"[Text Word] OR "southern africa"[Text Word] OR "africa, eastern"[Text Word] OR "east africa"[Text Word] OR "eastern africa"[Text Word] OR "africa, western"[Text Word] OR "west africa"[Text Word] OR "western africa"[Text Word] OR "west indies"[Text Word] OR caribbean[Text Word] OR "central america"[Text Word] OR "latin america"[Text Word] OR "south and central america"[Text Word] OR "south america"[Text Word] OR "asia, central"[Text Word] OR "central asia"[Text Word] OR "asia, northern"[Text Word] OR "north asia"[Text Word] OR "northern asia"[Text Word] OR "asia, southeastern"[Text Word] OR "southeastern asia"[Text Word] OR

For peer review only

"south eastern asia"[Text Word] OR "southeast asia"[Text Word] OR "south east asia"[Text Word] OR "asia, western"[Text Word] OR "western asia"[Text Word] OR "europe, eastern"[Text Word] OR "east europe"[Text Word] OR "eastern europe"[Text Word] OR "developing country"[Text Word] OR "developing countries"[Text Word] OR "developing nation"[Text Word] OR "developing nations"[Text Word] OR "developing population"[Text Word] OR "developing populations"[Text Word] OR "developing world"[Text Word] OR "less developed country"[Text Word] OR "less developed countries"[Text Word] OR "less developed nation"[Text Word] OR "less developed nations"[Text Word] OR "less developed population"[Text Word] OR "less developed populations"[Text Word] OR "less developed world"[Text Word] OR "lesser developed country"[Text Word] OR "lesser developed countries"[Text Word] OR "lesser developed nation"[Text Word] OR "lesser developed nations"[Text Word] OR "lesser developed population"[Text Word] OR "lesser developed populations"[Text Word] OR "lesser developed world"[Text Word] OR "under developed country"[Text Word] OR "under developed countries"[Text Word] OR "under developed nation"[Text Word] OR "under developed nations"[Text Word] OR "under developed population"[Text Word] OR "under developed populations"[Text Word] OR "under developed world"[Text Word] OR "underdeveloped country"[Text Word] OR "underdeveloped countries"[Text Word] OR "underdeveloped nation"[Text Word] OR "underdeveloped nations"[Text Word] OR "underdeveloped population"[Text Word] OR "underdeveloped populations"[Text Word] OR "underdeveloped world"[Text Word] OR "middle income country"[Text Word] OR "middle income countries"[Text Word] OR "middle income nation"[Text Word] OR "middle income nations"[Text Word] OR "middle income population"[Text Word] OR "middle income populations"[Text Word] OR "low income country"[Text Word] OR "low income countries"[Text Word] OR "low income nation"[Text Word] OR "low income nations"[Text Word] OR "low income population"[Text Word] OR "low income populations"[Text Word] OR "lower income country"[Text Word] OR "lower income countries"[Text Word] OR "lower income nation"[Text Word] OR "lower income nations"[Text Word] OR "lower income population"[Text Word] OR "lower income populations"[Text Word] OR "underserved country"[Text Word] OR "underserved countries"[Text Word] OR "underserved nation"[Text Word] OR "underserved nations"[Text Word] OR "underserved population"[Text Word] OR "underserved populations"[Text Word] OR "underserved world"[Text Word] OR "under served country"[Text Word] OR "under served countries"[Text Word] OR "under served nation"[Text Word] OR "under served nations"[Text Word] OR "under served population"[Text Word] OR "under served populations"[Text

For peer review only

| | | |
|---|---|---|
| | Word] OR "under served world"[Text Word] OR "deprived country"[Text Word] OR "deprived countries"[Text Word] OR "deprived nation"[Text Word] OR "deprived nations"[Text Word] OR "deprived population"[Text Word] OR "deprived populations"[Text Word] OR "deprived world"[Text Word] OR "poor country"[Text Word] OR "poor countries"[Text Word] OR "poor nation"[Text Word] OR "poor nations"[Text Word] OR "poor population"[Text Word] OR "poor populations"[Text Word] OR "poor world"[Text Word] OR "poorer country"[Text Word] OR "poorer countries"[Text Word] OR "poorer nation"[Text Word] OR "poorer nations"[Text Word] OR "poorer population"[Text Word] OR "poorer populations"[Text Word] OR "poorer world"[Text Word] OR "developing economy"[Text Word] OR "developing economies"[Text Word] OR "less developed economy"[Text Word] OR "less developed economics"[Text Word] OR "lesser developed economy"[Text Word] OR "lesser developed economies"[Text Word] OR "under developed economy"[Text Word] OR "under developed economies"[Text Word] OR "underdeveloped economy"[Text Word] OR "underdeveloped economies"[Text Word] OR "middle income economy"[Text Word] OR "middle income economies"[Text Word] OR "low income economy"[Text Word] OR "low income economies"[Text Word] OR "lower income economy"[Text Word] OR "lower income economies"[Text Word] OR "low gdp"[Text Word] OR "low gnp"[Text Word] OR "low gross domestic"[Text Word] OR "low gross national"[Text Word] OR "lower gdp"[Text Word] OR "lower gnp"[Text Word] OR "lower gross domestic"[Text Word] OR "lower gross national"[Text Word] OR lmic[Text Word] OR lmics[Text Word] OR "third world"[Text Word] OR "lami country"[Text Word] OR "lami countries"[Text Word] OR "transitional country"[Text Word] OR "transitional economies"[Text Word] OR "emerging economy"[Text Word] OR "emerging economies"[Text Word] OR "emerging nation"[Text Word] OR "emerging nations"[Text Word] | |
| 56 | #1 OR #55 | 39,866 |
| 55 | #21 AND #54 | 39,859 |
| 54 | #22 OR #23 OR #24 OR #25 OR #26 OR #27 OR #28 OR #29 OR #30 OR #31 OR #32 OR #33 OR #34 OR #35 OR #36 OR #37 OR #38 OR #39 OR #40 OR #41 OR #42 OR #43 OR #44 OR #45 OR #46 OR #47 OR #48 OR #49 OR #50 OR #51 OR #52 OR #53 | 850,027 |
| 53 | "Product Surveillance, Postmarketing/statistics and numerical data"[MeSH Terms] | 2,361 |
| 52 | "Maternal Health Services/statistics and numerical data"[MeSH Terms] | 7,290 |
| 51 | "Maternal Exposure/statistics and numerical data"[MeSH Terms] | 1,123 |

For peer review only

| | | |
|---|---|---|
| 50 | pharmacovigilan*[ti] OR pharmaco-vigilan*[ti] OR pharmacovigilan*[ot] OR pharmaco-vigilan*[ot] | 3,751 |
| 49 | "Product Surveillance, Postmarketing"[mesh:noexp] | 7,553 |
| 48 | "data system"[ti] OR "data systems"[ti] OR "information system"[ti] OR "information systems"[ti] OR "data system"[ot] OR "data systems"[ot] OR "information system"[ot] OR "information systems"[ot] | 13,581 |
| 47 | "Health Information Systems"[mesh] | 1,546 |
| 46 | (decision*[ti] AND support*[ti] AND clinical*[ti]) OR (decision*[ot] AND support*[ot] AND clinical*[ot]) | 4,467 |
| 45 | "Decision Support Systems, Clinical"[mesh] | 9,106 |
| 44 | "Databases, Factual"[mesh] | 163,862 |
| 43 | "Databases as Topic"[mesh:noexp] | 9,688 |
| 42 | surveillance*[ti] OR surveillance*[ot] | 61,304 |
| 41 | "Population Surveillance"[mesh] | 74,026 |
| 40 | survey*[ti] OR survey*[ot] | 189,840 |
| 39 | "Health Surveys"[mesh:noexp] | 66,266 |
| 38 | "Health Care Surveys"[MeSH Major Topic] | 10,167 |
| 37 | "Surveys and Questionnaires"[MeSH Major Topic:noexp] | 49,876 |
| 36 | registry[ot] OR registries[ot] OR eregistr*[ot] OR "e-registry"[ot] OR "e-registries"[ot] | 8,509 |
| 35 | registry[ti] OR registries[ti] OR eregistr*[ti] OR "e-registry"[ti] OR "e-registries"[ti] | 34,311 |
| 34 | Registries[mesh:noexp] | 104,749 |
| 33 | (preliminary[ot] OR "pilot project"[ot] OR "pilot projects"[ot]) AND data[ot] | 35 |
| 32 | (preliminary[ti] OR "pilot project"[ti] OR "pilot projects"[ti]) AND data[ti] | 2,947 |
| 31 | "Preliminary Data"[mesh] | 724 |
| 30 | "focus group"[ti] OR "focus groups"[ti] OR "focus group"[ot] OR "focus groups"[ot] | 6,048 |
| 29 | "Focus Groups"[mesh] | 34,384 |
| 28 | databas*[ot] OR "data base"[ot] OR "data bases"[ot] OR databank*[ot] OR "data bank"[ot] OR "data banks"[ot] OR dataset*[ot] OR "data set"[ot] OR "data sets"[ot] | 14,502 |
| 27 | databas*[ti] OR "data base"[ti] OR "data bases"[ti] OR databank*[ti] OR "data bank"[ti] OR "data banks"[ti] OR dataset*[ti] OR "data set"[ti] OR "data sets"[ti] | 55,119 |
| 26 | "Datasets as Topic"[mesh] | 7,252 |
| 25 | data[ot] AND (accumulat*[ot] OR accura*[ot] OR assembl*[ot] OR captur*[ot] OR collect*[ot] OR compil*[ot] OR coordinat*[ot] OR co-ordinat*[ot] OR gather*[ot] OR hub[ot] OR hubs[ot]) | 5,205 |

For peer review only

| | | |
|---|---|---|
| 24 | data[ti] AND (accumulat*[ti] OR accura*[ti] OR assembl*[ti] OR captur*[ti] OR collect*[ti] OR compil*[ti] OR coordinat*[ti] OR co-ordinat*[ti] OR gather*[ti] OR hub[ti] OR hubs[ti]) | 11,365 |
| 23 | "Data Accuracy"[mesh] | 3,726 |
| 22 | "Data Collection"[mesh:noexp] | 91,761 |
| 21 | #2 OR #3 OR #4 OR #5 OR #6 OR #7 OR #8 OR #9 OR #10 OR #11 OR #12 OR #13 OR #14 OR #15 OR #16 OR #17 OR #18 OR #19 OR #20 | 1,067,642 |
| 20 | maternal[ot] AND (fetal[ot] OR fetus[ot] OR foetal[ot] OR foetus[ot]) | 3,848 |
| 19 | (maternal*[ot] OR maternity[ot]) AND (care[ot] OR health*[ot] OR service[ot] OR services[ot]) | 11,210 |
| 18 | (maternal*[ot] or maternity[ot]) AND (contact*[ot] or expos*[ot]) | 601 |
| 17 | perinatal*[ot] OR "peri-natal"[ot] OR "peri-natally"[ot] OR peripartum[ot] or "peri-partum"[ot] | 7,552 |
| 16 | prenatal*[ot] OR antenatal*[ot] OR "ante-natal"[ot] OR "ante-natally"[ot] OR antepartum[ot] or "ante-partum"[ot] | 15,862 |
| 15 | maternal[ti] AND (fetal[ti] OR fetus[ti] OR foetal[ti] OR foetus[ti]) | 13,964 |
| 14 | (maternal*[ti] OR maternity[ti]) AND (care[ti] OR health*[ti] OR service[ti] OR services[ti]) | 13,467 |
| 13 | "Maternal Health Services"[mesh:noexp] | 15,617 |
| 12 | (maternal*[ti] or maternity[ti]) AND (contact*[ti] or expos*[ti]) | 3,847 |
| 11 | "Maternal Exposure"[mesh] | 10,689 |
| 10 | perinatal*[ti] OR "peri-natal"[ti] OR "peri-natally"[ti] OR peripartum[ti] or "peri-partum"[ti] | 30,982 |
| 9 | "Perinatal Care"[mesh] | 11,334 |
| 8 | prenatal*[ti] OR antenatal*[ti] OR "ante-natal"[ti] OR "ante-natally"[ti] OR antepartum[ti] or "ante-partum"[ti] | 59,414 |
| 7 | "Prenatal Care"[mesh] | 31,249 |
| 6 | pregnan*[ti] | 252,201 |
| 5 | "Pregnancy Trimesters"[mesh] | 43,874 |
| 4 | "Pregnant Women"[mesh] | 12,334 |
| 3 | "Pregnancy Complications"[mesh] | 460,551 |
| 2 | Pregnancy[mesh] | 970,546 |
| 1 | "pregnancy exposure database" [tw] OR "pregnancy exposure databases" [tw] OR "pregnancy exposure data base" [tw] OR "pregnancy exposure data bases" [tw] OR "pregnancy exposure registry" [tw] OR "pregnancy exposure registries" [tw] | 34 |

For peer review only

**Embase**

--------------------------------------------------------------------------------
1 ("pregnancy exposure database" or "pregnancy exposure databases" or "pregnancy exposure data base" or "pregnancy exposure data bases" or "pregnancy exposure registry" or "pregnancy exposure registries").tw,kw,kf. (113)
2 exp pregnancy/ (727815)
3 exp pregnancy complication/ (141579)
4 exp named groups by pregnancy/ (130431)
5 pregnan*.ti,kw,kf. (329712)
6 exp prenatal care/ (167165)
7 (prenatal* or antenatal* or "ante-natal" or "ante-natally" or antepartum or "ante-partum").ti,kw,kf. (87488)
8 exp perinatal care/ (65235)
9 (perinatal* or peri-natal* or peripartum or "peri-partum").ti,kw,kf. (46195)
10 maternal exposure/ (3606)
11 ((maternal* or maternity) and (contact* or expos*)).ti,kw,kf. (6357)
12 maternal health service/ (2454)
13 ((maternal* or maternity) and (care or health* or service or services)).ti,kw,kf. (22738)
14 (maternal and (fetal or fetus or foetal or foetus)).ti,kw,kf. (20741)
15 or/2-14 [PREGNANCY] (1035932)
16 *information processing/ (37078)
17 data aggregation/ (374)
18 data accuracy/ (1596)
19 (data and (accumulat* or accura* or assembl* or captur* or collect* or compil* or coordinat* or co-ordinat* or gather* or hub or hubs)).ti,kw,kf. (18259)
20 (databas* or "data base" or "data bases" or databank* or "data bank" or "data banks" or dataset* or "data set" or "data sets").ti,kw,kf. (86633)
21 (focus group or focus groups).ti,kw,kf. (7608)
22 preliminary data/ (31472)
23 ((preliminary or pilot project or pilot projects) and data).ti,kw,kf. (5481)
24 exp register/ (180900)
25 (registry or registries or eregistr* or "e-registry" or "e-registries").ti,kw,kf. (68347)
26 questionnaire/ (814172)
27 *health care survey/ (2606)
28 health survey/ (213987)
29 survey*.ti,kw,kf. (220889)
30 population surveillance/ (117)
31 surveillance*.ti,kw,kf. (82221)
32 data base/ (248533)
33 factual database/ (27912)
34 clinical decision support system/ (4716)
35 (decision* and support* and clinical*).ti,kw,kf. (5904)
36 medical information system/ (22591)
37 ("data system" or "data systems" or "information system" or "information systems").ti,kw,kf. (18222)
38 exp postmarketing surveillance/ (37924)
39 (pharmacovigilan* or pharmaco-vigilan*).ti,kw,kf. (7417)

For peer review only

40 or/16-39 [DATA COLLECTION, REGISTRIES] (1815231)
41 15 and 40 [PREGNANCY - DATA COLLECTION, REGISTRIES] (71373)
42 1 or 41 [PERs, PREGNANCY - DATA COLLECTION, REGISTRIES] (71381)
43 (afghanistan or albania or algeria or american samoa or angola or "antigua and barbuda" or antigua or barbuda or argentina or armenia or armenian or aruba or azerbaijan or bahrain or bangladesh or barbados or republic of belarus or belarus or byelarus or belorussia or byelorussian or belize or british honduras or benin or dahomey or bhutan or bolivia or "bosnia and herzegovina" or bosnia or herzegovina or botswana or bechuanaland or brazil or brasil or bulgaria or burkina faso or burkina fasso or upper volta or burundi or urundi or cabo verde or cape verde or cambodia or kampuchea or khmer republic or cameroon or cameron or cameroun or central african republic or ubangi shari or chad or chile or china or colombia or comoros or comoro islands or iles comores or mayotte or democratic republic of the congo or democratic republic congo or congo or zaire or costa rica or "cote d'ivoire" or "cote d' ivoire" or cote divoire or cote d ivoire or ivory coast or croatia or cuba or cyprus or czech republic or czechoslovakia or djibouti or french somaliland or dominica or dominican republic or ecuador or egypt or united arab republic or el salvador or equatorial guinea or spanish guinea or eritrea or estonia or eswatini or swaziland or ethiopia or fiji or gabon or gabonese republic or gambia or "georgia (republic)" or georgian or ghana or gold coast or gibraltar or greece or grenada or guam or guatemala or guinea or guinea bissau or guyana or british guiana or haiti or hispaniola or honduras or hungary or india or indonesia or timor or iran or iraq or isle of man or jamaica or jordan or kazakhstan or kazakh or kenya or "democratic people's republic of korea" or republic of korea or north korea or south korea or korea or kosovo or kyrgyzstan or kirghizia or kirgizstan or kyrgyz republic or kirghiz or laos or lao pdr or "lao people's democratic republic" or latvia or lebanon or lebanese republic or lesotho or basutoland or liberia or libya or libyan arab jamahiriya or lithuania or macau or macao or republic of north macedonia or macedonia or madagascar or malagasy republic or malawi or nyasaland or malaysia or malay federation or malaya federation or maldives or indian ocean islands or indian ocean or mali or malta or micronesia or federated states of micronesia or kiribati or marshall islands or nauru or northern mariana islands or palau or tuvalu or mauritania or mauritius or mexico or moldova or moldovian or mongolia or montenegro or "montenegro (republic)" or morocco or ifni or mozambique or portuguese east africa or myanmar or burma or namibia or nepal or netherlands antilles or nicaragua or niger or nigeria or oman or muscat or pakistan or panama or papua new guinea or new guinea or paraguay or peru or philippines or philipines or phillipines or phillippines or poland or "polish people's republic" or portugal or portuguese republic or puerto rico or romania or russia or russian federation or ussr or soviet union or union of soviet socialist republics or rwanda or ruanda or samoa or pacific islands or polynesia or samoan islands or navigator island or navigator islands or "sao tome and principe" or saudi arabia or senegal or serbia or seychelles or sierra leone or slovakia or slovak republic or slovenia or melanesia or solomon island or solomon islands or norfolk island or norfolk islands or somalia or south africa or south sudan or sri lanka or ceylon or "saint kitts and nevis" or "st. kitts and nevis" or saint lucia or "st. lucia" or "saint vincent and the grenadines" or saint vincent or "st. vincent" or grenadines or sudan or suriname or surinam or dutch guiana or netherlands guiana or syria or syrian arab republic or tajikistan or tadjikistan or tadzhikistan or tadzhik or tanzania or tanganyika or thailand or siam or timor leste or east timor or togo or togolese republic or tonga or "trinidad and tobago" or trinidad or tobago or tunisia or "turkey (republic)" or turkey or turkmenistan or turkmen or uganda or ukraine or uruguay or uzbekistan or uzbek or vanuatu or new hebrides or venezuela or vietnam or viet nam or middle east or west bank or gaza or palestine or yemen or yugoslavia or zambia or zimbabwe or northern rhodesia or global south or africa south of the sahara or "sub saharan africa" or subsaharan africa or africa, central or central africa or africa, northern or north africa or northern africa or magreb or maghrib or sahara or africa, southern or southern africa or africa, eastern or east africa or eastern africa or africa, western or west africa or western africa or west indies or indian ocean islands or caribbean region or

For peer review only

caribbean islands or caribbean or central america or latin america or "south and central america" or south america or asia, central or central asia or asia, northern or north asia or northern asia or asia, southeastern or southeastern asia or south eastern asia or southeast asia or south east asia or asia, western or western asia or europe, eastern or east europe or eastern europe or developing country or developing countries or developing nation? or developing population? or developing world or less developed countr* or less developed nation? or less developed population? or less developed world or lesser developed countr* or lesser developed nation? or lesser developed population? or lesser developed world or under developed countr* or under developed nation? or under developed population? or under developed world or underdeveloped countr* or underdeveloped nation? or underdeveloped population? or underdeveloped world or middle income countr* or middle income nation? or middle income population? or low income countr* or low income nation? or low income population? or lower income countr* or lower income nation? or lower income population? or underserved countr* or underserved nation? or underserved population? or underserved world or under served countr* or under served nation? or under served population? or under served world or deprived countr* or deprived nation? or deprived population? or deprived world or poor countr* or poor nation? or poor population? or poor world or poorer countr* or poorer nation? or poorer population? or poorer world or developing econom* or less developed econom* or lesser developed econom* or under developed econom* or underdeveloped econom* or middle income econom* or low income econom* or lower income econom* or low gdp or low gnp or low gross domestic or low gross national or lower gdp or lower gnp or lower gross domestic or lower gross national or lmic or lmics or third world or lami countr* or transitional countr* or emerging economies or emerging nation?).ti,ab,sh,kw. (2517925)

44 ("high burden country" or "high burden countries" or "high-burden country" or "high-burden countries" or "countdown country" or "countdown countries").ti,ab,kw,kf. (808)

45 43 or 44 [LMICs] (2518149)

46 42 and 45 [PERs, PREGNANCY - DATA COLLECTION, REGISTRIES - LMICs] (18846)

47 global health/ (16219)

48 ((global or international* or world*) and health*).ti,kw,kf. (45947)

49 exp adverse drug reaction/ (586248)

50 ((drug or drugs or medicine or medicines or medication* or pharmaceutical* or pharma-ceutical*) and expos*).ti,kw,kf. (9329)

51 (ae or co).fs. (2915988)

52 (safe or safety or side effect or side effects or undesirable effect or undesirable effects or treatment emergent or tolerability or toxicity or adrs).ti,kw,kf. (514988)

53 (adverse effect or adverse effects or adverse reaction or adverse reactions or adverse event or adverse events or adverse outcome or adverse outcomes).ti,kw,kf. (70731)

54 (AEFI and adverse).ti,kw,kf. (157)

55 "Global Alignment of Immuni#ation Safety Assessment in Pregnancy".tw,kw,kf. (20)

56 (GAIA and (alignment or immuni#ation or safety or pregnan*)).tw,kw,kf. (71)

57 exp vaccination/ (205483)

58 vaccine/ (67719)

59 exp bacterial vaccine/ or exp cell-based vaccine/ or conjugate vaccine/ or edible vaccine/ or exp fungus vaccine/ or exp inactivated vaccine/ or live vaccine/ or exp meningitis vaccine/ or exp nucleic acid vaccine/ or exp parasite vaccine/ or exp peptide vaccine/ or protein vaccine/ or exp subunit vaccine/ or exp toxoid vaccine/ or exp vector vaccine/ or virosome vaccine/ or exp virus vaccine/ (304544)

60 vaccin*.ti,kw,kf. (251220)

61 immunization/ (104541)

For peer review only

62 passive immunization/ (12623)
63 (immunit* and transfer*).ti,kw,kf. (1262)
64 (immunit* and (maternally-acqui* or passive*)).ti,kw,kf. (766)
65 ((antibod* or anti-bod*) and transfer*).ti,kw,kf. (2375)
66 ((maternal or pregnan*) and immuni#ation*).ti,kw,kf. (1766)
67 exp malaria/pc [Prevention] (14654)
68 exp antimalarial agent/ (161880)
69 (antimalarial* or anti-malarial*).ti,kw,kf. (11586)
70 exp cytomegalovirus infection/pc [Prevention] (5146)
71 respiratory syncytial virus infection/pc [Prevention] (1057)
72 exp Streptococcus infection/pc [Prevention] (10568)
73 exp Zika fever/pc [Prevention] (780)
74 exp antiretrovirus agent/ (216196)
75 (antiretroviral* or anti-retroviral* or "anti-HIV" or "anti-AIDS" or "AIDS drug" or "AIDS drugs").ti,kw,kf. (46286)
76 exp fetus development/ (29871)
77 ((fetal or foetal or fetus* or foetus*) and develop*).ti,kw,kf. (14926)
78 exp prenatal exposure/ (37294)
79 ((fetal or foetal or fetus* or foetus* or prenatal* or pre-natal*) and expos*).ti,kw,kf. (16619)
80 pregnancy outcome/ (72177)
81 ((birth or births or matern* or neonat* or neo-nat* or perinatal* or peri-natal*or peripartum or "peri-partum" or postnatal* or post-natal* or postpartum or "post-partum" or post-birth* or pregnanc*) and outcome*).ti,kw,kf. (55800)
82 ((birth or births) and defect*).ti,kw,kf. (4849)
83 child health/ (32206)
84 maternal welfare/ (15842)
85 (MNCH and (maternal* or newborn* or child*)).ti,kw,kf. (49)
86 or/47-85 [DRUGS, VACCINES, SAFETY, OUTCOMES] (4592324)
87 46 and 86 [PERs, PREGNANCY - DATA COLLECTION, REGISTRIES - LMICs - DRUGS, VACCINES, SAFETY, OUTCOMES] (5663)
88 exp animal/ or exp animal experimentation/ or exp animal model/ or exp animal experiment/ or nonhuman/ or exp vertebrate/ (30677570)
89 exp human/ or exp human experimentation/ or exp human experiment/ (23737031)
90 88 not 89 (6941713)
91 87 not 90 [ANIMAL-ONLY REMOVED] (5643)
92 editorial.pt. (729310)
93 91 not 92 [EDITORIALS REMOVED] (5614)
94 limit 93 to yr="2000-current" (5299)

***************************

**CINAHL**

| # | Query | Results |
|---|---|---|
| S92 | S90 and S91 | 878 |
| S91 | DT 2000 - 2022 | 7,386,209 |

For peer review only

| | | |
|---|---|---|
| S90 | S88 NOT S89 | 885 |
| S89 | PT editorial or opinion or commentary | 684,750 |
| S88 | S45 AND S87 | 891 |
| S87 | S46 OR S47 OR S48 OR S49 OR S50 OR S51 OR S52 OR S53 OR S54 OR S55 OR S56 OR S57 OR S58 OR S59 OR S60 OR S61 OR S62 OR S63 OR S64 OR S65 OR S66 OR S67 OR S68 OR S69 OR S70 OR S71 OR S72 OR S73 OR S74 OR S75 OR S76 OR S77 OR S78 OR S79 OR S80 OR S81 OR S82 OR S83 OR S84 OR S85 OR S86 | 354,157 |
| S86 | TI (MNCH AND (maternal* OR newborn* OR child*)) | 17 |
| S85 | (MH "Maternal-Child Health") | 3,727 |
| S84 | (MH "Child Health") | 17,073 |
| S83 | TI (birth OR births) and defect* | 969 |
| S82 | TI (birth OR births OR matern* OR neonat* OR neo-nat* OR perinatal* OR peri-natal* OR peripartum OR "peri-partum" OR postnatal* OR post-natal* OR postpartum OR "post-partum" OR post-birth* OR pregnanc*) AND outcome* | 17,343 |
| S81 | (MH "Pregnancy Outcomes") | 27,127 |
| S80 | TI (fetal OR foetal OR fetus* OR foetus* or prenatal* or pre-natal*) AND expos* | 2,603 |
| S79 | (MH "Prenatal Exposure Delayed Effects") | 6,314 |
| S78 | TI (fetal OR foetal OR fetus* OR foetus*) AND develop* | 1,028 |
| S77 | (MH "Fetal Development") | 7,851 |
| S76 | TI antiretroviral* OR anti-retroviral* OR "anti-HIV" OR "anti-AIDS" OR "AIDS drug" OR "AIDS drugs" | 9,126 |
| S75 | (MH "Anti-Retroviral Agents+") | 24,911 |
| S74 | (MH "Zika Virus Infections/PC") | 168 |
| S73 | (MH "Streptococcal Infections+/PC") | 2,280 |
| S72 | (MH "Respiratory Syncytial Virus Infections/PC") | 583 |
| S71 | (MH "Hepatitis E/PC") | 69 |
| S70 | (MH "Cytomegalovirus Infections+/PC") | 509 |
| S69 | TI antimalarial* or (anti W0 malarial*) | 714 |
| S68 | (MH "Antimalarials+") | 8,320 |
| S67 | (MH "Malaria/PC") | 3,293 |

For peer review only

| | | |
|---|---|---|
| S66 | TI (maternal* or pregnan*) and immuni?ation* | 253 |
| S65 | TI ((antibod* or (anti W0 bod*)) and transfer*) | 79 |
| S64 | TI immunit* AND (passive* or "maternally-acquired") | 13 |
| S63 | TI immunit* AND transfer* | 16 |
| S62 | (MH "Immunity, Maternally Acquired") | 232 |
| S61 | (MH "Immunization Programs") | 6,426 |
| S60 | (MH "Viral Vaccines+") | 36,206 |
| S59 | (MH "Vaccines, Combined+") | 3,562 |
| S58 | (MH "Toxoids+") | 2,372 |
| S57 | (MH "Bacterial Vaccines+") | 10,296 |
| S56 | (MH "Vaccines") | 9,533 |
| S55 | (MH "Immunization+") | 31,914 |
| S54 | TI ( GAIA AND (alignment or immuni?ation or safety or pregnan*) ) OR AB ( GAIA AND (alignment or immuni?ation or safety or pregnan*) ) | 6 |
| S53 | TI ( "Global Alignment of Immunisation Safety Assessment in Pregnancy" or "Global Alignment of Immunization Safety Assessment in Pregnancy" ) OR AB ( "Global Alignment of Immunisation Safety Assessment in Pregnancy" or "Global Alignment of Immunization Safety Assessment in Pregnancy" ) | 0 |
| S52 | TI AEFI and adverse | 5 |
| S51 | TI "adverse effect" or "adverse effects" or "adverse reaction" or "adverse reactions" or "adverse event" or "adverse events" or "adverse outcome" or "adverse outcomes" | 12,615 |
| S50 | TI safe or safety or "side effect" or "side effects" or "undesirable effect" or "undesirable effects" or "treatment emergent" or tolerability or toxicity or adrs | 113,937 |
| S49 | TI (drug or drugs or medicine or medicines or medication* or pharmaceutical* or pharma-ceutical*) AND expos* | 1,420 |
| S48 | (MH "Adverse Drug Event") | 16,207 |
| S47 | TI (global or international* or world*) and health* | 22,194 |
| S46 | (MH "World Health") | 29,391 |
| S45 | S39 AND S44 | 4,402 |
| S44 | S40 OR S41 OR S42 OR S43 | 379,212 |

For peer review only

| | | |
|---|---|---|
| S43 | TI ( "high burden country" or "high burden countries" or "high-burden country" or "high-burden countries" or "countdown country" or "countdown countries" ) OR AB ( "high burden country" or "high burden countries" or "high-burden country" or "high-burden countries" or "countdown country" or "countdown countries" ) | 188 |
| S42 | TI ( "developing country" or "developing countries" or "developing nation" or "developing nations" or "developing population" or "developing populations" or "developing world" or "less developed country" or "less developed countries" or "less developed nation" or "less developed nations" or "less developed population" or "less developed populations" or "less developed world" or "lesser developed country" or "lesser developed countries" or "lesser developed nation" or "lesser developed nations" or "lesser developed population" or "lesser developed populations" or "lesser developed world" or "under developed country" or "under developed countries" or "under developed nation" or "under developed nations" or "under developed population" or "under developed populations" or "under developed world" or "underdeveloped country" or "underdeveloped countries" or "underdeveloped nation" or "underdeveloped nations" or "underdeveloped population" or "underdeveloped populations" or "underdeveloped world" or "middle income country" or "middle income countries" or "middle income nation" or "middle income nations" or "middle income population" or "middle income populations" or "low income country" or "low income countries" or "low income nation" or "low income nations" or "low income population" or "low income populations" or "lower income country" or "lower income countries" or "lower income nation" or "lower income nations" or "lower income population" or "lower income populations" or "underserved country" or "underserved countries" or "underserved nation" or "underserved nations" or "underserved population" or "underserved populations" or "underserved world" or "under served country" or "under served countries" or "under served nation" or "under served nations" or "under served population" or "under served populations" or "under served world" or "deprived country" or "deprived countries" or "deprived nation" or "deprived nations" or "deprived population" or "deprived populations" or "deprived world" or "poor country" or "poor countries" or "poor nation" or "poor nations" or "poor population" or "poor populations" or "poor world" or "poorer country" or "poorer countries" or "poorer nation" or "poorer nations" or "poorer population" or "poorer populations" or "poorer world" or "developing economy" or "developing economies" or "less developed economy" or "less developed economics" or "lesser developed economy" or "lesser developed economies" or "under developed economy" or "under developed economies" or "underdeveloped economy" or "underdeveloped economies" or "middle income economy" or "middle income economies" or "low income economy" or "low income economies" or "lower income economy" or "lower income economies" or "low gdp" or "low gnp" or "low gross domestic" or "low gross national" or "lower gdp" or "lower gnp" or "lower gross domestic" or "lower gross national" or lmic or lmics or "third world" or "lami country" or "lami | 39,219 |

For peer review only

countries" or "transitional country" or "transitional economies" or "emerging economy" or "emerging economies" or "emerging nation" or "emerging nations" ) OR AB ( "developing country" or "developing countries" or "developing nation" or "developing nations" or "developing population" or "developing populations" or "developing world" or "less developed country" or "less developed countries" or "less developed nation" or "less developed nations" or "less developed population" or "less developed populations" or "less developed world" or "lesser developed country" or "lesser developed countries" or "lesser developed nation" or "lesser developed nations" or "lesser developed population" or "lesser developed populations" or "lesser developed world" or "under developed country" or "under developed countries" or "under developed nation" or "under developed nations" or "under developed population" or "under developed populations" or "under developed world" or "underdeveloped country" or "underdeveloped countries" or "underdeveloped nation" or "underdeveloped nations" or "underdeveloped population" or "underdeveloped populations" or "underdeveloped world" or "middle income country" or "middle income countries" or "middle income nation" or "middle income nations" or "middle income population" or "middle income populations" or "low income country" or "low income countries" or "low income nation" or "low income nations" or "low income population" or "low income populations" or "lower income country" or "lower income countries" or "lower income nation" or "lower income nations" or "lower income population" or "lower income populations" or "underserved country" or "underserved countries" or "underserved nation" or "underserved nations" or "underserved population" or "underserved populations" or "underserved world" or "under served country" or "under served countries" or "under served nation" or "under served nations" or "under served population" or "under served populations" or "under served world" or "deprived country" or "deprived countries" or "deprived nation" or "deprived nations" or "deprived population" or "deprived populations" or "deprived world" or "poor country" or "poor countries" or "poor nation" or "poor nations" or "poor population" or "poor populations" or "poor world" or "poorer country" or "poorer countries" or "poorer nation" or "poorer nations" or "poorer population" or "poorer populations" or "poorer world" or "developing economy" or "developing economies" or "less developed economy" or "less developed economics" or "lesser developed economy" or "lesser developed economies" or "under developed economy" or "under developed economies" or "underdeveloped economy" or "underdeveloped economies" or "middle income economy" or "middle income economies" or "low income economy" or "low income economies" or "lower income economy" or "lower income economies" or "low gdp" or "low gnp" or "low gross domestic" or "low gross national" or "lower gdp" or "lower gnp" or "lower gross domestic" or "lower gross national" or lmic or lmics or "third world" or "lami country" or "lami countries" or "transitional country" or "transitional economies" or "emerging economy" or "emerging economies" or "emerging nation" or "emerging nations" )

For peer review only

| | | |
|---|---|---|
| S41 | TI ( mali or malta or micronesia or "federated states of micronesia" or kiribati or "marshall islands" or nauru or "northern mariana islands" or palau or tuvalu or mauritania or mauritius or mexico or moldova or moldovian or mongolia or montenegro or morocco or ifni or mozambique or "portuguese east africa" or myanmar or burma or namibia or nepal or "netherlands antilles" or nicaragua or niger or nigeria or oman or muscat or pakistan or panama or "papua new guinea" or "new guinea" or paraguay or peru or philippines or philipines or phillipines or phillippines or poland or "polish people's republic" or portugal or "portuguese republic" or "puerto rico" or romania or russia or "russian federation" or ussr or "soviet union" or "union of soviet socialist republics" or rwanda or ruanda or samoa or "pacific islands" or polynesia or "samoan islands" or "navigator island" or "navigator islands" or "sao tome and principe" or "saudi arabia" or senegal or serbia or seychelles or "sierra leone" or slovakia or "slovak republic" or slovenia or melanesia or "solomon island" or "solomon islands" or "norfolk island" or "norfolk islands" or somalia or "south africa" or "south sudan" or "sri lanka" or ceylon or "saint kitts and nevis" or "st. kitts and nevis" or "saint lucia" or "st. lucia" or "saint Vincent and the grenadines" or "saint vincent" or "st. vincent" or grenadines or sudan or suriname or surinam or "dutch guiana" or "netherlands guiana" or syria or "syrian arab republic" or tajikistan or tadjikistan or tadzhikistan or tadzhik or tanzania or tanganyika or thailand or siam or "timor leste" or "east timor" or togo or "togolese republic" or tonga or "Trinidad and tobago" or trinidad or tobago or tunisia or turkey or turkmenistan or turkmen or uganda or ukraine or uruguay or uzbekistan or uzbek or vanuatu or "new hebrides" or venezuela or vietnam or "viet nam" or "middle east" or "west bank" or gaza or palestine or yemen or yugoslavia or zambia or zimbabwe or "northern rhodesia" or "global south" or "africa south of the sahara" or "sub-saharan africa" or "subsaharan africa" or "africa, central" or "central africa" or "africa, northern" or "north africa" or "northern africa" or magreb or maghrib or sahara or "africa, southern" or "southern africa" or "africa, eastern" or "east africa" or "eastern africa" or "africa, western" or "west africa" or "western africa" or "west indies" or caribbean or "central america" or "latin america" or "south and central america" or "south america" or "asia, central" or "central asia" or "asia, northern" or "north asia" or "northern asia" or "asia, southeastern" or "southeastern asia" or "south eastern asia" or "southeast asia" or "south east asia" or "asia, western" or "western asia" or "europe, eastern" or "east europe" or "eastern europe" ) OR AB ( mali or malta or micronesia or "federated states of micronesia" or kiribati or "marshall islands" or nauru or "northern mariana islands" or palau or tuvalu or mauritania or mauritius or mexico or moldova or moldovian or mongolia or montenegro or morocco or ifni or mozambique or "portuguese east africa" or myanmar or burma or namibia or nepal or "netherlands antilles" or nicaragua or niger or nigeria or oman or muscat or pakistan or panama or "papua new guinea" or "new guinea" or paraguay or peru or philippines or philipines or phillipines or phillippines or poland or "polish people's republic" or portugal or "portuguese republic" or "puerto rico" or romania or russia or "russian federation" or ussr or "soviet union" or "union of soviet socialist republics" or rwanda or ruanda | 148,518 |

| | | |
|---|---|---|
| | or samoa or "pacific islands" or polynesia or "samoan islands" or "navigator island" or "navigator islands" or "sao tome and principe" or "saudi arabia" or senegal or serbia or seychelles or "sierra leone" or slovakia or "slovak republic" or slovenia or melanesia or "solomon island" or "solomon islands" or "norfolk island" or "norfolk islands" or somalia or "south africa" or "south sudan" or "sri lanka" or ceylon or "saint kitts and nevis" or "st. kitts and nevis" or "saint lucia" or "st. lucia" or "saint Vincent and the grenadines" or "saint vincent" or "st. vincent" or grenadines or sudan or suriname or surinam or "dutch guiana" or "netherlands guiana" or syria or "syrian arab republic" or tajikistan or tadjikistan or tadzhikistan or tadzhik or tanzania or tanganyika or thailand or siam or "timor leste" or "east timor" or togo or "togolese republic" or tonga or "Trinidad and tobago" or trinidad or tobago or tunisia or turkey or turkmenistan or turkmen or uganda or ukraine or uruguay or uzbekistan or uzbek or vanuatu or "new hebrides" or venezuela or vietnam or "viet nam" or "middle east" or "west bank" or gaza or palestine or yemen or yugoslavia or zambia or zimbabwe or "northern rhodesia" or "global south" or "africa south of the sahara" or "sub-saharan africa" or "subsaharan africa" or "africa, central" or "central africa" or "africa, northern" or "north africa" or "northern africa" or magreb or maghrib or sahara or "africa, southern" or "southern africa" or "africa, eastern" or "east africa" or "eastern africa" or "africa, western" or "west africa" or "western africa" or "west indies" or caribbean or "central america" or "latin america" or "south and central america" or "south america" or "asia, central" or "central asia" or "asia, northern" or "north asia" or "northern asia" or "asia, southeastern" or "southeastern asia" or "south eastern asia" or "southeast asia" or "south east asia" or "asia, western" or "western asia" or "europe, eastern" or "east europe" or "eastern europe" ) | |
| S40 | TI ( afghanistan or albania or algeria or "american samoa" or angola or antigua or barbuda or argentina or armenia or armenian or aruba or azerbaijan or bahrain or bangladesh or barbados or belarus or byelarus or belorussia or byelorussian or belize or "british honduras" or benin or dahomey or bhutan or bolivia or "bosnia herzegovina" or bosnia or herzegovina or botswana or bechuanaland or brazil or brasil or bulgaria or "burkina faso" or "burkina fasso" or "upper volta" or burundi or urundi or "cabo verde" or "cape verde" or cambodia or kampuchea or "khmer republic" or cameroon or cameron or cameroun or "central african republic" or "ubangi shari" or chad or chile or china or colombia or comoros or "comoro islands" or "iles comores" or mayotte or "democratic republic of the congo" or "democratic republic congo" or congo or zaire or "costa rica" or "cote d’ivoire" or "cote d’ ivoire" or "cote divoire" or "cote d ivoire" or "ivory coast" or croatia or cuba or cyprus or "czech republic" or czechoslovakia or djibouti or "french somaliland" or dominica or "dominican republic" or ecuador or egypt or "united arab republic" or "el salvador" or "equatorial guinea" or "spanish guinea" or eritrea or estonia or eswatini or swaziland or ethiopia or fiji or gabon or "gabonese republic" or gambia or "georgia (republic)" or georgian or ghana or "gold coast" or gibraltar or greece or grenada or guam or guatemala or guinea or "guinea bissau" or guyana or "british guiana" or haiti or hispaniola or | 230,968 |

For peer review only

| | | |
|---|---|---|
| | honduras or hungary or india or indonesia or timor or iran or iraq or "isle of man" or jamaica or jordan or kazakhstan or kazakh or kenya or "democratic people’s republic of korea" or "republic of korea" or "north korea" or "south korea" or korea or kosovo or kyrgyzstan or kirghizia or kirgizstan or "kyrgyz republic" or kirghiz or laos or "lao pdr" or "lao people's democratic republic" or latvia or lebanon or lebanese republic or lesotho or basutoland or liberia or libya or "libyan arab jamahiriya" or lithuania or macau or macao or republic of "north macedonia" or macedonia or madagascar or "malagasy republic" or malawi or nyasaland or malaysia or "malay federation" or "malaya federation" or maldives or "indian ocean islands" or "indian ocean" ) OR AB ( afghanistan or albania or algeria or "american samoa" or angola or antigua or barbuda or argentina or armenia or armenian or aruba or azerbaijan or bahrain or bangladesh or barbados or belarus or byelarus or belorussia or byelorussian or belize or "british honduras" or benin or dahomey or bhutan or bolivia or "bosnia herzegovina" or bosnia or herzegovina or botswana or bechuanaland or brazil or brasil or bulgaria or "burkina faso" or "burkina fasso" or "upper volta" or burundi or urundi or "cabo verde" or "cape verde" or cambodia or kampuchea or "khmer republic" or cameroon or cameron or cameroun or "central african republic" or "ubangi shari" or chad or chile or china or colombia or comoros or "comoro islands" or "iles comores" or mayotte or "democratic republic of the congo" or "democratic republic congo" or congo or zaire or "costa rica" or "cote d’ivoire" or "cote d’ ivoire" or "cote divoire" or "cote d ivoire" or "ivory coast" or croatia or cuba or cyprus or "czech republic" or czechoslovakia or djibouti or "french somaliland" or dominica or "dominican republic" or ecuador or egypt or "united arab republic" or "el salvador" or "equatorial guinea" or "spanish guinea" or eritrea or estonia or eswatini or swaziland or ethiopia or fiji or gabon or "gabonese republic" or gambia or "georgia (republic)" or georgian or ghana or "gold coast" or gibraltar or greece or grenada or guam or guatemala or guinea or "guinea bissau" or guyana or "british guiana" or haiti or hispaniola or honduras or hungary or india or indonesia or timor or iran or iraq or "isle of man" or jamaica or jordan or kazakhstan or kazakh or kenya or "democratic people’s republic of korea" or "republic of korea" or "north korea" or "south korea" or korea or kosovo or kyrgyzstan or kirghizia or kirgizstan or "kyrgyz republic" or kirghiz or laos or "lao pdr" or "lao people's democratic republic" or latvia or lebanon or lebanese republic or lesotho or basutoland or liberia or libya or "libyan arab jamahiriya" or lithuania or macau or macao or republic of "north macedonia" or macedonia or madagascar or "malagasy republic" or malawi or nyasaland or malaysia or "malay federation" or "malaya federation" or maldives or "indian ocean islands" or "indian ocean" ) | |
| S39 | S1 OR S38 | 15,244 |
| S38 | S16 AND S37 | 15,243 |
| S37 | S17 OR S18 OR S19 OR S20 OR S21 OR S22 OR S23 OR S24 OR S25 OR S26 OR S27 OR S28 OR S29 OR S30 OR S31 OR S32 OR S33 OR S34 OR S35 OR S36 | 354,620 |

For peer review only - http://bmjopen.bmj.com/site/about/guidelines.xhtml

| | | |
|---|---|---|
| S36 | (MH "Product Surveillance+/SN") | 1 |
| S35 | (MH "Maternal Health Services+/SN") | 1,245 |
| S34 | TI pharmacovigilan* or (pharmaco W0 vigilan*) | 846 |
| S33 | (MH "Product Surveillance+") | 2,417 |
| S32 | TI "data system" or "data systems" or "information system" or "information systems" | 4,466 |
| S31 | (MH "Health Information Systems") | 3,566 |
| S30 | TI (decision* AND support* AND clinical*) | 1,865 |
| S29 | (MH "Decision Support Systems, Clinical") | 6,139 |
| S28 | TI surveillance* | 15,975 |
| S27 | (MH "Population Surveillance+") | 11,277 |
| S26 | TI survey* | 71,609 |
| S25 | (MH "Surveys") | 157,567 |
| S24 | TI registry or registries or eregistr* or "e-registry" or "e-registries" | 13,946 |
| S23 | TI (preliminary or "pilot project" or "pilot projects") AND data | 682 |
| S22 | TI "focus group" or "focus groups" | 2,957 |
| S21 | (MH "Focus Groups") | 48,414 |
| S20 | TI databas* or "data base" or "data bases" or databank* or "data bank" or "data banks" or dataset* or "data set" or "data sets" | 16,019 |
| S19 | TI data AND (accumulat* or accura* or assembl* or captur* or collect* or compil* or coordinat* or co-ordinat* or gather* or hub or hubs) | 4,425 |
| S18 | (MH "Data Curation") | 184 |
| S17 | (MH "Data Collection") | 49,942 |
| S16 | S2 OR S3 OR S4 OR S5 OR S6 OR S7 OR S8 OR S9 OR S10 OR S11 OR S12 OR S13 OR S14 OR S15 | 290,175 |
| S15 | TI maternal AND (fetal or fetus or foetal or foetus) | 4,004 |
| S14 | TI (maternal* or maternity) AND (care or health* or service or services) | 9,718 |
| S13 | (MH "Maternal Health Services") | 11,199 |
| S12 | TI (maternal* or maternity) AND (contact* or expos*) | 1,028 |
| S11 | (MH "Maternal Exposure") | 2,588 |

For peer review only

| | | |
|---|---|---|
| S10 | TI perinatal* or "peri-natal" or "peri-natally" or peripartum or "peri-partum" | 12,858 |
| S9 | (MH "Perinatal Care") | 5,019 |
| S8 | TI prenatal* or antenatal* or "ante-natal" or "ante-natally" or antepartum or "ante-partum" | 20,877 |
| S7 | (MH "Prenatal Care") | 19,422 |
| S6 | TI pregnan* | 80,123 |
| S5 | (MH "Pregnancy Trimesters+") | 13,521 |
| S4 | (MH "Expectant Mothers") | 11,382 |
| S3 | (MH "Pregnancy Complications+") | 106,825 |
| S2 | (MH "Pregnancy") OR (MH "Pregnancy, Multiple+") OR (MH "Pregnancy, Unplanned") OR (MH "Pregnancy, Unwanted") OR (MH "Pregnancy, High Risk") OR (MH "Pregnancy, Prolonged") | 227,240 |
| S1 | TI ( "pregnancy exposure database" OR "pregnancy exposure databases" OR "pregnancy exposure data base" OR "pregnancy exposure data bases" OR "pregnancy exposure registry" OR "pregnancy exposure registries" ) OR AB ( "pregnancy exposure database" OR "pregnancy exposure databases" OR "pregnancy exposure data base" OR "pregnancy exposure data bases" OR "pregnancy exposure registry" OR "pregnancy exposure registries" ) | 7 |

**Global Index Medicus**

tw:((tw:(pregnan* exposure* database*)) OR (tw:(pregnan* exposure* "data base")) OR (tw:(pregnan* exposure* "data bases")) OR (tw:(pregnan* exposure* databank*)) OR (tw:(pregnan* exposure* "data bank")) OR (tw:(pregnan* exposure* "data banks")) OR (tw:(pregnan* exposure* register)) OR (tw:(pregnan* exposure* registries)) OR (tw:(pregnan* exposure* registry)) OR (tw:(pregnan* exposure* registries))) – *108 records*

tw:((ti:((pregnan* OR prenatal* OR antenatal* OR "ante-natal" OR "ante-natally" OR antepartum OR "ante-partum" OR perinatal* OR "peri-natal" OR "peri-natally" OR peripartum OR "peri-partum" OR maternal*) AND (data OR database* OR databank* OR register OR registers OR registry OR registries OR survey* OR surveillance OR pharmacovigilan* OR "pharmaco-vigilance") AND (safe OR safety OR "side effect" of "side effects" OR "undesirable effect" OR "undesirable effects" OR "treatment emergent" OR tolerability OR toxicity OR adrs OR aefi)))) – *361 records*

tw:(ti:((pregnan* OR prenatal* OR antenatal* OR "ante-natal" OR "ante-natally" OR antepartum OR "ante-partum" OR perinatal* OR "peri-natal" OR "peri-natally" OR peripartum OR "peri-partum" OR maternal*) AND (data OR database* OR databank* OR register OR registers OR registry OR registries OR survey* OR surveillance OR pharmacovigilan* OR "pharmaco-vigilance") AND (MNCH or "maternal health" or "child health" or "infant health" or "birth defect" or "birth defects" or "birth outcome" or

For peer review only

"birth outcomes" or "pregnancy outcome" or "pregnancy outcomes" or "neonatal outcome" or "neonatal outcomes"))) – *50 records*

tw:(ti:((pregnan* OR prenatal* OR antenatal* OR "ante-natal" OR "ante-natally" OR antepartum OR "ante-partum" OR perinatal* OR "peri-natal" OR "peri-natally" OR peripartum OR "peri-partum" OR maternal*) AND (data OR database* OR databank* OR register OR registers OR registry OR registries OR survey* OR surveillance OR pharmacovigilan* OR "pharmaco-vigilance") AND (vaccine or vaccines or vaccination* or immunization* or immunization* or “maternally-acquired”))) – *0 records*

tw:((ti:((pregnan* OR prenatal* OR antenatal* OR "ante-natal" OR "ante-natally" OR antepartum OR "ante-partum" OR perinatal* OR "peri-natal" OR "peri-natally" OR peripartum OR "peri-partum" OR maternal*) AND (data OR database* OR databank* OR register OR registers OR registry OR registries OR survey* OR surveillance OR pharmacovigilan* OR "pharmaco-vigilance") AND (drug OR drugs OR medicine OR medicines OR medication* OR pharmaceutical* OR "pharma-ceutical" OR "pharma-ceuticals" OR vaccin* OR immun* OR antimalarial* OR "anti-malarial" OR "anti-malarials" OR antiviral* OR "anti-viral" OR "anti-virals" OR antiretroviral* OR "anti-retroviral" OR "anti-retrovirals" OR "Anti-HIV" OR "Anti-AIDS")))) – *10 records*

tw:((ti:((pregnan* OR prenatal* OR antenatal* OR "ante-natal" OR "ante-natally" OR antepartum OR "ante-partum" OR perinatal* OR "peri-natal" OR "peri-natally" OR peripartum OR "peri-partum" OR maternal*) AND (data OR database* OR databank* OR register OR registers OR registry OR registries OR survey* OR surveillance OR pharmacovigilan* OR "pharmaco-vigilance") AND (fetal* OR foetal* OR fetus* OR foetus* OR neonat* OR newborn* OR infant OR infants OR infanc* OR child*) ))) – *125 records*

tw:((ti:((pregnan* OR prenatal* OR antenatal* OR "ante-natal" OR "ante-natally" OR antepartum OR "ante-partum" OR perinatal* OR "peri-natal" OR "peri-natally" OR peripartum OR "peri-partum" OR maternal*)  AND (data OR database* OR databank* OR register OR registers OR registry OR registries OR survey* OR surveillance OR pharmacovigilan* OR "pharmaco-vigilance"))) AND (tw:(drug OR drugs OR medicine OR medicines OR medication* OR pharmaceutical* OR "pharma-ceutical" OR "pharma-ceuticals" OR vaccin* OR immunisation* OR immunization* OR antimalarial* OR "anti-malarial" OR "anti-malarials" OR antiviral* OR "anti-viral" OR "anti-virals" OR antiretroviral* OR "anti-retroviral" OR "anti-retrovirals" OR "Anti-HIV" OR "Anti-AIDS"))) – *71 records*

**TOTAL: 725 records**

## Google Scholar

"pregnant|pregnancy|prenatal"+"registry|registries|surveillance|pharmacovigilance" +

"expose|exposed|exposes|exposure|exposures"+[names of LMICs]

For peer review only

# Data Extraction Form

# PART 1: STUDY IDENTIFICATION / INCLUSION STATUS

Resource title:

Author(s):

Year of publication:

Resource type:

1. ☐ 1 - Peer reviewed journal
2. ☐ 2 - Grey literature
3. ☐ 3 - Registry
4. ☐ 4 - Website
5. ☐ 5 - Key informant survey / interview
6. ☐ Other

Database resource pulled from:

Final status:

1. ○ 1 - included
2. ○ 2 - excluded
3. ○ 3 - incomplete/unclear

If excluded, explain reasoning:

For peer review only

# PART 2: REGISTRY INFORMATION OR OTHER RESOURCE

Name of registry or other resource:

Primary goals/aims:

Funding source:

Years of operation:

Current status

1. Active
2. Inactive
3. On hold

Country(ies) where located:

Country representativeness:

1. Multi-national
2. National
3. State/Provincial
4. District
5. Sub-district
6. Community
7. Health clinic
8. Hospital
9. Other

Drug/vaccine exposure name and type:

Duration of follow up:

For peer review only

Current sample size:

1. <500 participants
2. 500 - 1,999 participants
3. 2,000 - 5,000 participants
4. 5,001 - 10,000 participants
5. 10,001 - 20,000 participants
6. >20,000 participants

Data collection:

1. Administrative databases
2. Medical databases
3. Registries
4. Research study
5. Program implementation
6. Other

Methodology:

Terminology and data system used:

# PART 3: CHARACTERISTICS OF INCLUDED POPULATION

Age ranges included:

Target population:

1. Pregnant women
2. Non-pregnant women / women of reproductive age
3. Children
4. Infants (<28 days old)
5. General population
6. Other

For peer review only

Gestational age

1. First trimester
2. Second trimester
3. Third trimester

Underlying medical condition(s):

Maternal outcome(s) recorded:

1. Thrombosis and/or thrombocytopenia syndrome
2. Antenatal hospitalization (not including delivery)
3. Disability
4. Gestational diabetes
5. Gestational hypertension
6. Premature rupture of membranes
7. Preterm labor
8. Preeclampsia / Eclampsia
9. Post-partum hemorrhage
10. Antenatal hemorrhage
11. Corporeal infection
12. Spontaneous abortion / miscarriage / pregnancy loss (example: prior to 20 weeks gestation)
13. Maternal death (example: within 42 days of termination of pregnancy)
14. Late maternal death (example: >42 days of termination of pregnancy)
15. Other

Maternal death cause:

1. Direct cause
2. Indirect cause

Neonatal outcome(s) recorded:

1. Preterm birth
2. Small size for gestational age / restricted fetal growth
3. Still birth (death after 28 weeks of pregnancy but before birth)
4. Live birth
5. Congenital anomaly / birth defect
6. Death
7. Other

For peer review only

Neonatal death timeframe

1. Early neonatal (0-7 days)
2. Late neonatal (8-28 days)
3. Post neonatal (29 days - 1 year)

Which congenital anomaly does this monitor (if any)?

Infant/child outcome(s) recorded:

1. Infections
2. Respiratory illness
3. Developmental outcomes
4. Other

What is the duration of follow up?

# PART 4: KEY FINDINGS

# Populate as best you can if information is provided; answer N/A if it is not relevant or N/P if it is not provided.

Strengths of the registry or other resource:

Weaknesses / gaps of registry or other resource:

Challenges of registry or other resource in its specific context:

Does this resource have the possibility to add new interventions?

1. Yes
2. No
3. Maybe
4. Unknown

For peer review only



Why?

Can this resource be combined with other systems?

1. Yes
2. No
3. Maybe
4. Unknown

Why?

Any upcoming changes to the resource:

Additional comments

For peer review only

BMJ Open

# Pregnancy Exposure Registries for Drugs and Vaccines in Low- and Middle-Income Countries: Scoping Review Protocol



| | |
|---|---|
| Journal: | *BMJ Open* |
| Manuscript ID | bmjopen-2022-070543.R2 |
| Article Type: | Protocol |
| Date Submitted by the Author: | 02-Apr-2023 |
| Complete List of Authors: | AbuShweimeh, Rahmeh; University of Washington, School of Public Health<br>Knudson, Sophie; PATH, Center for Vaccine Innovation and Access<br>Chaabane, Sonia; World Health Organization, Regulation and Safety<br>Pal, Shanthi Narayan; World Health Organization<br>Skidmore, Becky; 30. Independent Information Specialist<br>Stergachis, Andy; University of Washington School of Public Health, Pharmacy;<br>Bhat, Niranjan ; Center for Vaccine Innovation and Access, PATH, |
| <b>Primary Subject Heading</b>: | Obstetrics and gynaecology |
| Secondary Subject Heading: | Epidemiology, Pharmacology and therapeutics |
| Keywords: | CLINICAL PHARMACOLOGY, Maternal medicine < OBSTETRICS, EPIDEMIOLOGY |
| | |



SCHOLARONE™
Manuscripts

# Pregnancy Exposure Registries for Drugs and Vaccines in Low- and Middle-Income Countries: Scoping Review Protocol

**Authors:** Rahmeh AbuShweimeh[1], Sophia Knudson[2], Sonia Chaabane[3], Shanthi Narayan Pal[3], Becky Skidmore,[4] Andy Stergachis[1,5], Niranjan Bhat[2]

**Affiliations:**

1. School of Public Health, Hans Rosling Center for Population Health, University of Washington, 3980 15th Ave NE, Seattle, WA 98195
2. PATH, 2201 Westlake Ave, Suite 200, Seattle, WA 98121
3. Regulation and Safety, World Health Organization, Avenue Appia 20, 1211, Geneva, Switzerland
4. Independent Information Specialist, Ottawa, Canada
5. School of Pharmacy, University of Washington, Box 357631, Seattle, WA 98195

**Corresponding Author:**

Dr. Andy Stergachis; stergach@uw.edu

## Abstract

***Introduction***: Data regarding the safety of drugs and vaccines in pregnant women are typically unavailable before licensure. Pregnancy exposure registries (PERs) are an important source of post-marketing safety information. PERs in low- and middle-income countries (LMICs) are uncommon but can provide valuable safety data regarding their distinct contexts and will become more relevant as the introduction and use of new drugs and vaccines in pregnancy increase worldwide. Strategies to support PERs in LMICs must be based on a better understanding of



For peer review only - http://bmjopen.bmj.com/site/about/guidelines.xhtml

their current status. We developed a scoping review protocol to assess the landscape of PERs that operate in LMICs and characterize their strengths and challenges.

***Methods and Analysis*** This scoping review protocol follows the Joanna Briggs Institute (JBI) manual for scoping reviews. The search strategy will be reported using the Preferred Reporting Items for Systematic reviews and Meta-Analyses extension for Scoping Reviews (PRISMA-ScR) Checklist. We will search PubMed, Embase, CINAHL, and WHO's Global Index Medicus, as well as the reference lists of retrieved full-text records, for articles published between 2000 and 2022 that describe PERs or other resources that systematically record exposures to medical products during pregnancy and maternal and infant outcomes in LMICs. Title and abstracts will be screened by two authors and data extracted using a standardized form. We will undertake a grey literature search using Google Scholar and targeted websites. We will distribute an online survey to selected experts and conduct semi-structured interviews with key informants. Identified PERs will be summarized in tables and analyzed.

***Ethics and dissemination*** Ethical approval is not required for this activity, as it was determined not to involve human subjects research. Findings will be submitted to an open access peer-reviewed journal and may be presented at conferences, with underlying data and other materials made publicly available.

***Registration*** Open Science Framework (DOI: https://doi.org/10.17605/OSF.IO/FU5AT).



For peer review only - http://bmjopen.bmj.com/site/about/guidelines.xhtml

## STRENGTHS AND LIMITATIONS OF THIS STUDY

- This scoping review protocol identifies the landscape of pregnancy exposure registries that operate in low- and middle-income countries and characterizes their strengths and challenges for use in assessing the safety of new vaccines introductions.
- The methodology is guided by the Joanna Briggs Institute Scoping Review Manual and the reporting will be informed by the PRISMA checklist and an expert technical working group.
- To augment limitations associated with searches of bibliographic databases and grey literature, experts and key informants will queried to identify additional pregnancy exposure registries and systems that record exposure to medical products during pregnancy and maternal and perinatal outcomes in low- and middle-income countries.

For peer review only



For peer review only - http://bmjopen.bmj.com/site/about/guidelines.xhtml

## Introduction

Newly introduced vaccines and drugs hold the promise of reducing morbidity and mortality among pregnant women and infants living in low- and middle-income countries (LMICs). However, since pregnant women are actively excluded from most pre-registration clinical trials, safety information for this group is rarely available at the time of a medical product's licensure or approval. (1,2) Consequently, the safety of drugs and vaccines administered during pregnancy must be evaluated throughout their life cycle, including through active surveillance approaches during the post-licensure or post-authorization phase. A commonly used method to systematically assess post-approval safety of drugs and vaccines in pregnant women and their offspring is through the use of a pregnancy exposure registry (PER). A PER is an observational study that systematically collects health information on exposure to medical products such as drugs and vaccines during pregnancy.

PERs, particularly in high-income countries (HICs), are used throughout the post-marketing phase to monitor the safety of drugs and vaccines used during pregnancy. (3,4) Pregnancy exposure registries have been used infrequently in LMICs, where there are unique challenges with respect to the knowledge of background rates for obstetric and neonatal outcomes, access to the interventions under evaluation, and the availability of data collection resources and infrastructure. This latter point underscores the limitations in capturing information on the use of vaccines and other medicines in pregnancy, as well as information on the occurrence of obstetric/perinatal complications and other outcomes, and the capacity to link these data sources together. Examples of pregnancy exposure registries that operate in LMICs include those



For peer review only - http://bmjopen.bmj.com/site/about/guidelines.xhtml

established for drugs or vaccines of particular relevance to their populations, such as those used to treat malaria or HIV, or to prevent COVID-19. (5-7) Maternal immunization (MI) is an effective method to protect women during pregnancy and their newborns, and their use in LMICs is growing. (8) MI is anticipated to increase further in the coming years as a result of the continued adoption of COVID-19 vaccines and the introduction of promising new MI-specific vaccines, such as those for respiratory syncytial virus (RSV) and group B *Streptococcus* (GBS). Pre-licensure safety data on these vaccines will largely come from HIC settings, highlighting the importance of monitoring their post-marketing safety in LMICs. (9,10)

Most research assessing the global status of drug and vaccine safety monitoring in pregnancy has focused on HICs. (11) However, one recent study assessed existing maternal, newborn, and child health (MNCH) data collection systems in LMICs that could be used to monitor drug or vaccine safety (12). Another study assessed the feasibility of use of Global Alignment of Immunization Safety Assessment in pregnancy (GAIA) case definitions for neonatal outcomes and maternal vaccination in LMICs. (13) In contrast to these broader surveillance systems, PERs focus on active data collection specifically related to medical product exposures during pregnancy and pregnancy safety outcomes, and may be conducted by private as well as public agencies. (14) An improved understanding of PERs in LMICs can better inform how future public health efforts, such as new vaccine introductions and treatment programs, can supported maternal populations. To address this need, we aim to conduct a scoping review to identify and describe PERs and other similar resources that operate in LMICs. Here, we report the methodology for our planned scoping review.



For peer review only - http://bmjopen.bmj.com/site/about/guidelines.xhtml

## Study objective

This scoping review protocol aims to identify pregnancy exposure registries, databases and other routinely collected health data that systematically record exposures to medical products during pregnancy and maternal and infant outcomes in LMICs. The scoping review will consist of a systematic search of the scientific and grey literature, supplemented by an online survey and interviews with selected key informants, as needed.

## Method

### Protocol design

This scoping review protocol follows the Joanna Briggs Institute (JBI) manual for scoping reviews, and the search strategy will be reported using the Preferred Reporting Items for Systematic reviews and Meta-Analyses extension for Scoping Reviews (PRISMA-ScR) Checklist. (15-17) This protocol has been registered with the Open Science Framework (DOI: https://doi.org/10.17605/OSF.IO/FU5AT). (18) The scoping review start date and estimated end date are July 1, 2022, and June 30, 2023, respectively.

### Review questions

Following multiple consultations with key stakeholders, three primary review questions were selected:

1. What pregnancy exposure cohorts, databases and registries exist in LMICs?
2. What types of data, processes, and tools are included in these databases and registries?
3. What are the strengths and weaknesses of the identified databases and registries?



For peer review only

In addition, two secondary questions were identified:

1. Can the pregnancy exposure registries that have been identified in LMICs be used or adapted to monitor additional new vaccines or drugs that may be introduced for pregnant women?
2. What is the potential for data harmonization and/or combining of data across databases and registries?

Based on these objectives, the following eligibility criteria for selection were developed:

**Inclusion criteria**

1. Publications and documents published or produced from January 2000 to the present, to ensure that identified registries possess features that are more relevant to current scientific and technological conditions; online sources will be accessed.
2. Populations studied are located entirely or at least partially in low- and middle-income countries. (19)
3. Reference to prospective and retrospective electronic or combined paper-electronic data collection systems, including demographic national registers in LMICs.
4. Reference to prospective and retrospective cohort studies, with no restrictions regarding age range other than women of childbearing age (i.e., 15-49 years of age) or underlying conditions other than pregnancy.
5. Reference to systems that collect data on exposure to one or more drugs or vaccines during pregnancy.
6. Reference to systems that collect data on pregnancy outcomes, including delivery, post-partum, and neonatal outcomes (may include an extended time frame to include birth defects detected later).



For peer review only

**Exclusion criteria**

1. Editorials, opinion pieces, promotional literature
2. Guidelines or guidance documents.
3. Reference to non-allopathic (e.g., traditional, homeopathic, or naturopathic) interventions

**Search strategy and information sources**

Using an iterative process and in consultation with the review team, an information specialist will develop a strategy in PubMed incorporating controlled vocabulary/Medical Subject Headings (e.g., "Pregnancy", "Datasets as Topic", "Product Surveillance, Postmarketing") and free text (e.g., prenatal, registries, pharmacovigilance) (see Appendix 1). We will apply a LMIC filter to focus results to the geographic regions of interest. Another information specialist will peer review the strategy using the PRESS Checklist (6). Any necessary edits will be made before finalizing in PubMed and subsequently translating the search to Embase, CINAHL, and WHO's Global Index Medicus. We will also search the reference lists of potentially relevant records and articles to ensure that our search results are as comprehensive as possible.

Additionally, we will undertake a grey literature search, including a Google Scholar search and review of relevant websites, such as industry and professional organizations, associations and alliances (e.g., Developing Countries Vaccine Manufactures Network, Society for Maternal-Fetal Medicine, American College of Obstetricians and Gynecologists, International Society for Pharmacoepidemiology, International Society of Pharmacovigilance); selected Ministries of Health (including regulatory agencies and pharmacovigilance centers) in LMICs; and selected HIC organizations, including US Food and Drug Administration, European Medicines Agency,



For peer review only - http://bmjopen.bmj.com/site/about/guidelines.xhtml

US National Institutes of Health, World Health Organization, Maternal and Child Survival Programs, Measure Evaluation, UK Teratology Information Service, UK Obstetric Surveillance System, and selected academic and other non-governmental groups. The final search strategy is provided in the appendix.

## Study selection

Records retrieved by the search strategy will be downloaded to EndNote Version 9.3.3 (Clarivate) for de-duplication and then uploaded to review management software (Covidence) to enable independent screening and track disagreements and consensuses among reviewers. Each title and abstract will be screened by two independent reviewer authors to determine eligibility. Each item will be categorized into one of three categories (Yes, Maybe, No), and disagreements between reviewers will be resolved by a third reviewer. After title and abstract screening, a second round of screening will be conducted for full-text review by two reviewers and a decision will be made for data extraction. An adapted version of the PRISMA flow diagram will be constructed to summarize the number of records screened, assessed for eligibility, and included in the review, with reasons for exclusions at each stage. (7)

### Data extraction

A form for data extraction will be used to extract key information regarding the registries from the selected full-text articles and screened grey literature (see Appendix 2). The form was pilot-tested and refined during the full-text screening stage in order to capture information more efficiently. Key data elements to be collected from the included articles include the following:

a. Author(s)
b. Year of publication



For peer review only - http://bmjopen.bmj.com/site/about/guidelines.xhtml

c. Country(ies) where the registry is located.

d. Name and aims/purpose of the registry.

e. Years of registry operation

f. Characteristics of the included population (specifically, the characteristics or eligibility criteria used to be included in the registry)

g. Country representativeness (local/national/regional)

h. Funding source for the registry.

i. Current sample size (proportion in LMIC)

j. Methodology/methods, including database type.

k. Terminology and data system used (e.g., MedDRA, ICD, Brighton Collaboration)

l. Intervention/Exposure type (e.g., drugs, vaccines), comparator and details of these, including trimester of exposure.

m. Maternal, perinatal, and neonatal outcomes including, but not limited to spontaneous abortions, stillbirths, congenital anomalies, and details of these. In addition, documenting whether GAIA definitions or other standardized classifications are used.

n. Duration of follow-up.

o. Key findings that relate to the scoping review questions, including strengths, weaknesses, ability to add new interventions, and ability to combine data with other systems.

p. Demographic factors, socioeconomic factors, and lifestyle factors.



For peer review only - http://bmjopen.bmj.com/site/about/guidelines.xhtml

Data will be entered into a database that will allow searching and categorization according to selected characteristics.

**Informant Survey and Interviews**

In addition to searching bibliographic databases and grey literature, an online survey will be sent to experts and key informants to identify additional pregnancy exposure registries, surveillance registries, databases and routinely collected data that record exposure to medical products during pregnancy and maternal and perinatal outcomes in LMICs that may not have been captured or to provide additional detail on those already identified. Information obtained through key informant interviews will be subject to the same inclusion and exclusion criteria as used with the literature. A subset of survey respondents will be identified for semi-structured interviews if additional information is needed about the registries. Interviewees will be asked to discuss the strengths and weaknesses of the registry, relevant contextual factors, and the usability of these resources in their particular settings. All responses will be recorded in an electronic database for analysis.

**Data analysis**

Identified PERs will be summarized in tables organized according to relevant characteristics, including geography, methodology, types of interventions included, outcomes captured, and citations. The selected PERs will be further grouped and evaluated based on the primary and secondary scoping review questions (strengths, weaknesses, ability to add new interventions, and ability to combine data with other systems) and other methods of appraisal, and the quality of the



For peer review only - http://bmjopen.bmj.com/site/about/guidelines.xhtml

existing registries may be discussed as part of the findings. Geographic coverage or other spatial characteristics may be presented using maps.

**Consultation**

As part of this activity, a technical working group was established to provide assistance and guidance throughout the course of the review. Members of this group are experts in multiple disciplines, including pharmacovigilance, perinatology, and pediatrics, particularly as practiced in low-resource settings. In addition to this group, the protocol and results of this scoping review will be reviewed by an Expert Steering Committee on Safety Surveillance in Pregnancy in LMICs, established by WHO. Feedback from both groups will be incorporated to produce a final document.

## Patient and public involvement

There will be no patient or public involvement in this scoping review.

## Dissemination and ethics

Ethical approval is not required for this activity, though it was reviewed by PATH's Research Determination Committee and deemed not to be human-subjects research.

Results of this landscape analysis will be submitted to an open-access peer-reviewed journal for publication and may be presented at conferences, while underlying data and other materials will be made publicly available.



For peer review only - http://bmjopen.bmj.com/site/about/guidelines.xhtml

For peer review only

## Funding

This work was supported by a grant from the Bill & Melinda Gates Foundation [grant number INV-037810]. The funders had no role in the design, analysis, or writing of this manuscript.

## Acknowledgments

The authors would like to thank Kaitryn Campbell for peer review of the PubMed search strategy. We also thank the members of our Technical Working Group: Pierre Buekens, Ushma Mehta, Deshayne Fell, Smaragda Lamprianou, Kate Fay, Ajoke Sobano ter-Meulen, Nancy Salts, and Jessica Fleming. The authors alone are responsible for the views expressed in this article and they do not necessarily represent the views, decisions or policies of the institutions with which they are affiliated.

## Competing Interest

No competing interest.

## Author Contributions Statement

NB was responsible for conception and design of the review. BS developed the search strategies. SK and RAS are conducting the initial review of manuscripts. NB and AS are second reviewers. SC and SNP provided expert review and input into the protocol. RAS and AS were responsible for producing the initial draft of the review protocol. All authors provided significant editorial comments on the protocol drafts and read and approved the final manuscript.



For peer review only

## References

1. White NJ, McGready RM, Nosten FH. New medicines for tropical diseases in pregnancy: Catch-22. PLoS Med 2008;5(6): e133.

2. David AL, Ahmadzia H, Ashcroft R, Bucci-Rechtweg C, Spencer RN, Thornton S. Improving development of drug treatments for pregnant women and the fetus. Ther Innov Regul Sci. 2022 Nov;56(6):976-990.

3. Gelperin K, Hammad H, Leishear K, Bird ST, Taylor L, Hampp C, Sahin L. A systematic review of pregnancy exposure registries: examination of protocol-specified pregnancy outcomes, target sample size, and comparator selection. Pharmacoepidemiol Drug Saf. 2017 Feb;26(2):208-214.

4. Roque Pereira L, Durán CE, Layton D, Poulentzas G, Lalagkas PN, Kontogiorgis C, Sturkenboom M. A Landscape Analysis of Post-Marketing Studies Registered in the EU PAS Register and ClinicalTrials.gov Focusing on Pregnancy Outcomes or Breastfeeding Effects: A Contribution from the ConcePTION Project. Drug Saf. 2022 Apr;45(4):333-344.

5. Tinto H, Sevene E, Dellicour S, Calip GS, d'Alessandro U, Macete E, Nakanabo-Diallo S, Kazienga A, Valea I, Sorgho H, Valá A, Augusto O, Ruperez M, Menendez C, Ouma P, Desai M, ter Kuile F, Stergachis A. Assessment of the safety of antimalarial drug use during early pregnancy (ASAP): protocol for a multicenter prospective cohort study in Burkina Faso, Kenya and Mozambique. Reproductive Health. 2015 Dec, 12:112.

6. Mehta U, Clerk C, Allen E, Yore M, Sevene E, Singlovic J, Petzold M, Mangiaterra V, Elefant E, Sullivan FM, Holmes LB, Gomes M. Protocol for a drugs exposure pregnancy registry for implementation in resource-limited settings. BMC Pregnancy Childbirth. 2012 Sep 3;12:89.



For peer review only - http://bmjopen.bmj.com/site/about/guidelines.xhtml

7. Clinicaltrials.gov. COVID-19 Vaccines International Pregnancy Exposure Registry (C-VIPER). https://clinicaltrials.gov/ct2/show/NCT04705116. Accessed October 25, 2022.

8. Vargesson N. Thalidomide-induced teratogenesis: History and mechanisms. Birth Defects Res C Embryo Today. 2015;105(2):140-156.

9. Engmann C, Fleming JA, Khan S, et al. Closer and closer? Maternal immunization: current promise, future horizons. J Perinatol. 2020;40(6):844-857. doi:10.1038/s41372-020-0668-3

10. Sobanjo-ter Meulen A, Munoz FM, Kaslow DC, Klugman KP, Omer SB, Vora P, Stergachis A. Maternal Interventions Vigilance Harmonization in Low- and Middle-Income Countries: Stakeholder Meeting Report; Amsterdam, May 1-2, 2018. Vaccine. 2019 May 6;37(20):2643-2650.

11. Gelperin K, Hammad H, Leishear K, Bird ST, Taylor L, Hampp C, Sahin L. A systematic review of pregnancy exposure registries: examination of protocol-specified pregnancy outcomes, target sample size, and comparator selection. Pharmacoepidemiol Drug Saf. 2017 Feb;26(2):208-214.

12. Berrueta M, Ciapponi A, Bardach A, Cairoli FR, Castellano FJ, Xiong X, Stergachis A, Zaraa S, Sobanjo-ter Meulen A, Buekens P and Scoping Review Collaboration Group. Maternal and neonatal data collection systems in low- and middle-income countries for maternal vaccines active safety surveillance systems: A scoping review. BMC Pregnancy and Childbirth. 2021 Mar 17;21:217.

13. Stuurman AL, Riera M, Lamprianou S, Perez-Vilar S, Anderson SA, Mangtani P, Devlieger H, Verstraeten T, Zuber PLF, Guillard Maure C. Vaccine safety surveillance in pregnancy in



For peer review only

low- and middle-income countries using GAIA case definitions: A feasibility assessment. Vaccine. 2018 Oct 29;36(45):6736-6743.

14. Gliklich RE, Dreyer NA, Leavy MB, editors. Registries for Evaluating Patient Outcomes: A User's Guide [Internet]. 3rd edition. Rockville (MD): Agency for Healthcare Research and Quality (US); 2014 Apr. 21, Pregnancy Registries. Available from: https://www.ncbi.nlm.nih.gov/books/NBK208605

15. Chapter 11: Scoping reviews – JBI Manual for Evidence Synthesis – JBI Global Wiki. Accessed March 7, 2022. https://jbi-global-wiki.refined.site/space/MANUAL/3283910770/Chapter+11%3A+Scoping+reviews.

16. McGowan J, Sampson M, Salzwedel DM, Cogo E, Foerster V, Lefebvre C. PRESS Peer Review of Electronic Search Strategies: 2015 Guideline Statement. J Clin Epidemiol. 2016;75:40-46.

17. Tricco AC, Lillie E, Zarin W, et al. PRISMA Extension for Scoping Reviews (PRISMA-ScR): Checklist and Explanation. Ann Intern Med. 2018;169(7):467-473.

18. OSF Registries. Accessed July 8, 2022. https://osf.io/registries.

19. World Bank Country and Lending Groups. World Bank. https://datahelpdesk.worldbank.org/knowledgebase/articles/906519-world-bank-country-and-lending-groups. Accessed July 8, 2022.



For peer review only

For peer review only - http://bmjopen.bmj.com/site/about/guidelines.xhtml

# Search Strategy by Database:

**PubMed**

| Set # | Query | Results |
|---|---|---|
| 132 | #130 NOT #131 | 2,016 |
| 131 | address[Publication Type] OR autobiography[Publication Type] OR bibliography[Publication Type] OR biography[Publication Type] OR comment[Publication Type] OR dictionary[Publication Type] OR directory[Publication Type] OR editorial[Publication Type] OR "expression of concern"[Publication Type] OR festschrift[Publication Type] OR historical article[Publication Type] OR interactive tutorial[Publication Type] OR lecture[Publication Type] OR news[Publication Type] OR newspaper article[Publication Type] OR portrait[Publication Type] OR video-audio media[Publication Type] OR webcast[Publication Type] | 2,050,653 |
| 130 | #128 NOT #129 | 2,048 |
| 129 | Animals[mesh] NOT Humans[mesh] | 5,016,862 |
| 128 | #126 AND #127 | 2,051 |
| 127 | ("2000"[Date - Publication] : "3000"[Date - Publication]) | 20,721,844 |
| 126 | #61 AND #125 | 2,597 |
| 125 | #62 OR #63 OR #64 OR #65 OR #66 OR #67 OR #68 OR #69 OR #70 OR #71 OR #72 OR #73 OR #74 OR #75 OR #76 OR #77 OR #78 OR #79 OR #80 OR #81 OR #82 OR #83 OR #84 OR #85 OR #86 OR #87 OR #88 OR #89 OR #90 OR #91 OR #92 OR #93 OR #94 OR #95 OR #96 OR #97 OR #98 OR #99 OR #100 OR #101 OR #102 OR #103 OR #104 OR #105 OR #106 OR #107 OR #108 OR #109 OR #110 OR #111 OR #112 OR #113 OR #114 OR #115 OR #116 OR #117 OR #118 OR #119 OR #120 OR #121 OR #122 OR #123 OR #124 | 7,704,889 |
| 124 | MNCH[Other Term] AND (maternal*[Other Term] OR newborn*[Other Term] OR child*[Other Term]) | 11 |
| 123 | MNCH[Title] AND (maternal*[Title] OR newborn*[Title] OR child*[Title]) | 22 |
| 122 | "Maternal Health"[mesh] | 2,142 |
| 121 | "Child Health"[mesh] | 4,447 |
| 120 | "Infant Health"[mesh] | 1,175 |
| 119 | (birth[Other Term] OR births[Other Term]) AND defect*[Other Term] | 1,029 |
| 118 | (birth[Title] OR births[Title]) AND defect*[Title] | 2,671 |

For peer review only

| | | |
|---|---|---|
| 117 | (birth[Other Term] OR births[Other Term] OR matern*[Other Term] OR neonat*[Other Term] OR neo-nat*[Other Term] OR perinatal*[Other Term] OR peri-natal*[Other Term] OR peripartum[Other Term] OR "peri-partum"[Other Term] OR postnatal*[Other Term] OR post-natal*[Other Term] OR postpartum[Other Term] OR "post-partum"[Other Term] OR post-birth*[Other Term] OR pregnanc*[Other Term]) AND outcome*[Other Term] | 9,426 |
| 116 | (birth[Title] OR births[Title] OR matern*[Title] OR neonat*[Title] OR neo-nat*[Title] OR perinatal*[Title] OR peri-natal*[Title] OR peripartum[Title] OR "peri-partum"[Title] OR postnatal*[Title] OR post-natal*[Title] OR postpartum[Title] OR "post-partum"[Title] OR post-birth*[Title] OR pregnanc*[Title]) AND outcome*[Title] | 31,066 |
| 115 | Pregnancy Outcome[mesh] | 82,242 |
| 114 | (fetal[OT] OR foetal[OT] OR fetus*[OT] OR foetus*[OT] OR prenatal*[OT] OR pre-natal*[OT]) AND expos*[OT] | 2,033 |
| 113 | (fetal[Title] OR foetal[Title] OR fetus*[Title] OR foetus*[Title] OR prenatal*[Title] OR pre-natal*[Title]) AND expos*[Title] | 10,858 |
| 112 | "Prenatal Exposure Delayed Effects"[mesh] | 33,016 |
| 111 | (fetal[Other Term] OR foetal[Other Term] OR fetus*[Other Term] OR foetus*[Other Term]) AND develop*[Other Term] | 2,531 |
| 110 | (fetal[Title] OR foetal[Title] OR fetus*[Title] OR foetus*[Title]) AND develop*[Title] | 8,734 |
| 109 | "Fetal Development"[mesh:noexp] | 9,246 |
| 108 | antiretroviral*[Other Term] OR anti-retroviral*[Other Term] OR "anti-HIV"[Other Term] OR "anti-AIDS"[Other Term] OR "AIDS drug"[Other Term] OR "AIDS drugs"[Other Term] | 7,307 |
| 107 | (antiretroviral*[Title] OR anti-retroviral*[Title] OR "anti-HIV"[Title] OR "anti-AIDS"[Title] OR "AIDS drug"[Title] OR "AIDS drugs"[Title]) | 29,826 |
| 106 | "Anti-Retroviral Agents"[mesh] | 69,532 |
| 105 | "Zika Virus Infection/prevention and control"[MeSH] | 1,096 |
| 104 | "Streptococcal Infections/prevention and control"[MeSH:noexp] | 2,944 |
| 103 | "Respiratory Syncytial Virus Infections/prevention and control"[MeSH Terms] | 1,832 |
| 102 | "Hepatitis E/prevention and control"[MeSH Terms] | 346 |
| 101 | "Cytomegalovirus Infections/prevention and control"[MeSH Terms] | 3,130 |
| 100 | antimalarial*[Other Term] OR anti-malarial*[Other Term] | 2,577 |
| 99 | antimalarial*[Title] OR anti-malarial*[Title] | 8,562 |
| 98 | Antimalarials[mesh] | 28,226 |

For peer review only

| | | |
|---|---|---|
| 97 | "Malaria/prevention and control"[MeSH Terms] | 17,528 |
| 96 | (maternal[Other Term] OR pregnan*[Other Term]) AND (immunisation*[Other Term] OR immunization*[Other Term]) | 754 |
| 95 | (maternal[Title] OR pregnan*[Title]) AND (immunisation*[Title] OR immunization*[Title]) | 1,153 |
| 94 | (antibod*[Other Term] OR anti-bod*[Other Term]) AND transfer*[Other Term] | 338 |
| 93 | (antibod*[Title] OR anti-bod*[Title]) AND transfer*[Title] | 1,620 |
| 92 | immunit*[Other Term] AND maternally-acqui*[Other Term] | 8 |
| 91 | immunit*[Title] AND maternally-acqui*[Title] | 8 |
| 90 | immunit*[Other Term] AND transfer*[Other Term] | 161 |
| 89 | immunit*[Title] AND transfer*[Title] | 882 |
| 88 | "Immunity, Maternally-Acquired"[mesh] | 5,842 |
| 87 | "Immunization Programs"[mesh:noexp] | 12,435 |
| 86 | Immunization[mesh:noexp] | 144,668 |
| 85 | vaccin*[ti] OR vaccin*[ot] | 220,223 |
| 84 | "Viral Vaccines"[mesh] | 132,729 |
| 83 | "Vaccines, Live, Unattenuated"[mesh] | 82 |
| 82 | "Vaccines, Synthetic"[mesh] | 31,589 |
| 81 | "Vaccines, Inactivated"[mesh:noexp] | 6,082 |
| 80 | "Vaccines, Combined"[mesh] | 10,562 |
| 79 | "Vaccines, Attenuated"[mesh] | 12,810 |
| 78 | "Protozoan Vaccines"[mesh] | 6,894 |
| 77 | "Bacterial Vaccines"[mesh] | 72,365 |
| 76 | Vaccines[mesh:noexp] | 25,746 |
| 75 | Vaccination[mesh] | 100,718 |
| 74 | GAIA[Text Word] AND (alignment[Text Word] OR immunisation[Text Word] OR immunization[Text Word] OR safety[Text Word] OR pregnan*[Text Word]) | 35 |
| 73 | "Global Alignment of Immunization Safety Assessment in Pregnancy"[tw] - Schema: all | 0 |
| 72 | AEFI[Other Term] AND adverse[Other Term] | 68 |
| 71 | AEFI[Title] AND adverse[Title] | 35 |
| 70 | adverse effect[Other Term] OR adverse effects[Other Term] OR adverse reaction[Other Term] OR adverse reactions[Other Term] OR adverse event[Other Term] OR adverse events[Other Term] OR adverse outcome[Other Term] OR adverse outcomes[Other Term] | 17,729 |
| 69 | adverse effect[Title] OR adverse effects[Title] OR adverse reaction[Title] OR adverse reactions[Title] OR adverse event[Title] OR adverse events[Title] OR adverse outcome[Title] OR adverse outcomes[Title] | 31,848 |

For peer review only

| | | |
|---|---|---|
| 68 | safe[Other Term] OR safety[Other Term] OR side effect[Other Term] OR side effects[Other Term] OR undesirable effect[Other Term] OR undesirable effects[Other Term] OR treatment emergent[Other Term] OR tolerability[Other Term] OR toxicity[Other Term] OR adrs[Other Term] | 98,171 |
| 67 | safe[Title] OR safety[Title] OR side effect[Title] OR side effects[Title] OR undesirable effect[Title] OR undesirable effects[Title] OR treatment emergent[Title] OR tolerability[Title] OR toxicity[Title] OR adrs[Title] | 296,710 |
| 66 | ("adverse effects"[MeSH Subheading]) OR (Complications[MeSH Subheading]) OR ("drug effects"[MeSH Subheading]) | 6,963,724 |
| 65 | (drug[Title] OR drugs[Title] OR medicine[Title] OR medicines[Title] OR medication*[Title] OR pharmaceutical*[Title] OR pharma-ceutical*[Title]) AND expos*[Title] | 4,387 |
| 64 | "Drug-Related Side Effects and Adverse Reactions"[MeSH Terms] | 127,437 |
| 63 | (global[Title] OR international*[Title] OR world*[Title]) AND health*[Title] | 27,778 |
| 62 | Global Health[mesh] | 53,845 |
| 61 | #56 AND #60 | 8,450 |
| 60 | #57 OR #58 OR #59 | 1,104,126 |
| 59 | "high burden country"[Other Term] OR "high burden countries"[Other Term] OR "high-burden country"[Other Term] OR "high-burden countries"[Other Term] OR "countdown country"[Other Term] OR "countdown countries"[Other Term] | 10 |
| 58 | "high burden country"[Title/Abstract] OR "high burden countries"[Title/Abstract] OR "high-burden country"[Title/Abstract] OR "high-burden countries"[Title/Abstract] OR "countdown country"[Title/Abstract] OR "countdown countries"[Title/Abstract] | 633 |

For peer review only

| | | |
|---|---|---|
| 57 | afghanistan[Text Word] OR albania[Text Word] OR algeria[Text Word] OR "american samoa"[Text Word] OR angola[Text Word] OR antigua[Text Word] OR barbuda[Text Word] OR argentina[Text Word] OR armenia[Text Word] OR armenian[Text Word] OR aruba[Text Word] OR azerbaijan[Text Word] OR bahrain[Text Word] OR bangladesh[Text Word] OR barbados[Text Word] OR belarus[Text Word] OR byelarus[Text Word] OR belorussia[Text Word] OR byelorussian[Text Word] OR belize[Text Word] OR "british honduras"[Text Word] OR benin[Text Word] OR dahomey[Text Word] OR bhutan[Text Word] OR bolivia[Text Word] OR "bosnia herzegovina"[Text Word] OR bosnia[Text Word] OR herzegovina[Text Word] OR botswana[Text Word] OR bechuanaland[Text Word] OR brazil[Text Word] OR brasil[Text Word] OR bulgaria[Text Word] OR "burkina faso"[Text Word] OR "burkina fasso"[Text Word] OR "upper volta"[Text Word] OR burundi[Text Word] OR urundi[Text Word] OR "cabo verde"[Text Word] OR "cape verde"[Text Word] OR cambodia[Text Word] OR kampuchea[Text Word] OR "khmer republic"[Text Word] OR cameroon[Text Word] OR cameron[Text Word] OR cameroun[Text Word] OR "central african republic"[Text Word] OR "ubangi shari"[Text Word] OR chad[Text Word] OR chile[Text Word] OR china[Text Word] OR colombia[Text Word] OR comoros[Text Word] OR "comoro islands"[Text Word] OR "iles comores"[Text Word] OR mayotte[Text Word] OR "democratic republic of the congo"[Text Word] OR "democratic republic congo"[Text Word] OR congo[Text Word] OR zaire[Text Word] OR "costa rica"[Text Word] OR "cote d'ivoire"[Text Word] OR "cote d' ivoire"[Text Word] OR "cote divoire"[Text Word] OR "cote d ivoire"[Text Word] OR "ivory coast"[Text Word] OR croatia[Text Word] OR cuba[Text Word] OR cyprus[Text Word] OR "czech republic"[Text Word] OR czechoslovakia[Text Word] OR djibouti[Text Word] OR "french somaliland"[Text Word] OR dominica[Text Word] OR "dominican republic"[Text Word] OR ecuador[Text Word] OR egypt[Text Word] OR "united arab republic"[Text Word] OR "el salvador"[Text Word] OR "equatorial guinea"[Text Word] OR "spanish guinea"[Text Word] OR eritrea[Text Word] OR estonia[Text Word] OR eswatini[Text Word] OR swaziland[Text Word] OR ethiopia[Text Word] OR fiji[Text Word] OR gabon[Text Word] OR "gabonese republic"[Text Word] OR gambia[Text Word] OR "georgia (republic)"[Text Word] OR georgian[Text Word] OR ghana[Text Word] OR "gold coast"[Text Word] OR gibraltar[Text Word] OR greece[Text Word] OR grenada[Text Word] OR guam[Text Word] OR guatemala[Text Word] OR guinea[Text Word] OR "guinea bissau"[Text Word] OR guyana[Text Word] OR "british guiana"[Text Word] OR haiti[Text Word] OR hispaniola[Text Word] OR honduras[Text Word] OR hungary[Text Word] OR india[Text Word] OR indonesia[Text Word] OR timor[Text Word] OR iran[Text Word] OR iraq[Text Word] OR "isle | 1,103,822 |

For peer review only

of man"[Text Word] OR jamaica[Text Word] OR jordan[Text Word] OR kazakhstan[Text Word] OR kazakh[Text Word] OR kenya[Text Word] OR "democratic people’s republic of korea"[Text Word] OR "republic of korea"[Text Word] OR "north korea"[Text Word] OR "south korea"[Text Word] OR korea[Text Word] OR kosovo[Text Word] OR kyrgyzstan[Text Word] OR kirghizia[Text Word] OR kirgizstan[Text Word] OR "kyrgyz republic"[Text Word] OR kirghiz[Text Word] OR laos[Text Word] OR "lao pdr"[Text Word] OR "lao people's democratic republic"[Text Word] OR latvia[Text Word] OR lebanon[Text Word] OR lebanese republic[Text Word] OR lesotho[Text Word] OR basutoland[Text Word] OR liberia[Text Word] OR libya[Text Word] OR "libyan arab jamahiriya"[Text Word] OR lithuania[Text Word] OR macau[Text Word] OR macao[Text Word] OR republic of "north macedonia"[Text Word] OR macedonia[Text Word] OR madagascar[Text Word] OR "malagasy republic"[Text Word] OR malawi[Text Word] OR nyasaland[Text Word] OR malaysia[Text Word] OR "malay federation"[Text Word] OR "malaya federation"[Text Word] OR maldives[Text Word] OR "indian ocean islands"[Text Word] OR "indian ocean"[Text Word] OR mali[Text Word] OR malta[Text Word] OR micronesia[Text Word] OR "federated states of micronesia"[Text Word] OR kiribati[Text Word] OR "marshall islands"[Text Word] OR nauru[Text Word] OR "northern mariana islands"[Text Word] OR palau[Text Word] OR tuvalu[Text Word] OR mauritania[Text Word] OR mauritius[Text Word] OR mexico[Text Word] OR moldova[Text Word] OR moldovian[Text Word] OR mongolia[Text Word] OR montenegro[Text Word] OR morocco[Text Word] OR ifni[Text Word] OR mozambique[Text Word] OR "portuguese east africa"[Text Word] OR myanmar[Text Word] OR burma[Text Word] OR namibia[Text Word] OR nepal[Text Word] OR "netherlands antilles"[Text Word] OR nicaragua[Text Word] OR niger[Text Word] OR nigeria[Text Word] OR oman[Text Word] OR muscat[Text Word] OR pakistan[Text Word] OR panama[Text Word] OR "papua new guinea"[Text Word] OR "new guinea"[Text Word] OR paraguay[Text Word] OR peru[Text Word] OR philippines[Text Word] OR philipines[Text Word] OR phillipines[Text Word] OR phillippines[Text Word] OR poland[Text Word] OR "polish people's republic"[Text Word] OR portugal[Text Word] OR "portuguese republic"[Text Word] OR "puerto rico"[Text Word] OR romania[Text Word] OR russia[Text Word] OR "russian federation"[Text Word] OR ussr[Text Word] OR "soviet union"[Text Word] OR "union of soviet socialist republics"[Text Word] OR rwanda[Text Word] OR ruanda[Text Word] OR samoa[Text Word] OR "pacific islands"[Text Word] OR polynesia[Text Word] OR "samoan islands"[Text Word] OR "navigator island"[Text Word] OR "navigator islands"[Text Word] OR "sao tome and principe"[Text Word] OR "saudi arabia"[Text Word] OR senegal[Text Word] OR serbia[Text Word] OR

For peer review only

seychelles[Text Word] OR "sierra leone"[Text Word] OR slovakia[Text Word] OR "slovak republic"[Text Word] OR slovenia[Text Word] OR melanesia[Text Word] OR "solomon island"[Text Word] OR "solomon islands"[Text Word] OR "norfolk island"[Text Word] OR "norfolk islands"[Text Word] OR somalia[Text Word] OR "south africa"[Text Word] OR "south sudan"[Text Word] OR "sri lanka"[Text Word] OR ceylon[Text Word] OR "saint kitts and nevis"[Text Word] OR "st. kitts and nevis"[Text Word] OR "saint lucia"[Text Word] OR "st. lucia"[Text Word] OR "saint Vincent and the grenadines"[Text Word] OR "saint vincent"[Text Word] OR "st. vincent"[Text Word] OR grenadines[Text Word] OR sudan[Text Word] OR suriname[Text Word] OR surinam[Text Word] OR "dutch guiana"[Text Word] OR "netherlands guiana"[Text Word] OR syria[Text Word] OR "syrian arab republic"[Text Word] OR tajikistan[Text Word] OR tadjikistan[Text Word] OR tadzhikistan[Text Word] OR tadzhik[Text Word] OR tanzania[Text Word] OR tanganyika[Text Word] OR thailand[Text Word] OR siam[Text Word] OR "timor leste"[Text Word] OR "east timor"[Text Word] OR togo[Text Word] OR "togolese republic"[Text Word] OR tonga[Text Word] OR "Trinidad and tobago"[Text Word] OR trinidad[Text Word] OR tobago[Text Word] OR tunisia[Text Word] OR turkey[Text Word] OR turkmenistan[Text Word] OR turkmen[Text Word] OR uganda[Text Word] OR ukraine[Text Word] OR uruguay[Text Word] OR uzbekistan[Text Word] OR uzbek[Text Word] OR vanuatu[Text Word] OR "new hebrides"[Text Word] OR venezuela[Text Word] OR vietnam[Text Word] OR "viet nam"[Text Word] OR "middle east"[Text Word] OR "west bank"[Text Word] OR gaza[Text Word] OR palestine[Text Word] OR yemen[Text Word] OR yugoslavia[Text Word] OR zambia[Text Word] OR zimbabwe[Text Word] OR "northern rhodesia"[Text Word] OR "global south"[Text Word] OR "africa south of the sahara"[Text Word] OR "sub-saharan africa"[Text Word] OR "subsaharan africa"[Text Word] OR "africa, central"[Text Word] OR "central africa"[Text Word] OR "africa, northern"[Text Word] OR "north africa"[Text Word] OR "northern africa"[Text Word] OR magreb[Text Word] OR maghrib[Text Word] OR sahara[Text Word] OR "africa, southern"[Text Word] OR "southern africa"[Text Word] OR "africa, eastern"[Text Word] OR "east africa"[Text Word] OR "eastern africa"[Text Word] OR "africa, western"[Text Word] OR "west africa"[Text Word] OR "western africa"[Text Word] OR "west indies"[Text Word] OR caribbean[Text Word] OR "central america"[Text Word] OR "latin america"[Text Word] OR "south and central america"[Text Word] OR "south america"[Text Word] OR "asia, central"[Text Word] OR "central asia"[Text Word] OR "asia, northern"[Text Word] OR "north asia"[Text Word] OR "northern asia"[Text Word] OR "asia, southeastern"[Text Word] OR "southeastern asia"[Text Word] OR

For peer review only

"south eastern asia"[Text Word] OR "southeast asia"[Text Word] OR "south east asia"[Text Word] OR "asia, western"[Text Word] OR "western asia"[Text Word] OR "europe, eastern"[Text Word] OR "east europe"[Text Word] OR "eastern europe"[Text Word] OR "developing country"[Text Word] OR "developing countries"[Text Word] OR "developing nation"[Text Word] OR "developing nations"[Text Word] OR "developing population"[Text Word] OR "developing populations"[Text Word] OR "developing world"[Text Word] OR "less developed country"[Text Word] OR "less developed countries"[Text Word] OR "less developed nation"[Text Word] OR "less developed nations"[Text Word] OR "less developed population"[Text Word] OR "less developed populations"[Text Word] OR "less developed world"[Text Word] OR "lesser developed country"[Text Word] OR "lesser developed countries"[Text Word] OR "lesser developed nation"[Text Word] OR "lesser developed nations"[Text Word] OR "lesser developed population"[Text Word] OR "lesser developed populations"[Text Word] OR "lesser developed world"[Text Word] OR "under developed country"[Text Word] OR "under developed countries"[Text Word] OR "under developed nation"[Text Word] OR "under developed nations"[Text Word] OR "under developed population"[Text Word] OR "under developed populations"[Text Word] OR "under developed world"[Text Word] OR "underdeveloped country"[Text Word] OR "underdeveloped countries"[Text Word] OR "underdeveloped nation"[Text Word] OR "underdeveloped nations"[Text Word] OR "underdeveloped population"[Text Word] OR "underdeveloped populations"[Text Word] OR "underdeveloped world"[Text Word] OR "middle income country"[Text Word] OR "middle income countries"[Text Word] OR "middle income nation"[Text Word] OR "middle income nations"[Text Word] OR "middle income population"[Text Word] OR "middle income populations"[Text Word] OR "low income country"[Text Word] OR "low income countries"[Text Word] OR "low income nation"[Text Word] OR "low income nations"[Text Word] OR "low income population"[Text Word] OR "low income populations"[Text Word] OR "lower income country"[Text Word] OR "lower income countries"[Text Word] OR "lower income nation"[Text Word] OR "lower income nations"[Text Word] OR "lower income population"[Text Word] OR "lower income populations"[Text Word] OR "underserved country"[Text Word] OR "underserved countries"[Text Word] OR "underserved nation"[Text Word] OR "underserved nations"[Text Word] OR "underserved population"[Text Word] OR "underserved populations"[Text Word] OR "underserved world"[Text Word] OR "under served country"[Text Word] OR "under served countries"[Text Word] OR "under served nation"[Text Word] OR "under served nations"[Text Word] OR "under served population"[Text Word] OR "under served populations"[Text

For peer review only

| | | |
|---|---|---|
| | Word] OR "under served world"[Text Word] OR "deprived country"[Text Word] OR "deprived countries"[Text Word] OR "deprived nation"[Text Word] OR "deprived nations"[Text Word] OR "deprived population"[Text Word] OR "deprived populations"[Text Word] OR "deprived world"[Text Word] OR "poor country"[Text Word] OR "poor countries"[Text Word] OR "poor nation"[Text Word] OR "poor nations"[Text Word] OR "poor population"[Text Word] OR "poor populations"[Text Word] OR "poor world"[Text Word] OR "poorer country"[Text Word] OR "poorer countries"[Text Word] OR "poorer nation"[Text Word] OR "poorer nations"[Text Word] OR "poorer population"[Text Word] OR "poorer populations"[Text Word] OR "poorer world"[Text Word] OR "developing economy"[Text Word] OR "developing economies"[Text Word] OR "less developed economy"[Text Word] OR "less developed economics"[Text Word] OR "lesser developed economy"[Text Word] OR "lesser developed economies"[Text Word] OR "under developed economy"[Text Word] OR "under developed economies"[Text Word] OR "underdeveloped economy"[Text Word] OR "underdeveloped economies"[Text Word] OR "middle income economy"[Text Word] OR "middle income economies"[Text Word] OR "low income economy"[Text Word] OR "low income economies"[Text Word] OR "lower income economy"[Text Word] OR "lower income economies"[Text Word] OR "low gdp"[Text Word] OR "low gnp"[Text Word] OR "low gross domestic"[Text Word] OR "low gross national"[Text Word] OR "lower gdp"[Text Word] OR "lower gnp"[Text Word] OR "lower gross domestic"[Text Word] OR "lower gross national"[Text Word] OR lmic[Text Word] OR lmics[Text Word] OR "third world"[Text Word] OR "lami country"[Text Word] OR "lami countries"[Text Word] OR "transitional country"[Text Word] OR "transitional economies"[Text Word] OR "emerging economy"[Text Word] OR "emerging economies"[Text Word] OR "emerging nation"[Text Word] OR "emerging nations"[Text Word] | |
| 56 | #1 OR #55 | 39,866 |
| 55 | #21 AND #54 | 39,859 |
| 54 | #22 OR #23 OR #24 OR #25 OR #26 OR #27 OR #28 OR #29 OR #30 OR #31 OR #32 OR #33 OR #34 OR #35 OR #36 OR #37 OR #38 OR #39 OR #40 OR #41 OR #42 OR #43 OR #44 OR #45 OR #46 OR #47 OR #48 OR #49 OR #50 OR #51 OR #52 OR #53 | 850,027 |
| 53 | "Product Surveillance, Postmarketing/statistics and numerical data"[MeSH Terms] | 2,361 |
| 52 | "Maternal Health Services/statistics and numerical data"[MeSH Terms] | 7,290 |
| 51 | "Maternal Exposure/statistics and numerical data"[MeSH Terms] | 1,123 |

For peer review only

| | | |
|---|---|---|
| 50 | pharmacovigilan*[ti] OR pharmaco-vigilan*[ti] OR pharmacovigilan*[ot] OR pharmaco-vigilan*[ot] | 3,751 |
| 49 | "Product Surveillance, Postmarketing"[mesh:noexp] | 7,553 |
| 48 | "data system"[ti] OR "data systems"[ti] OR "information system"[ti] OR "information systems"[ti] OR "data system"[ot] OR "data systems"[ot] OR "information system"[ot] OR "information systems"[ot] | 13,581 |
| 47 | "Health Information Systems"[mesh] | 1,546 |
| 46 | (decision*[ti] AND support*[ti] AND clinical*[ti]) OR (decision*[ot] AND support*[ot] AND clinical*[ot]) | 4,467 |
| 45 | "Decision Support Systems, Clinical"[mesh] | 9,106 |
| 44 | "Databases, Factual"[mesh] | 163,862 |
| 43 | "Databases as Topic"[mesh:noexp] | 9,688 |
| 42 | surveillance*[ti] OR surveillance*[ot] | 61,304 |
| 41 | "Population Surveillance"[mesh] | 74,026 |
| 40 | survey*[ti] OR survey*[ot] | 189,840 |
| 39 | "Health Surveys"[mesh:noexp] | 66,266 |
| 38 | "Health Care Surveys"[MeSH Major Topic] | 10,167 |
| 37 | "Surveys and Questionnaires"[MeSH Major Topic:noexp] | 49,876 |
| 36 | registry[ot] OR registries[ot] OR eregistr*[ot] OR "e-registry"[ot] OR "e-registries"[ot] | 8,509 |
| 35 | registry[ti] OR registries[ti] OR eregistr*[ti] OR "e-registry"[ti] OR "e-registries"[ti] | 34,311 |
| 34 | Registries[mesh:noexp] | 104,749 |
| 33 | (preliminary[ot] OR "pilot project"[ot] OR "pilot projects"[ot]) AND data[ot] | 35 |
| 32 | (preliminary[ti] OR "pilot project"[ti] OR "pilot projects"[ti]) AND data[ti] | 2,947 |
| 31 | "Preliminary Data"[mesh] | 724 |
| 30 | "focus group"[ti] OR "focus groups"[ti] OR "focus group"[ot] OR "focus groups"[ot] | 6,048 |
| 29 | "Focus Groups"[mesh] | 34,384 |
| 28 | databas*[ot] OR "data base"[ot] OR "data bases"[ot] OR databank*[ot] OR "data bank"[ot] OR "data banks"[ot] OR dataset*[ot] OR "data set"[ot] OR "data sets"[ot] | 14,502 |
| 27 | databas*[ti] OR "data base"[ti] OR "data bases"[ti] OR databank*[ti] OR "data bank"[ti] OR "data banks"[ti] OR dataset*[ti] OR "data set"[ti] OR "data sets"[ti] | 55,119 |
| 26 | "Datasets as Topic"[mesh] | 7,252 |
| 25 | data[ot] AND (accumulat*[ot] OR accura*[ot] OR assembl*[ot] OR captur*[ot] OR collect*[ot] OR compil*[ot] OR coordinat*[ot] OR co-ordinat*[ot] OR gather*[ot] OR hub[ot] OR hubs[ot]) | 5,205 |

For peer review only

| | | |
|---|---|---|
| 24 | data[ti] AND (accumulat*[ti] OR accura*[ti] OR assembl*[ti] OR captur*[ti] OR collect*[ti] OR compil*[ti] OR coordinat*[ti] OR co-ordinat*[ti] OR gather*[ti] OR hub[ti] OR hubs[ti]) | 11,365 |
| 23 | "Data Accuracy"[mesh] | 3,726 |
| 22 | "Data Collection"[mesh:noexp] | 91,761 |
| 21 | #2 OR #3 OR #4 OR #5 OR #6 OR #7 OR #8 OR #9 OR #10 OR #11 OR #12 OR #13 OR #14 OR #15 OR #16 OR #17 OR #18 OR #19 OR #20 | 1,067,642 |
| 20 | maternal[ot] AND (fetal[ot] OR fetus[ot] OR foetal[ot] OR foetus[ot]) | 3,848 |
| 19 | (maternal*[ot] OR maternity[ot]) AND (care[ot] OR health*[ot] OR service[ot] OR services[ot]) | 11,210 |
| 18 | (maternal*[ot] or maternity[ot]) AND (contact*[ot] or expos*[ot]) | 601 |
| 17 | perinatal*[ot] OR "peri-natal"[ot] OR "peri-natally"[ot] OR peripartum[ot] or "peri-partum"[ot] | 7,552 |
| 16 | prenatal*[ot] OR antenatal*[ot] OR "ante-natal"[ot] OR "ante-natally"[ot] OR antepartum[ot] or "ante-partum"[ot] | 15,862 |
| 15 | maternal[ti] AND (fetal[ti] OR fetus[ti] OR foetal[ti] OR foetus[ti]) | 13,964 |
| 14 | (maternal*[ti] OR maternity[ti]) AND (care[ti] OR health*[ti] OR service[ti] OR services[ti]) | 13,467 |
| 13 | "Maternal Health Services"[mesh:noexp] | 15,617 |
| 12 | (maternal*[ti] or maternity[ti]) AND (contact*[ti] or expos*[ti]) | 3,847 |
| 11 | "Maternal Exposure"[mesh] | 10,689 |
| 10 | perinatal*[ti] OR "peri-natal"[ti] OR "peri-natally"[ti] OR peripartum[ti] or "peri-partum"[ti] | 30,982 |
| 9 | "Perinatal Care"[mesh] | 11,334 |
| 8 | prenatal*[ti] OR antenatal*[ti] OR "ante-natal"[ti] OR "ante-natally"[ti] OR antepartum[ti] or "ante-partum"[ti] | 59,414 |
| 7 | "Prenatal Care"[mesh] | 31,249 |
| 6 | pregnan*[ti] | 252,201 |
| 5 | "Pregnancy Trimesters"[mesh] | 43,874 |
| 4 | "Pregnant Women"[mesh] | 12,334 |
| 3 | "Pregnancy Complications"[mesh] | 460,551 |
| 2 | Pregnancy[mesh] | 970,546 |
| 1 | "pregnancy exposure database" [tw] OR "pregnancy exposure databases" [tw] OR "pregnancy exposure data base" [tw] OR "pregnancy exposure data bases" [tw] OR "pregnancy exposure registry" [tw] OR "pregnancy exposure registries" [tw] | 34 |

For peer review only

**Embase**

--------------------------------------------------------------------------------
1 ("pregnancy exposure database" or "pregnancy exposure databases" or "pregnancy exposure data base" or "pregnancy exposure data bases" or "pregnancy exposure registry" or "pregnancy exposure registries").tw,kw,kf. (113)
2 exp pregnancy/ (727815)
3 exp pregnancy complication/ (141579)
4 exp named groups by pregnancy/ (130431)
5 pregnan*.ti,kw,kf. (329712)
6 exp prenatal care/ (167165)
7 (prenatal* or antenatal* or "ante-natal" or "ante-natally" or antepartum or "ante-partum").ti,kw,kf. (87488)
8 exp perinatal care/ (65235)
9 (perinatal* or peri-natal* or peripartum or "peri-partum").ti,kw,kf. (46195)
10 maternal exposure/ (3606)
11 ((maternal* or maternity) and (contact* or expos*)).ti,kw,kf. (6357)
12 maternal health service/ (2454)
13 ((maternal* or maternity) and (care or health* or service or services)).ti,kw,kf. (22738)
14 (maternal and (fetal or fetus or foetal or foetus)).ti,kw,kf. (20741)
15 or/2-14 [PREGNANCY] (1035932)
16 *information processing/ (37078)
17 data aggregation/ (374)
18 data accuracy/ (1596)
19 (data and (accumulat* or accura* or assembl* or captur* or collect* or compil* or coordinat* or co-ordinat* or gather* or hub or hubs)).ti,kw,kf. (18259)
20 (databas* or "data base" or "data bases" or databank* or "data bank" or "data banks" or dataset* or "data set" or "data sets").ti,kw,kf. (86633)
21 (focus group or focus groups).ti,kw,kf. (7608)
22 preliminary data/ (31472)
23 ((preliminary or pilot project or pilot projects) and data).ti,kw,kf. (5481)
24 exp register/ (180900)
25 (registry or registries or eregistr* or "e-registry" or "e-registries").ti,kw,kf. (68347)
26 questionnaire/ (814172)
27 *health care survey/ (2606)
28 health survey/ (213987)
29 survey*.ti,kw,kf. (220889)
30 population surveillance/ (117)
31 surveillance*.ti,kw,kf. (82221)
32 data base/ (248533)
33 factual database/ (27912)
34 clinical decision support system/ (4716)
35 (decision* and support* and clinical*).ti,kw,kf. (5904)
36 medical information system/ (22591)
37 ("data system" or "data systems" or "information system" or "information systems").ti,kw,kf. (18222)
38 exp postmarketing surveillance/ (37924)
39 (pharmacovigilan* or pharmaco-vigilan*).ti,kw,kf. (7417)

For peer review only

40 or/16-39 [DATA COLLECTION, REGISTRIES] (1815231)
41 15 and 40 [PREGNANCY - DATA COLLECTION, REGISTRIES] (71373)
42 1 or 41 [PERs, PREGNANCY - DATA COLLECTION, REGISTRIES] (71381)
43 (afghanistan or albania or algeria or american samoa or angola or "antigua and barbuda" or antigua or barbuda or argentina or armenia or armenian or aruba or azerbaijan or bahrain or bangladesh or barbados or republic of belarus or belarus or byelarus or belorussia or byelorussian or belize or british honduras or benin or dahomey or bhutan or bolivia or "bosnia and herzegovina" or bosnia or herzegovina or botswana or bechuanaland or brazil or brasil or bulgaria or burkina faso or burkina fasso or upper volta or burundi or urundi or cabo verde or cape verde or cambodia or kampuchea or khmer republic or cameroon or cameron or cameroun or central african republic or ubangi shari or chad or chile or china or colombia or comoros or comoro islands or iles comores or mayotte or democratic republic of the congo or democratic republic congo or congo or zaire or costa rica or "cote d’ivoire" or "cote d’ ivoire" or cote divoire or cote d ivoire or ivory coast or croatia or cuba or cyprus or czech republic or czechoslovakia or djibouti or french somaliland or dominica or dominican republic or ecuador or egypt or united arab republic or el salvador or equatorial guinea or spanish guinea or eritrea or estonia or eswatini or swaziland or ethiopia or fiji or gabon or gabonese republic or gambia or "georgia (republic)" or georgian or ghana or gold coast or gibraltar or greece or grenada or guam or guatemala or guinea or guinea bissau or guyana or british guiana or haiti or hispaniola or honduras or hungary or india or indonesia or timor or iran or iraq or isle of man or jamaica or jordan or kazakhstan or kazakh or kenya or "democratic people’s republic of korea" or republic of korea or north korea or south korea or korea or kosovo or kyrgyzstan or kirghizia or kirgizstan or kyrgyz republic or kirghiz or laos or lao pdr or "lao people's democratic republic" or latvia or lebanon or lebanese republic or lesotho or basutoland or liberia or libya or libyan arab jamahiriya or lithuania or macau or macao or republic of north macedonia or macedonia or madagascar or malagasy republic or malawi or nyasaland or malaysia or malay federation or malaya federation or maldives or indian ocean islands or indian ocean or mali or malta or micronesia or federated states of micronesia or kiribati or marshall islands or nauru or northern mariana islands or palau or tuvalu or mauritania or mauritius or mexico or moldova or moldovian or mongolia or montenegro or "montenegro (republic)" or morocco or ifni or mozambique or portuguese east africa or myanmar or burma or namibia or nepal or netherlands antilles or nicaragua or niger or nigeria or oman or muscat or pakistan or panama or papua new guinea or new guinea or paraguay or peru or philippines or philipines or phillipines or phillippines or poland or "polish people's republic" or portugal or portuguese republic or puerto rico or romania or russia or russian federation or ussr or soviet union or union of soviet socialist republics or rwanda or ruanda or samoa or pacific islands or polynesia or samoan islands or navigator island or navigator islands or "sao tome and principe" or saudi arabia or senegal or serbia or seychelles or sierra leone or slovakia or slovak republic or slovenia or melanesia or solomon island or solomon islands or norfolk island or norfolk islands or somalia or south africa or south sudan or sri lanka or ceylon or "saint kitts and nevis" or "st. kitts and nevis" or saint lucia or "st. lucia" or "saint vincent and the grenadines" or saint vincent or "st. vincent" or grenadines or sudan or suriname or surinam or dutch guiana or netherlands guiana or syria or syrian arab republic or tajikistan or tadjikistan or tadzhikistan or tadzhik or tanzania or tanganyika or thailand or siam or timor leste or east timor or togo or togolese republic or tonga or "trinidad and tobago" or trinidad or tobago or tunisia or "turkey (republic)" or turkey or turkmenistan or turkmen or uganda or ukraine or uruguay or uzbekistan or uzbek or vanuatu or new hebrides or venezuela or vietnam or viet nam or middle east or west bank or gaza or palestine or yemen or yugoslavia or zambia or zimbabwe or northern rhodesia or global south or africa south of the sahara or "sub saharan africa" or subsaharan africa or africa, central or central africa or africa, northern or north africa or northern africa or magreb or maghrib or sahara or africa, southern or southern africa or africa, eastern or east africa or eastern africa or africa, western or west africa or western africa or west indies or indian ocean islands or caribbean region or

For peer review only

caribbean islands or caribbean or central america or latin america or "south and central america" or south america or asia, central or central asia or asia, northern or north asia or northern asia or asia, southeastern or southeastern asia or south eastern asia or southeast asia or south east asia or asia, western or western asia or europe, eastern or east europe or eastern europe or developing country or developing countries or developing nation? or developing population? or developing world or less developed countr* or less developed nation? or less developed population? or less developed world or lesser developed countr* or lesser developed nation? or lesser developed population? or lesser developed world or under developed countr* or under developed nation? or under developed population? or under developed world or underdeveloped countr* or underdeveloped nation? or underdeveloped population? or underdeveloped world or middle income countr* or middle income nation? or middle income population? or low income countr* or low income nation? or low income population? or lower income countr* or lower income nation? or lower income population? or underserved countr* or underserved nation? or underserved population? or underserved world or under served countr* or under served nation? or under served population? or under served world or deprived countr* or deprived nation? or deprived population? or deprived world or poor countr* or poor nation? or poor population? or poor world or poorer countr* or poorer nation? or poorer population? or poorer world or developing econom* or less developed econom* or lesser developed econom* or under developed econom* or underdeveloped econom* or middle income econom* or low income econom* or lower income econom* or low gdp or low gnp or low gross domestic or low gross national or lower gdp or lower gnp or lower gross domestic or lower gross national or lmic or lmics or third world or lami countr* or transitional countr* or emerging economies or emerging nation?).ti,ab,sh,kw. (2517925)

44 ("high burden country" or "high burden countries" or "high-burden country" or "high-burden countries" or "countdown country" or "countdown countries").ti,ab,kw,kf. (808)

45 43 or 44 [LMICs] (2518149)

46 42 and 45 [PERs, PREGNANCY - DATA COLLECTION, REGISTRIES - LMICs] (18846)

47 global health/ (16219)

48 ((global or international* or world*) and health*).ti,kw,kf. (45947)

49 exp adverse drug reaction/ (586248)

50 ((drug or drugs or medicine or medicines or medication* or pharmaceutical* or pharma-ceutical*) and expos*).ti,kw,kf. (9329)

51 (ae or co).fs. (2915988)

52 (safe or safety or side effect or side effects or undesirable effect or undesirable effects or treatment emergent or tolerability or toxicity or adrs).ti,kw,kf. (514988)

53 (adverse effect or adverse effects or adverse reaction or adverse reactions or adverse event or adverse events or adverse outcome or adverse outcomes).ti,kw,kf. (70731)

54 (AEFI and adverse).ti,kw,kf. (157)

55 "Global Alignment of Immuni#ation Safety Assessment in Pregnancy".tw,kw,kf. (20)

56 (GAIA and (alignment or immuni#ation or safety or pregnan*)).tw,kw,kf. (71)

57 exp vaccination/ (205483)

58 vaccine/ (67719)

59 exp bacterial vaccine/ or exp cell-based vaccine/ or conjugate vaccine/ or edible vaccine/ or exp fungus vaccine/ or exp inactivated vaccine/ or live vaccine/ or exp meningitis vaccine/ or exp nucleic acid vaccine/ or exp parasite vaccine/ or exp peptide vaccine/ or protein vaccine/ or exp subunit vaccine/ or exp toxoid vaccine/ or exp vector vaccine/ or virosome vaccine/ or exp virus vaccine/ (304544)

60 vaccin*.ti,kw,kf. (251220)

61 immunization/ (104541)

For peer review only

62 passive immunization/ (12623)
63 (immunit* and transfer*).ti,kw,kf. (1262)
64 (immunit* and (maternally-acqui* or passive*)).ti,kw,kf. (766)
65 ((antibod* or anti-bod*) and transfer*).ti,kw,kf. (2375)
66 ((maternal or pregnan*) and immuni#ation*).ti,kw,kf. (1766)
67 exp malaria/pc [Prevention] (14654)
68 exp antimalarial agent/ (161880)
69 (antimalarial* or anti-malarial*).ti,kw,kf. (11586)
70 exp cytomegalovirus infection/pc [Prevention] (5146)
71 respiratory syncytial virus infection/pc [Prevention] (1057)
72 exp Streptococcus infection/pc [Prevention] (10568)
73 exp Zika fever/pc [Prevention] (780)
74 exp antiretrovirus agent/ (216196)
75 (antiretroviral* or anti-retroviral* or "anti-HIV" or "anti-AIDS" or "AIDS drug" or "AIDS drugs").ti,kw,kf. (46286)
76 exp fetus development/ (29871)
77 ((fetal or foetal or fetus* or foetus*) and develop*).ti,kw,kf. (14926)
78 exp prenatal exposure/ (37294)
79 ((fetal or foetal or fetus* or foetus* or prenatal* or pre-natal*) and expos*).ti,kw,kf. (16619)
80 pregnancy outcome/ (72177)
81 ((birth or births or matern* or neonat* or neo-nat* or perinatal* or peri-natal*or peripartum or "peri-partum" or postnatal* or post-natal* or postpartum or "post-partum" or post-birth* or pregnanc*) and outcome*).ti,kw,kf. (55800)
82 ((birth or births) and defect*).ti,kw,kf. (4849)
83 child health/ (32206)
84 maternal welfare/ (15842)
85 (MNCH and (maternal* or newborn* or child*)).ti,kw,kf. (49)
86 or/47-85 [DRUGS, VACCINES, SAFETY, OUTCOMES] (4592324)
87 46 and 86 [PERs, PREGNANCY - DATA COLLECTION, REGISTRIES - LMICs - DRUGS, VACCINES, SAFETY, OUTCOMES] (5663)
88 exp animal/ or exp animal experimentation/ or exp animal model/ or exp animal experiment/ or nonhuman/ or exp vertebrate/ (30677570)
89 exp human/ or exp human experimentation/ or exp human experiment/ (23737031)
90 88 not 89 (6941713)
91 87 not 90 [ANIMAL-ONLY REMOVED] (5643)
92 editorial.pt. (729310)
93 91 not 92 [EDITORIALS REMOVED] (5614)
94 limit 93 to yr="2000-current" (5299)

**************************

**CINAHL**

| # | Query | Results |
|---|---|---|
| S92 | S90 and S91 | 878 |
| S91 | DT 2000 - 2022 | 7,386,209 |

For peer review only

| | | |
|---|---|---|
| S90 | S88 NOT S89 | 885 |
| S89 | PT editorial or opinion or commentary | 684,750 |
| S88 | S45 AND S87 | 891 |
| S87 | S46 OR S47 OR S48 OR S49 OR S50 OR S51 OR S52 OR S53 OR S54 OR S55 OR S56 OR S57 OR S58 OR S59 OR S60 OR S61 OR S62 OR S63 OR S64 OR S65 OR S66 OR S67 OR S68 OR S69 OR S70 OR S71 OR S72 OR S73 OR S74 OR S75 OR S76 OR S77 OR S78 OR S79 OR S80 OR S81 OR S82 OR S83 OR S84 OR S85 OR S86 | 354,157 |
| S86 | TI (MNCH AND (maternal* OR newborn* OR child*)) | 17 |
| S85 | (MH "Maternal-Child Health") | 3,727 |
| S84 | (MH "Child Health") | 17,073 |
| S83 | TI (birth OR births) and defect* | 969 |
| S82 | TI (birth OR births OR matern* OR neonat* OR neo-nat* OR perinatal* OR peri-natal* OR peripartum OR "peri-partum" OR postnatal* OR post-natal* OR postpartum OR "post-partum" OR post-birth* OR pregnanc*) AND outcome* | 17,343 |
| S81 | (MH "Pregnancy Outcomes") | 27,127 |
| S80 | TI (fetal OR foetal OR fetus* OR foetus* or prenatal* or pre-natal*) AND expos* | 2,603 |
| S79 | (MH "Prenatal Exposure Delayed Effects") | 6,314 |
| S78 | TI (fetal OR foetal OR fetus* OR foetus*) AND develop* | 1,028 |
| S77 | (MH "Fetal Development") | 7,851 |
| S76 | TI antiretroviral* OR anti-retroviral* OR "anti-HIV" OR "anti-AIDS" OR "AIDS drug" OR "AIDS drugs" | 9,126 |
| S75 | (MH "Anti-Retroviral Agents+") | 24,911 |
| S74 | (MH "Zika Virus Infections/PC") | 168 |
| S73 | (MH "Streptococcal Infections+/PC") | 2,280 |
| S72 | (MH "Respiratory Syncytial Virus Infections/PC") | 583 |
| S71 | (MH "Hepatitis E/PC") | 69 |
| S70 | (MH "Cytomegalovirus Infections+/PC") | 509 |
| S69 | TI antimalarial* or (anti W0 malarial*) | 714 |
| S68 | (MH "Antimalarials+") | 8,320 |
| S67 | (MH "Malaria/PC") | 3,293 |

For peer review only

| | | |
|---|---|---|
| S66 | TI (maternal* or pregnan*) and immuni?ation* | 253 |
| S65 | TI ((antibod* or (anti W0 bod*)) and transfer*) | 79 |
| S64 | TI immunit* AND (passive* or "maternally-acquired") | 13 |
| S63 | TI immunit* AND transfer* | 16 |
| S62 | (MH "Immunity, Maternally Acquired") | 232 |
| S61 | (MH "Immunization Programs") | 6,426 |
| S60 | (MH "Viral Vaccines+") | 36,206 |
| S59 | (MH "Vaccines, Combined+") | 3,562 |
| S58 | (MH "Toxoids+") | 2,372 |
| S57 | (MH "Bacterial Vaccines+") | 10,296 |
| S56 | (MH "Vaccines") | 9,533 |
| S55 | (MH "Immunization+") | 31,914 |
| S54 | TI ( GAIA AND (alignment or immuni?ation or safety or pregnan*) ) OR AB ( GAIA AND (alignment or immuni?ation or safety or pregnan*) ) | 6 |
| S53 | TI ( "Global Alignment of Immunisation Safety Assessment in Pregnancy" or "Global Alignment of Immunization Safety Assessment in Pregnancy" ) OR AB ( "Global Alignment of Immunisation Safety Assessment in Pregnancy" or "Global Alignment of Immunization Safety Assessment in Pregnancy" ) | 0 |
| S52 | TI AEFI and adverse | 5 |
| S51 | TI "adverse effect" or "adverse effects" or "adverse reaction" or "adverse reactions" or "adverse event" or "adverse events" or "adverse outcome" or "adverse outcomes" | 12,615 |
| S50 | TI safe or safety or "side effect" or "side effects" or "undesirable effect" or "undesirable effects" or "treatment emergent" or tolerability or toxicity or adrs | 113,937 |
| S49 | TI (drug or drugs or medicine or medicines or medication* or pharmaceutical* or pharma-ceutical*) AND expos* | 1,420 |
| S48 | (MH "Adverse Drug Event") | 16,207 |
| S47 | TI (global or international* or world*) and health* | 22,194 |
| S46 | (MH "World Health") | 29,391 |
| S45 | S39 AND S44 | 4,402 |
| S44 | S40 OR S41 OR S42 OR S43 | 379,212 |

For peer review only

| | | |
|---|---|---|
| S43 | TI ( "high burden country" or "high burden countries" or "high-burden country" or "high-burden countries" or "countdown country" or "countdown countries" ) OR AB ( "high burden country" or "high burden countries" or "high-burden country" or "high-burden countries" or "countdown country" or "countdown countries" ) | 188 |
| S42 | TI ( "developing country" or "developing countries" or "developing nation" or "developing nations" or "developing population" or "developing populations" or "developing world" or "less developed country" or "less developed countries" or "less developed nation" or "less developed nations" or "less developed population" or "less developed populations" or "less developed world" or "lesser developed country" or "lesser developed countries" or "lesser developed nation" or "lesser developed nations" or "lesser developed population" or "lesser developed populations" or "lesser developed world" or "under developed country" or "under developed countries" or "under developed nation" or "under developed nations" or "under developed population" or "under developed populations" or "under developed world" or "underdeveloped country" or "underdeveloped countries" or "underdeveloped nation" or "underdeveloped nations" or "underdeveloped population" or "underdeveloped populations" or "underdeveloped world" or "middle income country" or "middle income countries" or "middle income nation" or "middle income nations" or "middle income population" or "middle income populations" or "low income country" or "low income countries" or "low income nation" or "low income nations" or "low income population" or "low income populations" or "lower income country" or "lower income countries" or "lower income nation" or "lower income nations" or "lower income population" or "lower income populations" or "underserved country" or "underserved countries" or "underserved nation" or "underserved nations" or "underserved population" or "underserved populations" or "underserved world" or "under served country" or "under served countries" or "under served nation" or "under served nations" or "under served population" or "under served populations" or "under served world" or "deprived country" or "deprived countries" or "deprived nation" or "deprived nations" or "deprived population" or "deprived populations" or "deprived world" or "poor country" or "poor countries" or "poor nation" or "poor nations" or "poor population" or "poor populations" or "poor world" or "poorer country" or "poorer countries" or "poorer nation" or "poorer nations" or "poorer population" or "poorer populations" or "poorer world" or "developing economy" or "developing economies" or "less developed economy" or "less developed economics" or "lesser developed economy" or "lesser developed economies" or "under developed economy" or "under developed economies" or "underdeveloped economy" or "underdeveloped economies" or "middle income economy" or "middle income economies" or "low income economy" or "low income economies" or "lower income economy" or "lower income economies" or "low gdp" or "low gnp" or "low gross domestic" or "low gross national" or "lower gdp" or "lower gnp" or "lower gross domestic" or "lower gross national" or lmic or lmics or "third world" or "lami country" or "lami | 39,219 |

For peer review only

countries" or "transitional country" or "transitional economies" or "emerging economy" or "emerging economies" or "emerging nation" or "emerging nations" ) OR AB ( "developing country" or "developing countries" or "developing nation" or "developing nations" or "developing population" or "developing populations" or "developing world" or "less developed country" or "less developed countries" or "less developed nation" or "less developed nations" or "less developed population" or "less developed populations" or "less developed world" or "lesser developed country" or "lesser developed countries" or "lesser developed nation" or "lesser developed nations" or "lesser developed population" or "lesser developed populations" or "lesser developed world" or "under developed country" or "under developed countries" or "under developed nation" or "under developed nations" or "under developed population" or "under developed populations" or "under developed world" or "underdeveloped country" or "underdeveloped countries" or "underdeveloped nation" or "underdeveloped nations" or "underdeveloped population" or "underdeveloped populations" or "underdeveloped world" or "middle income country" or "middle income countries" or "middle income nation" or "middle income nations" or "middle income population" or "middle income populations" or "low income country" or "low income countries" or "low income nation" or "low income nations" or "low income population" or "low income populations" or "lower income country" or "lower income countries" or "lower income nation" or "lower income nations" or "lower income population" or "lower income populations" or "underserved country" or "underserved countries" or "underserved nation" or "underserved nations" or "underserved population" or "underserved populations" or "underserved world" or "under served country" or "under served countries" or "under served nation" or "under served nations" or "under served population" or "under served populations" or "under served world" or "deprived country" or "deprived countries" or "deprived nation" or "deprived nations" or "deprived population" or "deprived populations" or "deprived world" or "poor country" or "poor countries" or "poor nation" or "poor nations" or "poor population" or "poor populations" or "poor world" or "poorer country" or "poorer countries" or "poorer nation" or "poorer nations" or "poorer population" or "poorer populations" or "poorer world" or "developing economy" or "developing economies" or "less developed economy" or "less developed economics" or "lesser developed economy" or "lesser developed economies" or "under developed economy" or "under developed economies" or "underdeveloped economy" or "underdeveloped economies" or "middle income economy" or "middle income economies" or "low income economy" or "low income economies" or "lower income economy" or "lower income economies" or "low gdp" or "low gnp" or "low gross domestic" or "low gross national" or "lower gdp" or "lower gnp" or "lower gross domestic" or "lower gross national" or lmic or lmics or "third world" or "lami country" or "lami countries" or "transitional country" or "transitional economies" or "emerging economy" or "emerging economies" or "emerging nation" or "emerging nations" )

For peer review only

| | | |
|---|---|---|
| S41 | TI ( mali or malta or micronesia or "federated states of micronesia" or kiribati or "marshall islands" or nauru or "northern mariana islands" or palau or tuvalu or mauritania or mauritius or mexico or moldova or moldovian or mongolia or montenegro or morocco or ifni or mozambique or "portuguese east africa" or myanmar or burma or namibia or nepal or "netherlands antilles" or nicaragua or niger or nigeria or oman or muscat or pakistan or panama or "papua new guinea" or "new guinea" or paraguay or peru or philippines or philipines or phillipines or phillippines or poland or "polish people's republic" or portugal or "portuguese republic" or "puerto rico" or romania or russia or "russian federation" or ussr or "soviet union" or "union of soviet socialist republics" or rwanda or ruanda or samoa or "pacific islands" or polynesia or "samoan islands" or "navigator island" or "navigator islands" or "sao tome and principe" or "saudi arabia" or senegal or serbia or seychelles or "sierra leone" or slovakia or "slovak republic" or slovenia or melanesia or "solomon island" or "solomon islands" or "norfolk island" or "norfolk islands" or somalia or "south africa" or "south sudan" or "sri lanka" or ceylon or "saint kitts and nevis" or "st. kitts and nevis" or "saint lucia" or "st. lucia" or "saint Vincent and the grenadines" or "saint vincent" or "st. vincent" or grenadines or sudan or suriname or surinam or "dutch guiana" or "netherlands guiana" or syria or "syrian arab republic" or tajikistan or tadjikistan or tadzhikistan or tadzhik or tanzania or tanganyika or thailand or siam or "timor leste" or "east timor" or togo or "togolese republic" or tonga or "Trinidad and tobago" or trinidad or tobago or tunisia or turkey or turkmenistan or turkmen or uganda or ukraine or uruguay or uzbekistan or uzbek or vanuatu or "new hebrides" or venezuela or vietnam or "viet nam" or "middle east" or "west bank" or gaza or palestine or yemen or yugoslavia or zambia or zimbabwe or "northern rhodesia" or "global south" or "africa south of the sahara" or "sub-saharan africa" or "subsaharan africa" or "africa, central" or "central africa" or "africa, northern" or "north africa" or "northern africa" or magreb or maghrib or sahara or "africa, southern" or "southern africa" or "africa, eastern" or "east africa" or "eastern africa" or "africa, western" or "west africa" or "western africa" or "west indies" or caribbean or "central america" or "latin america" or "south and central america" or "south america" or "asia, central" or "central asia" or "asia, northern" or "north asia" or "northern asia" or "asia, southeastern" or "southeastern asia" or "south eastern asia" or "southeast asia" or "south east asia" or "asia, western" or "western asia" or "europe, eastern" or "east europe" or "eastern europe" ) OR AB ( mali or malta or micronesia or "federated states of micronesia" or kiribati or "marshall islands" or nauru or "northern mariana islands" or palau or tuvalu or mauritania or mauritius or mexico or moldova or moldovian or mongolia or montenegro or morocco or ifni or mozambique or "portuguese east africa" or myanmar or burma or namibia or nepal or "netherlands antilles" or nicaragua or niger or nigeria or oman or muscat or pakistan or panama or "papua new guinea" or "new guinea" or paraguay or peru or philippines or philipines or phillipines or phillippines or poland or "polish people's republic" or portugal or "portuguese republic" or "puerto rico" or romania or russia or "russian federation" or ussr or "soviet union" or "union of soviet socialist republics" or rwanda or ruanda | 148,518 |

For peer review only - http://bmjopen.bmj.com/site/about/guidelines.xhtml

| | | |
|---|---|---|
| | or samoa or "pacific islands" or polynesia or "samoan islands" or "navigator island" or "navigator islands" or "sao tome and principe" or "saudi arabia" or senegal or serbia or seychelles or "sierra leone" or slovakia or "slovak republic" or slovenia or melanesia or "solomon island" or "solomon islands" or "norfolk island" or "norfolk islands" or somalia or "south africa" or "south sudan" or "sri lanka" or ceylon or "saint kitts and nevis" or "st. kitts and nevis" or "saint lucia" or "st. lucia" or "saint Vincent and the grenadines" or "saint vincent" or "st. vincent" or grenadines or sudan or suriname or surinam or "dutch guiana" or "netherlands guiana" or syria or "syrian arab republic" or tajikistan or tadjikistan or tadzhikistan or tadzhik or tanzania or tanganyika or thailand or siam or "timor leste" or "east timor" or togo or "togolese republic" or tonga or "Trinidad and tobago" or trinidad or tobago or tunisia or turkey or turkmenistan or turkmen or uganda or ukraine or uruguay or uzbekistan or uzbek or vanuatu or "new hebrides" or venezuela or vietnam or "viet nam" or "middle east" or "west bank" or gaza or palestine or yemen or yugoslavia or zambia or zimbabwe or "northern rhodesia" or "global south" or "africa south of the sahara" or "sub-saharan africa" or "subsaharan africa" or "africa, central" or "central africa" or "africa, northern" or "north africa" or "northern africa" or magreb or maghrib or sahara or "africa, southern" or "southern africa" or "africa, eastern" or "east africa" or "eastern africa" or "africa, western" or "west africa" or "western africa" or "west indies" or caribbean or "central america" or "latin america" or "south and central america" or "south america" or "asia, central" or "central asia" or "asia, northern" or "north asia" or "northern asia" or "asia, southeastern" or "southeastern asia" or "south eastern asia" or "southeast asia" or "south east asia" or "asia, western" or "western asia" or "europe, eastern" or "east europe" or "eastern europe" ) | |
| S40 | TI ( afghanistan or albania or algeria or "american samoa" or angola or antigua or barbuda or argentina or armenia or armenian or aruba or azerbaijan or bahrain or bangladesh or barbados or belarus or byelarus or belorussia or byelorussian or belize or "british honduras" or benin or dahomey or bhutan or bolivia or "bosnia herzegovina" or bosnia or herzegovina or botswana or bechuanaland or brazil or brasil or bulgaria or "burkina faso" or "burkina fasso" or "upper volta" or burundi or urundi or "cabo verde" or "cape verde" or cambodia or kampuchea or "khmer republic" or cameroon or cameron or cameroun or "central african republic" or "ubangi shari" or chad or chile or china or colombia or comoros or "comoro islands" or "iles comores" or mayotte or "democratic republic of the congo" or "democratic republic congo" or congo or zaire or "costa rica" or "cote d'ivoire" or "cote d' ivoire" or "cote divoire" or "cote d ivoire" or "ivory coast" or croatia or cuba or cyprus or "czech republic" or czechoslovakia or djibouti or "french somaliland" or dominica or "dominican republic" or ecuador or egypt or "united arab republic" or "el salvador" or "equatorial guinea" or "spanish guinea" or eritrea or estonia or eswatini or swaziland or ethiopia or fiji or gabon or "gabonese republic" or gambia or "georgia (republic)" or georgian or ghana or "gold coast" or gibraltar or greece or grenada or guam or guatemala or guinea or "guinea bissau" or guyana or "british guiana" or haiti or hispaniola or | 230,968 |

For peer review only

| | | |
|---|---|---|
| | honduras or hungary or india or indonesia or timor or iran or iraq or "isle of man" or jamaica or jordan or kazakhstan or kazakh or kenya or "democratic people's republic of korea" or "republic of korea" or "north korea" or "south korea" or korea or kosovo or kyrgyzstan or kirghizia or kirgizstan or "kyrgyz republic" or kirghiz or laos or "lao pdr" or "lao people's democratic republic" or latvia or lebanon or lebanese republic or lesotho or basutoland or liberia or libya or "libyan arab jamahiriya" or lithuania or macau or macao or republic of "north macedonia" or macedonia or madagascar or "malagasy republic" or malawi or nyasaland or malaysia or "malay federation" or "malaya federation" or maldives or "indian ocean islands" or "indian ocean" ) OR AB ( afghanistan or albania or algeria or "american samoa" or angola or antigua or barbuda or argentina or armenia or armenian or aruba or azerbaijan or bahrain or bangladesh or barbados or belarus or byelarus or belorussia or byelorussian or belize or "british honduras" or benin or dahomey or bhutan or bolivia or "bosnia herzegovina" or bosnia or herzegovina or botswana or bechuanaland or brazil or brasil or bulgaria or "burkina faso" or "burkina fasso" or "upper volta" or burundi or urundi or "cabo verde" or "cape verde" or cambodia or kampuchea or "khmer republic" or cameroon or cameron or cameroun or "central african republic" or "ubangi shari" or chad or chile or china or colombia or comoros or "comoro islands" or "iles comores" or mayotte or "democratic republic of the congo" or "democratic republic congo" or congo or zaire or "costa rica" or "cote d'ivoire" or "cote d' ivoire" or "cote divoire" or "cote d ivoire" or "ivory coast" or croatia or cuba or cyprus or "czech republic" or czechoslovakia or djibouti or "french somaliland" or dominica or "dominican republic" or ecuador or egypt or "united arab republic" or "el salvador" or "equatorial guinea" or "spanish guinea" or eritrea or estonia or eswatini or swaziland or ethiopia or fiji or gabon or "gabonese republic" or gambia or "georgia (republic)" or georgian or ghana or "gold coast" or gibraltar or greece or grenada or guam or guatemala or guinea or "guinea bissau" or guyana or "british guiana" or haiti or hispaniola or honduras or hungary or india or indonesia or timor or iran or iraq or "isle of man" or jamaica or jordan or kazakhstan or kazakh or kenya or "democratic people's republic of korea" or "republic of korea" or "north korea" or "south korea" or korea or kosovo or kyrgyzstan or kirghizia or kirgizstan or "kyrgyz republic" or kirghiz or laos or "lao pdr" or "lao people's democratic republic" or latvia or lebanon or lebanese republic or lesotho or basutoland or liberia or libya or "libyan arab jamahiriya" or lithuania or macau or macao or republic of "north macedonia" or macedonia or madagascar or "malagasy republic" or malawi or nyasaland or malaysia or "malay federation" or "malaya federation" or maldives or "indian ocean islands" or "indian ocean" ) | |
| S39 | S1 OR S38 | 15,244 |
| S38 | S16 AND S37 | 15,243 |
| S37 | S17 OR S18 OR S19 OR S20 OR S21 OR S22 OR S23 OR S24 OR S25 OR S26 OR S27 OR S28 OR S29 OR S30 OR S31 OR S32 OR S33 OR S34 OR S35 OR S36 | 354,620 |

For peer review only

| | | |
|---|---|---|
| S36 | (MH "Product Surveillance+/SN") | 1 |
| S35 | (MH "Maternal Health Services+/SN") | 1,245 |
| S34 | TI pharmacovigilan* or (pharmaco W0 vigilan*) | 846 |
| S33 | (MH "Product Surveillance+") | 2,417 |
| S32 | TI "data system" or "data systems" or "information system" or "information systems" | 4,466 |
| S31 | (MH "Health Information Systems") | 3,566 |
| S30 | TI (decision* AND support* AND clinical*) | 1,865 |
| S29 | (MH "Decision Support Systems, Clinical") | 6,139 |
| S28 | TI surveillance* | 15,975 |
| S27 | (MH "Population Surveillance+") | 11,277 |
| S26 | TI survey* | 71,609 |
| S25 | (MH "Surveys") | 157,567 |
| S24 | TI registry or registries or eregistr* or "e-registry" or "e-registries" | 13,946 |
| S23 | TI (preliminary or "pilot project" or "pilot projects") AND data | 682 |
| S22 | TI "focus group" or "focus groups" | 2,957 |
| S21 | (MH "Focus Groups") | 48,414 |
| S20 | TI databas* or "data base" or "data bases" or databank* or "data bank" or "data banks" or dataset* or "data set" or "data sets" | 16,019 |
| S19 | TI data AND (accumulat* or accura* or assembl* or captur* or collect* or compil* or coordinat* or co-ordinat* or gather* or hub or hubs) | 4,425 |
| S18 | (MH "Data Curation") | 184 |
| S17 | (MH "Data Collection") | 49,942 |
| S16 | S2 OR S3 OR S4 OR S5 OR S6 OR S7 OR S8 OR S9 OR S10 OR S11 OR S12 OR S13 OR S14 OR S15 | 290,175 |
| S15 | TI maternal AND (fetal or fetus or foetal or foetus) | 4,004 |
| S14 | TI (maternal* or maternity) AND (care or health* or service or services) | 9,718 |
| S13 | (MH "Maternal Health Services") | 11,199 |
| S12 | TI (maternal* or maternity) AND (contact* or expos*) | 1,028 |
| S11 | (MH "Maternal Exposure") | 2,588 |

For peer review only

| | | |
|---|---|---|
| S10 | TI perinatal* or "peri-natal" or "peri-natally" or peripartum or "peri-partum" | 12,858 |
| S9 | (MH "Perinatal Care") | 5,019 |
| S8 | TI prenatal* or antenatal* or "ante-natal" or "ante-natally" or antepartum or "ante-partum" | 20,877 |
| S7 | (MH "Prenatal Care") | 19,422 |
| S6 | TI pregnan* | 80,123 |
| S5 | (MH "Pregnancy Trimesters+") | 13,521 |
| S4 | (MH "Expectant Mothers") | 11,382 |
| S3 | (MH "Pregnancy Complications+") | 106,825 |
| S2 | (MH "Pregnancy") OR (MH "Pregnancy, Multiple+") OR (MH "Pregnancy, Unplanned") OR (MH "Pregnancy, Unwanted") OR (MH "Pregnancy, High Risk") OR (MH "Pregnancy, Prolonged") | 227,240 |
| S1 | TI ( "pregnancy exposure database" OR "pregnancy exposure databases" OR "pregnancy exposure data base" OR "pregnancy exposure data bases" OR "pregnancy exposure registry" OR "pregnancy exposure registries" ) OR AB ( "pregnancy exposure database" OR "pregnancy exposure databases" OR "pregnancy exposure data base" OR "pregnancy exposure data bases" OR "pregnancy exposure registry" OR "pregnancy exposure registries" ) | 7 |

**Global Index Medicus**

tw:((tw:(pregnan* exposure* database*)) OR (tw:(pregnan* exposure* "data base")) OR (tw:(pregnan* exposure* "data bases")) OR (tw:(pregnan* exposure* databank*)) OR (tw:(pregnan* exposure* "data bank")) OR (tw:(pregnan* exposure* "data banks")) OR (tw:(pregnan* exposure* register)) OR (tw:(pregnan* exposure* registries)) OR (tw:(pregnan* exposure* registry)) OR (tw:(pregnan* exposure* registries))) – *108 records*

tw:((ti:((pregnan* OR prenatal* OR antenatal* OR "ante-natal" OR "ante-natally" OR antepartum OR "ante-partum" OR perinatal* OR "peri-natal" OR "peri-natally" OR peripartum OR "peri-partum" OR maternal*) AND (data OR database* OR databank* OR register OR registers OR registry OR registries OR survey* OR surveillance OR pharmacovigilan* OR "pharmaco-vigilance") AND (safe OR safety OR "side effect" of "side effects" OR "undesirable effect" OR "undesirable effects" OR "treatment emergent" OR tolerability OR toxicity OR adrs OR aefi)))) – *361 records*

tw:(ti:((pregnan* OR prenatal* OR antenatal* OR "ante-natal" OR "ante-natally" OR antepartum OR "ante-partum" OR perinatal* OR "peri-natal" OR "peri-natally" OR peripartum OR "peri-partum" OR maternal*) AND (data OR database* OR databank* OR register OR registers OR registry OR registries OR survey* OR surveillance OR pharmacovigilan* OR "pharmaco-vigilance") AND (MNCH or "maternal health" or "child health" or "infant health" or "birth defect" or "birth defects" or "birth outcome" or

For peer review only

"birth outcomes" or "pregnancy outcome" or "pregnancy outcomes" or "neonatal outcome" or "neonatal outcomes"))) – *50 records*

tw:(ti:((pregnan* OR prenatal* OR antenatal* OR "ante-natal" OR "ante-natally" OR antepartum OR "ante-partum" OR perinatal* OR "peri-natal" OR "peri-natally" OR peripartum OR "peri-partum" OR maternal*) AND (data OR database* OR databank* OR register OR registers OR registry OR registries OR survey* OR surveillance OR pharmacovigilan* OR "pharmaco-vigilance") AND (vaccine or vaccines or vaccination* or immunization* or immunization* or “maternally-acquired”))) – *0 records*

tw:((ti:((pregnan* OR prenatal* OR antenatal* OR "ante-natal" OR "ante-natally" OR antepartum OR "ante-partum" OR perinatal* OR "peri-natal" OR "peri-natally" OR peripartum OR "peri-partum" OR maternal*) AND (data OR database* OR databank* OR register OR registers OR registry OR registries OR survey* OR surveillance OR pharmacovigilan* OR "pharmaco-vigilance") AND (drug OR drugs OR medicine OR medicines OR medication* OR pharmaceutical* OR "pharma-ceutical" OR "pharma-ceuticals" OR vaccin* OR immun* OR antimalarial* OR "anti-malarial" OR "anti-malarials" OR antiviral* OR "anti-viral" OR "anti-virals" OR antiretroviral* OR "anti-retroviral" OR "anti-retrovirals" OR "Anti-HIV" OR "Anti-AIDS")))) – *10 records*

tw:((ti:((pregnan* OR prenatal* OR antenatal* OR "ante-natal" OR "ante-natally" OR antepartum OR "ante-partum" OR perinatal* OR "peri-natal" OR "peri-natally" OR peripartum OR "peri-partum" OR maternal*) AND (data OR database* OR databank* OR register OR registers OR registry OR registries OR survey* OR surveillance OR pharmacovigilan* OR "pharmaco-vigilance") AND (fetal* OR foetal* OR fetus* OR foetus* OR neonat* OR newborn* OR infant OR infants OR infanc* OR child*) ))) – *125 records*

tw:((ti:((pregnan* OR prenatal* OR antenatal* OR "ante-natal" OR "ante-natally" OR antepartum OR "ante-partum" OR perinatal* OR "peri-natal" OR "peri-natally" OR peripartum OR "peri-partum" OR maternal*) AND (data OR database* OR databank* OR register OR registers OR registry OR registries OR survey* OR surveillance OR pharmacovigilan* OR "pharmaco-vigilance"))) AND (tw:(drug OR drugs OR medicine OR medicines OR medication* OR pharmaceutical* OR "pharma-ceutical" OR "pharma-ceuticals" OR vaccin* OR immunisation* OR immunization* OR antimalarial* OR "anti-malarial" OR "anti-malarials" OR antiviral* OR "anti-viral" OR "anti-virals" OR antiretroviral* OR "anti-retroviral" OR "anti-retrovirals" OR "Anti-HIV" OR "Anti-AIDS"))) – *71 records*

**TOTAL: 725 records**

## Google Scholar

"pregnant|pregnancy|prenatal"+"registry|registries|surveillance|pharmacovigilance" +

"expose|exposed|exposes|exposure|exposures"+[names of LMICs]

For peer review only - http://bmjopen.bmj.com/site/about/guidelines.xhtml

# Data Extraction Form

# PART 1: STUDY IDENTIFICATION / INCLUSION STATUS

Resource title:

Author(s):

Year of publication:

Resource type:

1. 1 - Peer reviewed journal
2. 2 - Grey literature
3. 3 - Registry
4. 4 - Website
5. 5 - Key informant survey / interview
6. Other

Database resource pulled from:

Final status:

1. 1 - included
2. 2 - excluded
3. 3 - incomplete/unclear

If excluded, explain reasoning:

For peer review only

# PART 2: REGISTRY INFORMATION OR OTHER RESOURCE

Name of registry or other resource:

Primary goals/aims:

Funding source:

Years of operation:

Current status

1. Active
2. Inactive
3. On hold

Country(ies) where located:

Country representativeness:

1. Multi-national
2. National
3. State/Provincial
4. District
5. Sub-district
6. Community
7. Health clinic
8. Hospital
9. Other

Drug/vaccine exposure name and type:

Duration of follow up:

For peer review only

Current sample size:

1. <500 participants
2. 500 - 1,999 participants
3. 2,000 - 5,000 participants
4. 5,001 - 10,000 participants
5. 10,001 - 20,000 participants
6. >20,000 participants

Data collection:

1. Administrative databases
2. Medical databases
3. Registries
4. Research study
5. Program implementation
6. Other

Methodology:

Terminology and data system used:

# PART 3: CHARACTERISTICS OF INCLUDED POPULATION

Age ranges included:

Target population:

1. Pregnant women
2. Non-pregnant women / women of reproductive age
3. Children
4. Infants (<28 days old)
5. General population
6. Other

For peer review only

Gestational age

1. First trimester
2. Second trimester
3. Third trimester

Underlying medical condition(s):

Maternal outcome(s) recorded:

1. Thrombosis and/or thrombocytopenia syndrome
2. Antenatal hospitalization (not including delivery)
3. Disability
4. Gestational diabetes
5. Gestational hypertension
6. Premature rupture of membranes
7. Preterm labor
8. Preeclampsia / Eclampsia
9. Post-partum hemorrhage
10. Antenatal hemorrhage
11. Corporeal infection
12. Spontaneous abortion / miscarriage / pregnancy loss (example: prior to 20 weeks gestation)
13. Maternal death (example: within 42 days of termination of pregnancy)
14. Late maternal death (example: >42 days of termination of pregnancy)
15. Other

Maternal death cause:

1. Direct cause
2. Indirect cause

Neonatal outcome(s) recorded:

1. Preterm birth
2. Small size for gestational age / restricted fetal growth
3. Still birth (death after 28 weeks of pregnancy but before birth)
4. Live birth
5. Congenital anomaly / birth defect
6. Death
7. Other

For peer review only

Neonatal death timeframe

1. Early neonatal (0-7 days)
2. Late neonatal (8-28 days)
3. Post neonatal (29 days - 1 year)

Which congenital anomaly does this monitor (if any)?

Infant/child outcome(s) recorded:

1. Infections
2. Respiratory illness
3. Developmental outcomes
4. Other

What is the duration of follow up?

# PART 4: KEY FINDINGS

# Populate as best you can if information is provided; answer N/A if it is not relevant or N/P if it is not provided.

Strengths of the registry or other resource:

Weaknesses / gaps of registry or other resource:

Challenges of registry or other resource in its specific context:

Does this resource have the possibility to add new interventions?

1. Yes
2. No
3. Maybe
4. Unknown

For peer review only - http://bmjopen.bmj.com/site/about/guidelines.xhtml

Why?

For peer review only

Can this resource be combined with other systems?

1. Yes
2. No
3. Maybe
4. Unknown

Why?

Any upcoming changes to the resource:

Additional comments

For peer review only
